# Steroidomic Differences Among Clozapine, Olanzapine, and Risperidone in Male Patients with First-Episode Psychosis, Including a Treatment-Resistant Schizophrenia Subgroup

**DOI:** 10.3390/ijms27198909

**Published:** 2026-10-07

**Authors:** Karel Valeš, Kristína Holubová, Filip Španiel, Petra Fürstová, Miloš Petrović, Marta Velíková, Martin Hill

**Affiliations:** 1 National Institute of Mental Health, Topolova 748, 250 67 Klecany, Czech Republic; karel.vales@nudz.cz (K.V.); kristina.holubova@nudz.cz (K.H.); filip.spaniel@nudz.cz (F.Š.);; 2 Third Faculty of Medicine, Charles University in Prague, Ruská, 871 00 Prague, Czech Republic; 3 Clinic for Internal Medicine, Clinical Hospital Centre, Dragisa Misovic-Dedinje, 11000 Belgrade, Serbia; milos.petrovic@dragisamisovic.bg.ac.rs; 4 Institute of Endocrinology, 110 00 Prague, Czech Republic

**Keywords:** schizophrenia, clozapine, risperidone, olanzapine, neuroactive steroids, GABAA modulation, first-episode psychosis, treatment resistant schizophrenia

## Abstract

Clozapine (Clo) is the most effective antipsychotic for treatment-resistant schizophrenia (TRS), yet the mechanisms underlying its unique clinical profile remain insufficiently understood. Neuroactive steroids (NASs) influence neuronal excitability, neuroplasticity, inflammation, and stress responses—domains disrupted in schizophrenia—and may contribute to antipsychotic pharmacodynamics. This pilot study examined whether Clo exhibits a distinctive steroidomic pattern compared with olanzapine (Ola) and risperidone (Ris) in male patients with first-episode psychosis, including a clinically defined TRS subgroup receiving Clo. Serum collected after group-specific treatment durations (Clo: 17.7 ± 12.4; Ola: 13.9 ± 1.9; Ris: 12.9 ± 1.3 months) was analyzed by GC–MS/MS, with steroidogenic enzyme activities inferred from precursor–product molar ratios as proxies of metabolic balance. Statistical evaluation included age-adjusted ANCOVA, OPLS, multiple regression, and trend-based analysis of coordinated pathway-level shifts. Although Ola increased several steroid concentrations, its overall and unconjugated trends did not differ significantly from Clo (*p* = 0.515 and *p* = 0.409). At the multivariate level, however, the two profiles were significantly discriminated (OPLS: R^2^Y = 50.8%, CV-ANOVA *p* = 0.011), indicating that the difference lies in the coordinated pattern rather than the global magnitude of change. Clo showed a distinctive exploratory pattern: lower DHEA, higher 5α-reduced pregnane NAS, altered androgen turnover, reduced *CYP17A1* lyase proxy activity, and a mild but statistically significant increase in the cortisol-to-cortisone ratio, and was clearly distinguished from Ris by broad steroid increases. This may indicate a shift toward a profile with potential GABAergic and neuroprotective features, although interpretations remain exploratory given the small, cross-sectional, male-only sample and the non-independence of steroid pathways. Neurosteroidogenesis may thus contribute to antipsychotic response variability and offer candidate biomarkers, warranting validation in larger longitudinal studies.

## 1. Introduction

Clozapine (Clo) is the gold-standard treatment for therapy-resistant schizophrenia (TRS), yet its mechanism of action remains only partially understood. Clo interacts with multiple neurotransmitter systems, including dopaminergic (D1–D5), serotonergic (5-HT1A, 5-HT2A/2C, 5-HT6/7), glutamatergic (NMDA, mGluR3), GABAergic (GABA_A_), muscarinic (M1–M5), adrenergic (α1, α2), and histaminergic (H1) receptors [1,2], but even this unusually broad pharmacodynamic profile does not fully account for its distinctive clinical efficacy. Beyond classical neurotransmitter signaling, Clo has been shown to influence neuronal excitability [3,4,5,6,7], enhance synaptic plasticity [8,9], promote neurogenesis [10], reduce inflammation [11,12] and oxidative stress [13], restore mitochondrial function [14], and improve functional connectivity [15], indicating engagement of broader regulatory systems. Neuroactive steroids (NASs) represent one such system of particular interest, as they mechanistically link several of these domains—excitability, plasticity, stress, and inflammation—and, because many NASs cross the blood–brain barrier, they provide a measurable peripheral readout that may partially reflect central neurosteroidogenic activity relevant to antipsychotic action.

NASs modulate neuronal excitability through GABA_A_ and NMDA receptors [16,17], support neuroplasticity [18], influence functional connectivity [19], and regulate inflammatory and oxidative processes [20,21,22]. Altered NAS profiles have been reported in schizophrenia [23,24,25,26,27,28], and our previous work demonstrated significant differences between patients and healthy controls, with disease progression exerting stronger effects on NAS levels than antipsychotic treatment when medications were analyzed collectively [29]. Examples of NASs relevant to schizophrenia include allopregnanolone (a potent GABA_A_ positive modulator), pregnenolone (linked to neuroplasticity and stress regulation), and dehydroepiandrosterone (DHEA), which exhibits concentration-dependent excitatory actions.

Building on these findings, the present study re-analyzed serum samples from male first-episode psychosis (FEP) patients, including a clinically defined TRS subgroup receiving Clo, after group-specific treatment durations with Clo, olanzapine (Ola), or risperidone (Ris). The aim was to evaluate exploratory pharmacodynamic differences in steroidomic profiles across these three antipsychotics. By integrating concentration changes, proxy enzymatic ratios, and pathway-level trends, we tested the hypothesis that Clo exhibits a distinctive and internally coordinated steroidomic pattern—rather than merely a difference in the global magnitude of steroidogenic change—that may be relevant to its therapeutic profile in TRS. Given the small, cross-sectional, male-only sample and the non-independence of steroidogenic pathways, the findings are presented as exploratory and hypothesis-generating.

## 2. Results

### 2.1. Patients’ Characteristics

The three groups did not differ significantly in age or duration of untreated psychosis (DUP). Although the omnibus test for age did not reach conventional significance (ANOVA *p* = 0.069), standardized pairwise comparisons revealed a large Clo–Ris contrast (Hedges’ g = −1.28), indicating a tendency toward older age in the risperidone group. The groups were not clinically equivalent by design, as the clozapine group was defined by treatment-resistant schizophrenia (TRS), whereas the olanzapine and risperidone groups were not (Table 1). Duration of illness (DI) did not reach conventional significance (ANOVA *p* = 0.061) but showed a large effect size (ηp^2^ = 0.225), with a significant Clo–Ola contrast, indicating a tendency toward longer illness duration in the clozapine group (Section 6, Table 13). Despite the absence of statistically significant age differences, the risperidone group was older on average, and the clozapine group represented a clinically more severe, treatment-resistant subgroup, which may influence endocrine and metabolic parameters independently of treatment assignment. This relative diagnostic homogeneity at baseline (where all patients presented with first-episode psychosis) reduces the likelihood that V2 steroid differences were driven primarily by baseline illness severity rather than treatment exposure, although residual confounding related to TRS status and divergent clinical trajectories cannot be excluded.

Body mass index (BMI) increased significantly from V1 to V2 only in the Ola group (within-group randomization test, *p* < 0.01), consistent with its well-established metabolic impact [23,24], and may partially account for the androgen-related steroid elevations observed in Ola-treated patients. Given the small sample sizes, particularly in the Clo (*n* = 6) and Ris (*n* = 5) groups, these clinical observations should be interpreted with caution.

Treatment duration at V2 was longest in the Clo group, with a significant between-group difference (ANOVA *p* = 0.014, ηp^2^ = 0.335). Cumulative chlorpromazine equivalents (cCPZeq) also differed significantly between groups at V2 (*p* = 0.020), and the V1–V2 change (Δ) in cCPZeq diverged between groups (ANOVA on Δ: *p* = 0.020, ηp^2^ = 0.323), reflecting an increase in Clo-treated individuals versus a decrease in the Ola and Ris groups. These findings indicate more sustained and intensive antipsychotic exposure in the Clo group, as reflected by higher cumulative CPZeq and longer treatment duration, which may itself influence steroid metabolism and should be considered a potential confounder rather than a pure treatment effect.

Clinically, symptomatic and functional trajectories showed group-level differences primarily in between-group contrasts rather than in within-group V1–V2 changes, none of which reached significance in the paired randomization test. The Ris group showed the numerically largest reductions across PANSS total, general, and negative subscales, although none reached within-group significance, and between-group contrasts distinguished Ris from Ola on PANSS-negative and PANSS total scores (‡). The absence of a significant within-group PANSS change in the Clo group is consistent with the treatment-resistant schizophrenia (TRS) phenotype typically associated with Clo initiation. Functional outcomes measured by the Global Assessment of Functioning (GAF) differed significantly between groups at V2 (ANOVA *p* = 0.045, ηp^2^ = 0.246), with higher functioning in the Ola and Ris groups relative to Clo; within-group V1–V2 gains did not reach significance in any group.

Together, these clinical characteristics provide essential context for interpreting steroidomic patterns, which may reflect a combination of drug-specific effects and underlying differences in illness severity, treatment history, and metabolic status. Given the small sample sizes and the non-randomized, naturalistic design, these clinical differences should be viewed as exploratory and hypothesis-generating rather than definitive group-level distinctions. The Clo group corresponded to a clinically more severe, treatment-resistant phenotype, with longer exposure and higher cumulative dosing; the Ola group was characterized by metabolic sensitivity (significant BMI gain and prolactin change); and the Ris group by the largest, though not individually significant, symptomatic reductions.

After adjustment for the baseline and change in PANSS and GAF, and for cumulative CPZeq exposure and treatment duration (Supplementary Table S1), the group differences in steroid profiles differed between contrasts: both clozapine contrasts (vs. olanzapine and vs. risperidone) remained statistically significant across all adjustment models, whereas the olanzapine–risperidone discrimination did not reach significance in any model (see Section 2.2 and Supplementary Table S1).

### 2.2. Global Steroidomic Differences

Age-adjusted ANCOVA revealed significant differences in multiple steroids, including 5α-pregnane-3α,20α-diol, cortisone, DHEA and several downstream androgen metabolites such as androstenedione, epiandrosterone and 5β-androstane-3α,17β-diol (Supplementary Table S2). A complete list of all 85 quantified steroids, including their full biochemical names and metabolic grouping, is provided in Table 2, and baseline concentrations for these analytes are summarized in Supplementary Table S3.

However, because ANCOVA was applied to 85 steroids (Table 2) without a global correction for multiple testing, the unadjusted results should be interpreted as exploratory. To address multiplicity, exact *p*-values for the drug effect were recalculated from the reported F statistics and subjected to Benjamini–Hochberg false discovery rate (BH-FDR) correction at α = 0.05, applied separately within predefined biochemical blocks of native steroids and enzyme-proxy activity ratios, as detailed in Supplementary Table S2.

**Figure 1:**
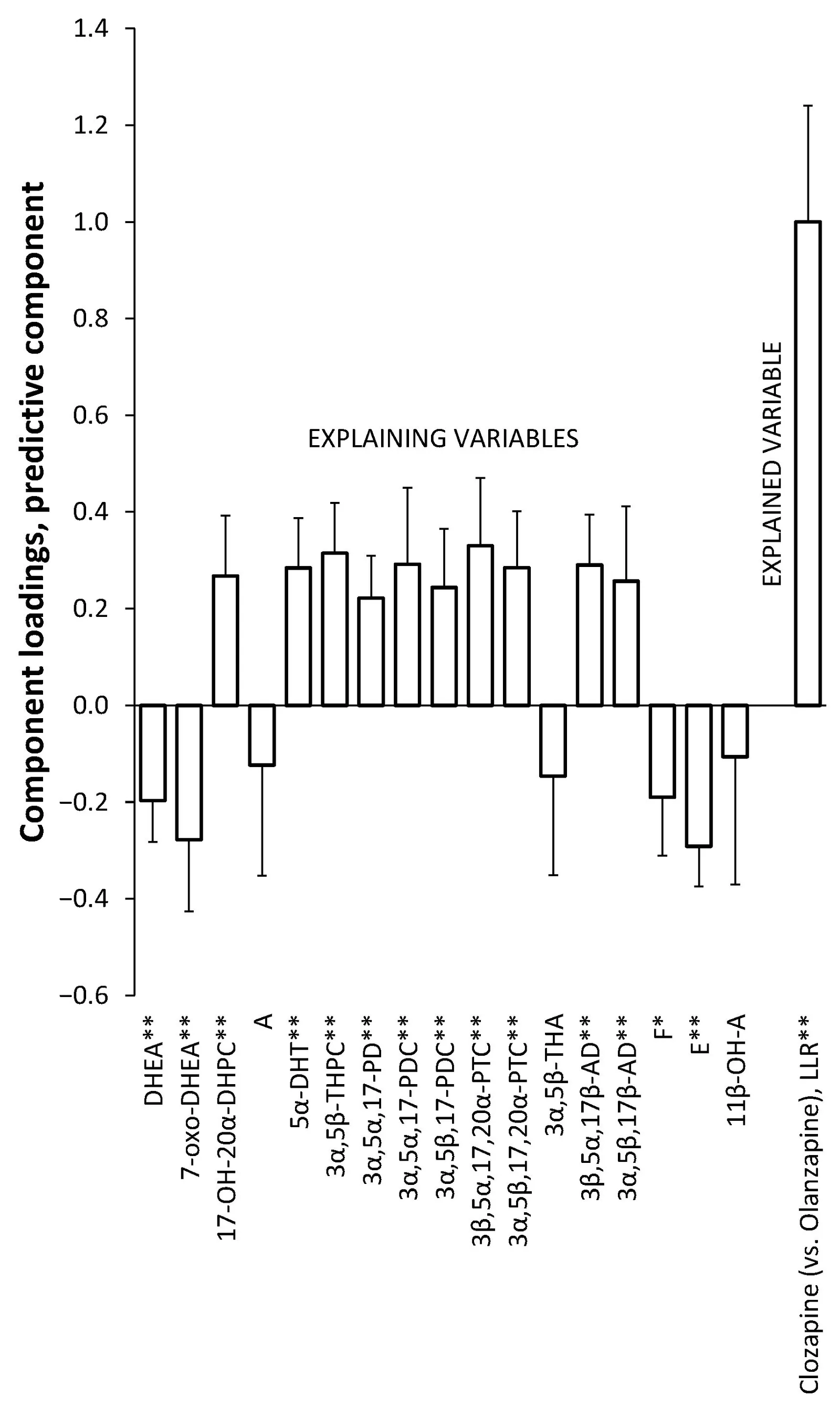
Loading plot of the predictive component of the OPLS model discriminating clozapine- from olanzapine-treated patients based on steroid profiles (corresponding to Table 3). Bars represent component loadings expressed as correlation coefficients (R) of the individual explanatory variables (steroids, X) with the predictive component; the explained variable (Y) is the group classification of clozapine vs. olanzapine (LLR). Positive loadings indicate elevated steroids in the clozapine group; negative loadings indicate reduced steroids in the clozapine group relative to olanzapine. Error bars denote 95% confidence intervals (jackknife-based); loadings whose confidence intervals exclude zero represent variables significantly contributing to group separation. Model performance: R^2^Y = 50.8% (Q^2^ = 40.7%); CV-ANOVA F(2,17) = 5.9; *p* = 0.011; 17 of 86 variables were relevant; predictive/orthogonal components = 1/0. Asterisks indicate the statistical significance of each variable: * *p* < 0.05, ** *p* < 0.01. Abbreviations: OPLSs—orthogonal projections to latent structures; LLR—logarithm of the likelihood ratio; CV—cross-validation; CV-ANOVA—analysis of variance of cross-validated predictive residuals. Steroid abbreviations: DHEA—dehydroepiandrosterone; 7-oxo-DHEA—7-oxo-dehydroepiandrosterone; 17-OH-20α-DHPC—conjugated 17-hydroxy-20α-dihydroprogesterone; A—androstenedione; 5α-DHT—5α-dihydrotestosterone; 3α,5β-THPC—conjugated 3α,5β-tetrahydroprogesterone; 3α,5α,17-PD—3α,5α,17-pregnanediol; 3α,5α,17-PDC—conjugated 3α,5α,17-pregnanediol; 3α,5β,17-PDC—conjugated 3α,5β,17-pregnanediol; 3β,5α,17,20α-PTC—conjugated 3β,5α,17,20α-pregnanetetrol; 3α,5β,17,20α-PTC—conjugated 3α,5β,17,20α-pregnanetetrol; 3α,5β-THA—3α,5β-tetrahydro androstenedione; 3β,5α,17β-AD—3β,5α,17β-androstanediol; 3α,5β,17β-AD—3α,5β,17β-androstanediol; F—cortisol; E—cortisone; 11β-OH-A—11β-hydroxyandrostenedione.

**Figure 2:**
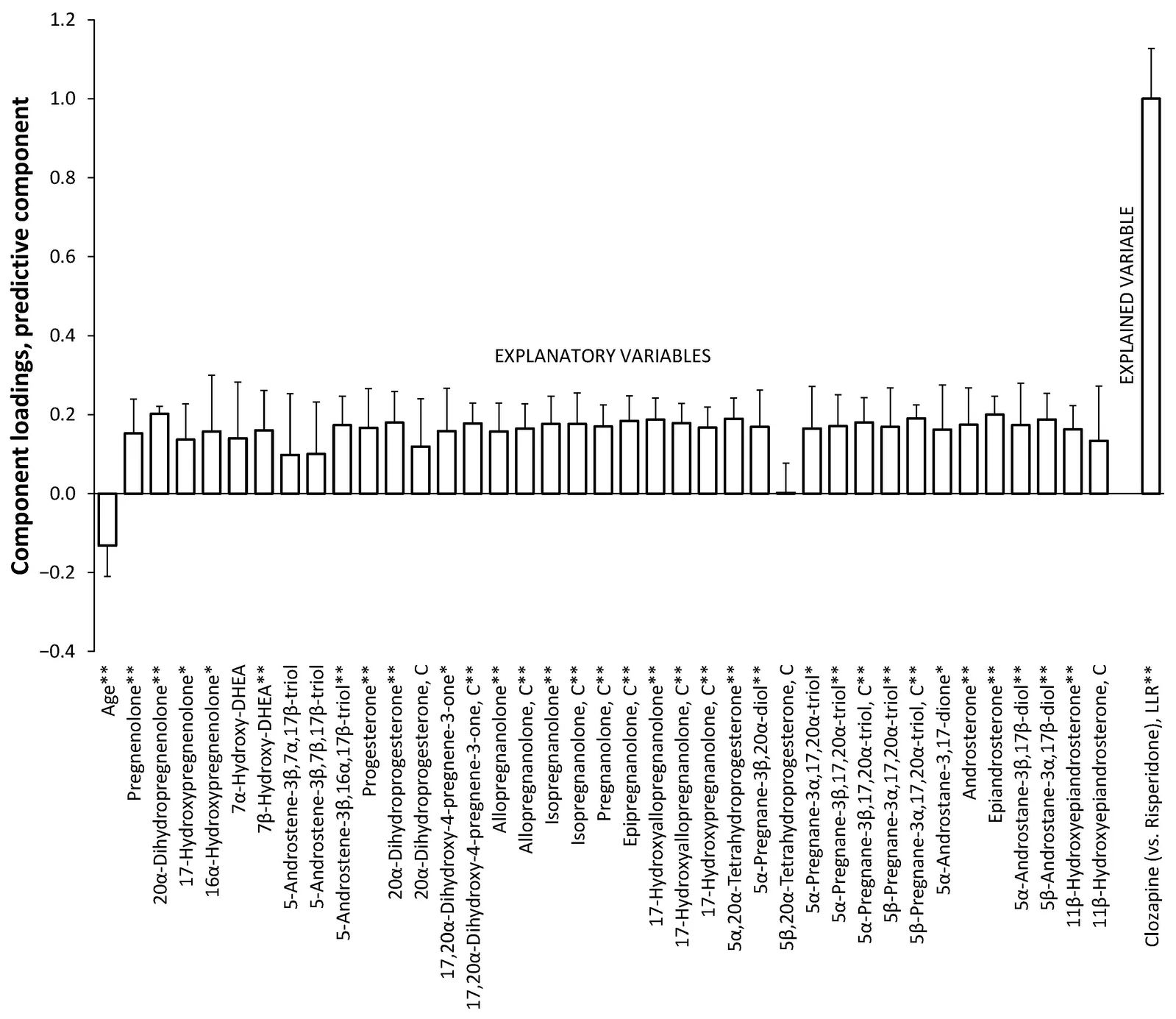
Loading plot of the predictive component of the OPLS model discriminating clozapine- from risperidone-treated patients based on steroid profiles (corresponding to Table 7). Bars represent component loadings expressed as correlation coefficients (R) of the individual explanatory variables (steroids and age, X) with the predictive component; the explained variable (Y) is the group classification of clozapine vs. risperidone (LLR). Positive loadings indicate variables elevated in the clozapine group; negative loadings indicate variables reduced in the clozapine group relative to risperidone. Error bars denote 95% confidence intervals (jackknife-based); loadings whose confidence intervals exclude zero represent variables significantly contributing to group separation. Model performance: R^2^Y = 62.8% (Q^2^ = 56.2%); CV-ANOVA F(2,8) = 5.1; *p* = 0.037; 39 of 86 variables were relevant; predictive/orthogonal components = 1/0. Asterisks indicate the statistical significance of each variable: * *p* < 0.05, ** *p* < 0.01. Abbreviations: OPLS—orthogonal projections to latent structures; LLR—logarithm of the likelihood ratio; CV—cross-validation; CV-ANOVA—analysis of variance of cross-validated predictive residuals; C—conjugated form; DHEA—dehydroepiandrosterone.

OPLS/OMR discrimination models are presented sequentially for each treatment comparison, beginning with Clo–Ola, followed by Clo–Risp, and finally Ola–Risp (Table 3, Table 4, Table 5, Table 6, Table 7, Table 8, Table 9, Table 10 and Table 11), which report only models with significant CV-ANOVA (*p* < 0.05). Models showing a significant predictive component, but non-significant CV-ANOVA are provided separately in Supplementary Table S4. Multivariate OPLS and OMR analyses revealed pattern-level discrimination involving approximately 38 steroids for clozapine versus olanzapine and 39 for clozapine versus risperidone (Table 3, Figure 1 and Table 7, Figure 2, respectively). These counts represent the number of variables contributing positively to model-based group separation rather than confirmed increases in absolute steroid concentrations. Given the small sample sizes in the clozapine (*n* = 6) and risperidone (*n* = 5) groups, together with the high dimensionality of the steroidomic dataset, these multivariate models should be interpreted as exploratory. Although they provide internally consistent discrimination patterns, the risk of overfitting remains substantial, and the findings are therefore intended to guide biological interpretation rather than to support definitive inferential claims. Several OPLS models showed reduced or even negative cross-validated explained variance (Q^2^), indicating limited generalizability of the predictive component.

Models with a significant predictive component but non-significant CV-ANOVA are reported in Supplementary Table S4; for many of these, the CV-ANOVA *p*-values were less than 0.1, frequently around *p* = 0.06, consistent with borderline predictive stability in small pilot samples. Taken together, these results indicate that OPLS-derived discrimination patterns should be regarded as hypothesis-generating rather than confirmatory. It should be noted that the steroid counts derived from the OPLS/OMR analyses (Table 3, Table 4, Table 5, Table 6, Table 7, Table 8, Table 9, Table 10 and Table 11 and Supplementary Table S4) and those obtained from the trend-based pathway test (Table 12) are not directly comparable, as the two approaches apply fundamentally different counting frameworks. In the multivariate models, counts reflect the number of variables contributing to model-based discrimination, whereas the trend-based procedure aggregates directional shifts across the entire steroidogenic network. Moreover, individual steroids, their conjugated forms, and precursor–product ratios are not statistically independent observations; many share metabolic pathways and exhibit strong intercorrelations. As a result, the number of “elevated steroids” should not be interpreted as the number of independent pathway-level findings. The trend-based pathway analysis confirmed broad increases in steroidogenesis for clozapine versus risperidone and olanzapine versus risperidone (Table 12), indicating coordinated upregulation across steroidogenic pathways. Because precursor–product ratios can be influenced by adrenal and gonadal secretion, hepatic metabolism, binding proteins, circadian variation, and parallel enzymatic routes, they represent proxy indicators of metabolic balance rather than direct measures of enzyme activity. Accordingly, pathway-level interpretations should be made with caution.

In sensitivity analyses, baseline PANSS-P was added to the steroid predictors in the OPLS models. For the clozapine–olanzapine contrast, the model remained significant after adjustment (R^2^Y = 45.4%, Q^2^ = 31.7%, CV-ANOVA F = 4.0, *p* = 0.038) compared with the unadjusted model (R^2^Y = 50.8%, Q^2^ = 40.7%, F = 5.9, *p* = 0.011), indicating that the steroid-based discrimination was largely independent of baseline positive-symptom severity. For the olanzapine–risperidone contrast, adjustment for baseline PANSS-P attenuated the model below significance (R^2^Y = 35.8%, Q^2^ = 26.7%, F = 2.9, *p* = 0.084) relative to the unadjusted model (R^2^Y = 62.8%, Q^2^ = 56.2%, F = 5.1, *p* = 0.037), suggesting that positive-symptom severity contributed substantially to this discrimination. PANSS-P change (V1–V2) was not retained as a relevant predictor in any model.

The clozapine–olanzapine steroid discrimination remained significant after individual adjustment for cumulative CPZeq exposure, GAF, and treatment duration (all *p* ≤ 0.034), in addition to baseline PANSS-P, indicating that this contrast was not attributable to any single clinical or exposure covariate. The model adjusted for cumulative CPZeq exposure yielded a markedly higher apparent performance (R^2^Y = 95.9%, Q^2^ = 82.0%), which most likely reflects overfitting driven by the strong between-group difference in CPZeq (Supplementary Table S4A) rather than genuinely improved steroid-based discrimination, and is therefore reported for completeness only.

The clozapine–risperidone discrimination was likewise robust, remaining significant after individual adjustment for cumulative CPZeq exposure, GAF, and treatment duration (all *p* ≤ 0.046), with essentially unchanged model performance (R^2^Y ≈ 62–63%, Q^2^ ≈ 54–56%). In contrast, the olanzapine–risperidone discrimination did not reach statistical significance in any covariate model (R^2^Y = 34.2%, Q^2^ = 25.3%, F = 2.7, *p* = 0.097); notably, no valid predictive component could be extracted after adjustment for GAF, indicating the absence of a robust steroid-based separation between these two groups independent of clinical severity.

Because the inclusion of covariates did not materially alter the steroid contributions to the predictive components, and the steroid-level results remained nearly identical to those obtained in the unadjusted models, subsequent interpretations are based on the models without covariates.

### 2.3. Distinctive Effects of Clozapine

Clozapine produced a distinctive and internally coherent steroidomic signature that differentiated it from both olanzapine and risperidone. The pattern was characterized by coordinated changes across multiple steroidogenic pathways, suggesting a drug-specific modulation of neurosteroidogenesis. Compared with both comparator treatments, clozapine was associated with a selective reduction in early Δ^5^ steroids, most prominently DHEA and 7-oxo-DHEA, alongside broad elevations in downstream 5α-reduced pregnane neuroactive steroids. These changes indicate a shift away from Δ5 androgen precursors toward enhanced 5α-reductive metabolism. Age-adjusted ANCOVA further confirmed that these differences were not attributable to group disparities in age or BMI (Supplementary Table S2).

The OPLS and OMR analyses, supported by age-adjusted ANCOVA, consistently identified:

- Decreased DHEA and 7-oxo-DHEA, reflecting reduced flux through the Δ^5^ steroid pathway (Table 3 and Table 7, Supplementary Table S1; also supported by Δ^5^-to-precursor ratios in Table S4A and by absolute DHEA level differences in Supplementary Table S2);
- Marked increases in 5α-reduced pregnane neuroactive steroids, including allopregnanolone derivatives and conjugated 5α-pregnane diols/triols (Table 3 and Table 7, Supplementary Table S1; further supported by SRD5A-related ratios in Table S4F and by higher levels of 5α-pregnane-3α,20α-diol and its conjugates in Supplementary Table S2);
- Increased downstream androgen metabolites, such as 5β-androstane-3α,17β-diol (Table 7, Supplementary Table S2; consistent with AKR1D1-related ratios in Table S4G and with elevated 5α/5β-reduced androstanes in Supplementary Table S2);
- Reduced *CYP17A1* lyase proxy activity, indicating attenuated conversion of 17-hydroxypregnenolone/progesterone toward androgen precursors (Table 12; supported by lyase-related ratios in Table S4C,J, and by lower A/17-OH-P and A/17-OH-20α-DHP ratios in Supplementary Table S2);
- Increased SRD5A1/2 proxy activity, consistent with enhanced 5α-reduction in both pregnane and androgen substrates (Table 12; further supported by SRD5A-dependent ratios in Table S4F and by higher 5α-reduced pregnane neuroactive steroids and androstanes (e.g., 3α,5α-THA and 3α,5α-17-PD) in Supplementary Table S2);
- Increased cortisol-to-cortisone ratio, suggesting higher HSD11B1 proxy activity or reduced HSD11B2-mediated inactivation of cortisol (Supplementary Table S2; supported by HSD11B1/HSD11B2-related ratios in Table S4D and by lower cortisone and higher 11β-hydroxyepiandrosterone/11β-hydroxyandrosterone conjugates in Supplementary Table S2).

Taken together, these pathway-level shifts point toward a clozapine-specific enhancement of GABAergic neurosteroidogenesis, accompanied by reduced androgenic Δ5 precursor availability and altered glucocorticoid metabolism (higher F/E ratio). This coordinated pattern may contribute to clozapine’s unique neurobiological profile, including its effects on excitatory–inhibitory balance, stress responsivity, and inflammation.

### 2.4. Comparison with Olanzapine

Olanzapine exhibited a steroidomic profile that differed from clozapine both in direction and pathway-level coherence. While clozapine was characterized by a coordinated shift toward enhanced 5α-reductive metabolism and reduced Δ5 precursor availability, olanzapine showed a pattern dominated by higher DHEA concentrations and elevations in several downstream androgen-related metabolites (Supplementary Table S2). These differences were reflected consistently across multivariate models: clozapine demonstrated lower DHEA and 7-oxo-DHEA (Table 3, Supplementary Table S1), while olanzapine showed higher DHEA and 7α/7β-hydroxy-DHEA (Supplementary Table S2), indicating divergent modulation of the Δ5 steroid pathway.

At the pathway level, clozapine displayed broad and internally coherent increases in 5α-reduced pregnane neuroactive steroids, including allopregnanolone derivatives and multiple 5α-pregnane triols (Table 3 and Table 7, Supplementary Table S1). In contrast, olanzapine showed more selective increases in androgenic- and Δ5-related metabolites, with limited elevation of 5α-reduced pregnanes. These distinctions were further supported by enzymatic ratio analyses: clozapine showed reduced CYP17A1 lyase proxy activity (Table 12; Supplementary Table S4C,J) and increased SRD5A1/2 proxy activity (Table 12; Supplementary Table S4F), whereas olanzapine did not exhibit a comparably coherent pattern across these enzymatic domains.

Despite these pathway-level differences, global trend tests for total and unconjugated steroids did not significantly distinguish clozapine from olanzapine (*p* = 0.515 and *p* = 0.409), indicating substantial overlap in overall steroid concentrations. The observed distinctions therefore arise primarily from multivariate coordination and enzymatic pathway structure, rather than from large univariate differences. Accordingly, these findings should be interpreted as descriptive and hypothesis-generating, reflecting potential mechanistic divergence rather than definitive evidence of drug-specific steroidogenic modulation.

Taken together, olanzapine’s profile appears more closely aligned with metabolic activation and androgenic pathway engagement, whereas clozapine demonstrates a more integrated shift toward 5α-reductive neurosteroidogenesis. These qualitative differences may contribute to their distinct neurobiological and clinical profiles, although confirmation in larger and longitudinal samples is required.

## 3. Discussion

The coordinated steroidomic patterns identified in this study indicate that antipsychotics modulate neuroendocrine pathways more broadly and systematically than previously recognized [23,24,29]. Rather than isolated changes in individual metabolites, the results reveal pathway-integrated shifts spanning Δ5-androgen precursors, 5α-reductive metabolism, glucocorticoid activation, and downstream androgen turnover. Among the three medications examined, clozapine (Clo) exhibited the most distinctive and internally coherent steroidomic signature, whereas olanzapine (Ola) and risperidone (Ris) showed more limited or less coordinated patterns.

Although global trend tests did not detect significant differences between Clo and Ola in total or unconjugated steroid levels (*p* = 0.515 and *p* = 0.409), multivariate OPLS/OMR analyses consistently revealed a structured, directional pattern of pathway-level changes that distinguished Clo from both comparators. This highlights an important conceptual point: multivariate coordination can reveal biologically meaningful patterns even when univariate differences are modest, underscoring the value of steroidomic approaches in psychopharmacology.

### 3.1. Mechanistic Interpretation: Substrate Flux and Enzymatic Pathway Shifts

Mechanistically, Clo was associated with selective reductions in early Δ5 steroids, most prominently DHEA and 7-oxo-DHEA (Table 3 and Table 7, Supplementary Table S1), supported by Δ5-to-precursor ratios indicating reduced CYP17A1 flux (Supplementary Table S4A). This reduction in Δ5 precursors coincided with broad elevations in 5α-reduced pregnane neuroactive steroids (NASs), including allopregnanolone, isopregnanolone, and multiple conjugated 5α-pregnane triols (Table 3 and Table 7, Supplementary Table S1). The pattern was further reinforced by SRD5A-dependent ratios (Supplementary Table S4F), indicating enhanced 5α-reduction in both pregnane and androgen substrates [16,17].

Taken together, these findings suggest a re-routing of the precursor flux away from Δ5-androgen pathways toward 5α-reduced GABAergic NAS [16,17]. This interpretation is supported by reduced CYP17A1 lyase proxy activity (Table 12; Supplementary Table S4C,J), reflected in lower A/17-OH-precursor ratios and attenuated conversion of 17-hydroxypregnenolone/progesterone toward androgenic pathways. Despite this reduction, downstream androgen metabolites such as 5β-androstane-3α,17β-diol were elevated (Table 7, Supplementary Table S2), consistent with increased AKR1D1-related flux (Supplementary Table S4G). This suggests that androgen metabolism may be diverted toward 5α/5β-reduced derivatives rather than classical Δ5 androgen precursors.

Glucocorticoid metabolism also showed coordinated modulation. Clo was associated with an increased cortisol-to-cortisone ratio (Supplementary Table S2), supported by HSD11B1/HSD11B2-related ratios (Supplementary Table S4D), indicating enhanced glucocorticoid activation or reduced inactivation. This aligns with evidence that Clo modulates stress-responsive pathways, reduces oxidative stress, improves mitochondrial function, and enhances synaptic plasticity—mechanisms increasingly recognized as contributors to its unique efficacy in treatment-resistant schizophrenia (TRS) [11,12,13,14].

### 3.2. Functional Implications: Excitatory–Inhibitory Balance and Stress Responsivity

The observed increases in 5α-reduced pregnane NAS have potential functional implications. Allopregnanolone is a potent positive allosteric modulator of GABAAR, whereas its 3β-epimer isopregnanolone antagonizes this effect, and sulfated derivatives generally act as negative modulators. Clo-associated increases therefore do not imply a uniformly inhibitory action but rather a shift in the balance of opposing NAS, compatible with an enhanced inhibitory tone in the excitatory–inhibitory (E/I) balance [16,17]. This interpretation remains cautious: peripheral steroid levels do not directly index central GABAergic signaling, and DHEA/DHEAS exert complex, concentration-dependent effects that cannot be reduced to simple excitatory roles. Nevertheless, the coordinated pattern aligns with neurobiological models of TRS emphasizing impaired GABAergic signaling, disrupted neuroplasticity, and altered stress responsivity [16,17].

Changes in androgen turnover and glucocorticoid activation further support the notion that Clo engages multiple neurobiological domains simultaneously. Reduced CYP17A1 lyase proxy activity and increased SRD5A1/2 proxy activity suggest a systematic re-routing of steroid metabolism toward GABAergic pregnane and androstane derivatives. In parallel, enhanced glucocorticoid activation may reflect modulation of stress pathways, consistent with Clo’s known effects on oxidative stress, mitochondrial function, and synaptic plasticity.

### 3.3. Comparison with Olanzapine and Risperidone

Ola exhibited a steroidomic profile more closely aligned with its known metabolic effects. Higher DHEA- and androgen-related metabolites (Supplementary Table S2) suggest increased Δ5-androgenic flux, potentially reflecting metabolic activation associated with weight gain and insulin resistance [23,24]. Although Ola increased several steroid concentrations, its pathway-level effects lacked the coordinated 5α-reductive pattern observed for Clo. Ris showed the least extensive steroidogenic modulation, consistent with its more selective receptor profile and the symptomatic improvements observed in this group.

### 3.4. Interpretive Constraints and Methodological Considerations

Several interpretive constraints warrant emphasis.

- First, although the steroidomic patterns were robust to adjustment for symptomatic severity, functioning, and cumulative dose, residual associations with positive-symptom severity indicate that symptom dimensions cannot be fully disentangled in this modest sample.
- Second, the between-group age difference corresponded to a large effect size (η^2^ = 0.216), driven mainly by the Clo–Ris contrast, and age-dependent steroidogenesis may have contributed to some observed patterns despite covariate adjustment.
- Third, the between-group differences were generally of small-to-moderate magnitude, and the interpretive value of the findings lies primarily in the coordinated multivariate pattern, not in any single metabolite.
- Fourth, peripheral steroid levels do not directly reflect central neurosteroidogenesis, and mechanistic interpretations must remain provisional until validated by central physiological measures.

### 3.5. Conceptual Significance

Despite these limitations, the present results suggest that Clo may be associated with a mechanistically meaningful modulation of neurosteroidogenesis, characterized by reduced Δ^5^-precursor availability, enhanced 5α-reductive metabolism, altered androgen turnover proxies (e.g., A/20α-DHP, 11β-OH-A/B), and increased glucocorticoid activation. These pathway-level shifts may contribute to Clo’s distinctive neurobiological profile, including potential restoration of inhibitory tone, modulation of stress pathways, and attenuation of inflammatory processes [3,4,5,6,7,8,9,10,11,12,13,14,15]. By integrating concentration changes, enzymatic ratios, and pathway-level trends, this study identifies neuroactive steroids and their enzymatic pathways as candidate contributors to treatment response, resistance, and Clo’s unique therapeutic profile.

These mechanistic insights provide a foundation for the subsequent sections on clinical implications, future directions, and biomarker development, and they highlight neurosteroidogenesis as a promising domain for translational research in psychopharmacology.

## 4. Potential Clinical Implications of the Findings

The present findings suggest that antipsychotic-induced modulation of steroidogenesis may have clinically relevant consequences for symptom domains, treatment response, and side-effect profiles. Clozapine’s coordinated steroidomic signature—characterized by reduced Δ5-precursor availability, enhanced 5α-reductive metabolism, and altered glucocorticoid activation—may reflect biological processes relevant to its unique efficacy in treatment-resistant schizophrenia (TRS). Increased 5α-reduced pregnane neuroactive steroids (NASs), including allopregnanolone derivatives, could contribute to modulation of GABAergic tone, stress responsivity, and neuroplasticity, domains implicated in TRS pathophysiology [16,17].

Alterations in androgen turnover and glucocorticoid activation may further influence affective regulation, cognitive processes, and inflammatory pathways. Reduced CYP17A1 lyase proxy activity suggests attenuated androgenic flux, whereas increased SRD5A1/2 proxy activity may enhance production of GABAergic pregnane and androstane derivatives. These shifts may interact with known clozapine-associated improvements in oxidative stress, mitochondrial function, and synaptic plasticity, potentially contributing to its clinical profile beyond dopamine and serotonin receptor modulation [25,26,30].

In contrast, olanzapine’s elevation of DHEA and androgen-related metabolites aligns with its metabolic side-effect profile, including weight gain and insulin resistance [23,24]. Risperidone’s comparatively limited steroidogenic modulation is consistent with its more selective receptor profile and may partly explain its different clinical and metabolic characteristics.

Although these interpretations remain provisional, they highlight neurosteroidogenesis as a potential mechanistic domain influencing antipsychotic response, tolerability, and symptom trajectories. If validated in larger and longitudinal cohorts, steroidomic markers or enzymatic ratios could contribute to individualized treatment strategies, early identification of TRS, or monitoring of treatment-related metabolic changes.

## 5. Future Directions

Future research should aim to validate and extend these findings using larger, longitudinal, and mechanistically oriented designs. Several avenues appear particularly promising:

i. **Longitudinal and dose-response studies.** Tracking steroidomic changes across treatment initiation, stabilization, and dose adjustments could clarify temporal dynamics, causal relationships, and potential early biomarkers of treatment response or resistance.
ii. **Integration with central physiological measures.** Peripheral steroid levels do not directly index central neurosteroidogenesis. Combining steroidomics with EEG-based E/I balance measures, neuroimaging of GABAergic circuits, or cerebrospinal fluid sampling could establish mechanistic links between peripheral signatures and central neurobiology [16,17].
iii. **Mechanistic studies of enzymatic pathways.** Experimental work examining CYP17A1, SRD5A1/2, HSD11B1/2, and AKR1D1 activity in cellular or animal models could clarify how antipsychotics influence steroidogenic flux and whether these effects are receptor-mediated, metabolic, or stress-responsive [11,12,14].
iv. **Clinical stratification and biomarker development.** Steroidomic profiles may help identify patients with TRS, predict clozapine response, or differentiate metabolic risk profiles associated with olanzapine. Enzymatic ratios could serve as candidate biomarkers for treatment personalization.
v. **Interaction with inflammation, stress, and metabolic pathways.** Given the interplay between steroidogenesis, HPA axis function, inflammation, and metabolic regulation, future studies should examine how these domains converge in antipsychotic treatment and whether steroidomic modulation contributes to metabolic or inflammatory side effects.
vi. **Translational and therapeutic innovation.** If neurosteroidogenic modulation proves mechanistically relevant, adjunctive strategies targeting SRD5A, HSD11B1/2, or NAS biosynthesis could be explored as potential enhancers of antipsychotic efficacy or modulators of side-effect burden.

Together, these directions could help establish neurosteroidogenesis as a meaningful mechanistic and clinical domain in psychopharmacology.

## 6. Study Strengths and Limitations

This study has several methodological strengths that enhance the interpretability of its findings. A key advantage lies in the pathway-integrated analytical approach, which combined absolute concentrations, conjugated and unconjugated fractions, enzymatic ratios, and multivariate OPLS/OMR modeling. This design enabled the detection of coordinated steroidogenic patterns that would not have been apparent from univariate comparisons alone. The inclusion of mechanistic proxies for CYP17A1, SRD5A1/2, HSD11B1/HSD11B2, and AKR1D1 provided additional resolution into substrate flux and enzymatic pathway activity, allowing the identification of drug-specific signatures at the level of steroidogenic networks rather than isolated metabolites. Importantly, the robustness of the clozapine-associated patterns to adjustment for symptomatic severity, functioning, treatment duration, and cumulative dose argues against these findings being merely secondary to illness severity or exposure, and supports a drug-related contribution. The comparative design, involving three mechanistically distinct antipsychotics, further strengthened the ability to identify drug-specific steroidomic profiles [29]. Steroid quantification was performed using GC-MS/MS, a gold-standard analytical platform that provides high specificity and sensitivity across a broad panel of endogenous steroids, including resolution of isomeric and isobaric species, thereby reducing the risk of misidentification or co-elution artifacts that may affect immunoassay-based measurements [31,32,33].

Several limitations must also be acknowledged. The modest sample size limits statistical power, increases uncertainty in effect estimates, and restricts generalizability. The cross-sectional nature of the comparisons precludes causal inference and does not allow assessment of temporal dynamics or dose-response relationships. Although age was included as a covariate, the between-group age difference—particularly the Clo–Ris contrast—was associated with a large effect size (ANOVA *p* = 0.069; ηp^2^ = 0.216; Hedges’ g = −1.28, 95% CI −2.51 to −0.04; Table 13), and given the age dependence of steroidogenesis, residual influence cannot be fully excluded. Peripheral plasma measurements provide only an indirect index of central neurosteroidogenesis, and mechanistic interpretations must therefore remain cautious [16,17]. Because concomitant medications were not documented systematically across all participants, some degree of residual clinical confounding related to polypharmacy cannot be fully excluded.

Finally, the between-group differences in individual metabolites were generally small to moderate in magnitude; the interpretive value of the findings lies primarily in the coordinated multivariate patterns rather than in any single effect. Taken together, these strengths and limitations indicate that the present results should be viewed as hypothesis-generating but mechanistically informative, providing a foundation for future longitudinal and translational studies aimed at clarifying the role of neurosteroidogenesis in antipsychotic response and treatment-resistant schizophrenia.

## 7. Materials and Methods

### 7.1. Study Design and Participants

This study employed a prospective, observational cohort design without randomization or blinding, reflecting real-world clinical decision-making in early psychosis, where patients initially presenting with FEP may subsequently develop treatment-resistant schizophrenia (TRS). Male patients presenting with FEP were enrolled at baseline (V1) and reevaluated after follow-up (V2). Treatment allocation was determined by treating psychiatrists based on clinical indication rather than experimental assignment.

The clozapine (Clo) group consisted of patients who met clinical criteria for treatment-resistant schizophrenia (TRS), defined as an insufficient response to at least two adequate antipsychotic trials, persistent positive symptoms, and functional impairment. Switching procedures, prior antipsychotic failures, and diagnostic criteria at enrollment were verified from clinical records. Clo was initiated according to national guidelines for TRS.

Information on concomitant medications was available only partially from clinical records and was not collected systematically across all participants. Because these data were incomplete and not suitable for quantitative or group-level comparison, concomitant medications are not reported. Benzodiazepines and metabolic medications were not prescribed in this cohort. Treatment-switching procedures and the clinical context of referral to the NIMH are described to clarify the naturalistic polypharmacy patterns typical for this patient population.

The olanzapine (Ola) and risperidone (Ris) groups included patients treated with these agents as first-line or early-phase antipsychotics. All participants were male (Clo *n* = 6; Ola *n* = 14; Ris *n* = 5). Demographic and clinical characteristics—including duration of untreated psychosis (DUP), duration of illness (DI), treatment duration, cumulative chlorpromazine equivalents, PANSS scores, and metabolic parameters—are summarized in Table 1.

The absence of a healthy control group reflects the study’s focus on comparative pharmacodynamic effects among antipsychotics rather than case–control differences. All participants provided written informed consent. The study was approved by the NIMH CZ Ethics Board (protocol 127/17).

### 7.2. Steroid Quantification

Serum concentrations of 85 steroids (Table 2) were quantified using the validated GC–MS/MS method [34]. GC–MS/MS provided high analytical specificity and sensitivity, minimizing risks of misidentification or co-elution artifacts [31,32,33].

#### 7.2.1. Sample Collection, Processing, and Storage

Peripheral venous blood was collected from each participant between 08:00 and 09:00 a.m. to minimize the influence of circadian variation, particularly on glucocorticoid levels. Samples were allowed to clot at room temperature, and serum was separated by centrifugation (5 min at 2000× *g* at 2 °C). A 1 mL serum aliquot was used for steroid analysis; serum aliquots were stored at −20 °C until analysis. Repeated freeze–thaw cycles were avoided; stability tests confirmed no significant differences after three freeze–thaw cycles or after 24 h at room temperature (Section 7.2.5).

#### 7.2.2. Chemicals and Internal Standards

Most steroid standards, including the deuterated internal standards (ISs), were purchased from Steraloids (Newport, RI, USA); D7-cortisone ([2,2,4,6,6,12,12-D7]-cortisone) was purchased from Sigma-Aldrich (St. Louis, MO, USA). Sylon BTZ (Supelco/Merck, Bellefonte, PA, USA), methoxyamine hydrochloride (St. Louis, MO, USA), and all other solvents and reagents were obtained from Merck (Darmstadt, Germany). All solvents were of HPLC grade. Stock solutions of external and internal standards were prepared in methanol at 1 mg/mL (D7-cortisone at 10 µg/mL).

The full multi-matrix method on which the present analysis is based originally employed a larger panel of deuterated internal standards. For the present study, restricted to male serum, the IS set was reduced to the three standards that remained isotopically stable under the strongly acidic conditions of methanolysis:

D6-DHEA ([2,2,3,4,4,6-D6]-DHEA; Steraloids, Newport, RI, USA)—IS1

D4-cortisol ([9,11,12,12-D4]-cortisol; Steraloids, Newport, RI, USA)—IS2

D7-cortisone ([2,2,4,6,6,12,12-D7]-cortisone; Sigma-Aldrich, St. Louis, MO, USA)—IS3

The remaining deuterated standards used in the original method (e.g., D9-progesterone and D8-17-hydroxyprogesterone) were omitted because they proved isotopically unstable during methanolysis, undergoing deuterium–hydrogen exchange via acid-catalyzed keto–enol tautomerism. The mixed IS working solution was prepared from the individual stock solutions as follows: 10 µL D6-DHEA (1 mg/mL), 100 µL D4-cortisol (1 mg/mL), and 50 µL D7-cortisone (10 µg/mL). A fixed volume of 15 µL of this mixed IS solution was added to every calibration, zero, quality-control, and study sample prior to extraction.

D6-DHEA was used as the internal standard for unconjugated Δ^5^ and Δ^4^ steroids, androstane derivatives, pregnenes, and pregnanes, as well as for all conjugated steroids; D4-cortisol and D7-cortisone were used for glucocorticoids and related 11-oxygenated steroids. Because all study samples were from men, only the male serum matrix was used for calibration and validation; the other matrices reported in the original method (follicular, luteal, pregnancy, and umbilical cord serum) were not relevant and were omitted.

#### 7.2.3. Sample Preparation

Unconjugated and conjugated steroids were determined from the same 1 mL serum aliquot in two consecutive fractions.

**Unconjugated steroids.** After addition of the mixed IS solution (15 µL), unconjugated steroids were extracted from 1 mL of serum with diethyl ether (3 mL). The ether extract was evaporated in a block heater (Grant Instruments Ltd., OBD4; Shepreth, Cambridgeshire, UK) at 37 °C. Lipids were removed by partitioning the dry residue between methanol–water 4:1 (1 mL) and pentane (1 mL); the pentane phase was discarded, and the polar phase was dried in a vacuum centrifuge (Thermo Scientific, Waltham, MA, USA) equipped with a freeze-dryer unit (Jouan S.A., Saint-Herblain, France) at 60 °C (2 h). The residue was dissolved in acetonitrile (100 µL), transferred to a conical vial, and dried under nitrogen using a custom-built evaporator (Sipoch s.r.o., Praha, Czech Republic). Derivatization was performed in two steps: (i) oximation with 2% methoxyamine hydrochloride (Sigma-Aldrich (Merck), St. Louis, MO, USA) in pyridine (60 °C, 1 h) to form methyloxime (MOX) derivatives, and (ii) after drying under nitrogen, silylation with Sylon BTZ [BSA + TMCS + TMSI, 3:2:3] (Supelco/Merck, Bellefonte, PA, USA) (90 °C, 24 h) to form TMS–MOX derivatives. After drying under nitrogen and addition of ~1 mg ammonium bicarbonate (Sigma-Aldrich, St. Louis, MO, USA), the residue was partitioned between isooctane (Sigma-Aldrich, St. Louis, MO, USA) (100 µL) and N,N-dimethylformamide (Sigma-Aldrich, St. Louis, MO, USA) (50 µL); the mixture was vortexed (1 min) and centrifuged (20 min, 3000 rpm). The lower polar layer was discarded and 2 µL of the upper non-polar layer was injected into the GC–MS/MS system.

**Conjugated steroids.** Steroid conjugates remaining in the polar residue after diethyl-ether extraction were processed by adding the mixed IS solution (15 µL) followed by methanol (1 mL, 1 min mixing). After centrifugation (20 min, 3000 rpm) the supernatant was transferred to a clean tube and dried (vacuum centrifuge (Thermo Scientific, Waltham, MA, USA), 37 °C, 5 h). Conjugates were cleaved by acid methanolysis according to Dehennin: 1 mL of 1 M TMCS (Sigma-Aldrich, St. Louis, MO, USA) in methanol (Merck, Darmstadt, Germany) was added, and hydrolysis proceeded for 1 h at 55 °C. After neutralization with 100 mg sodium bicarbonate and drying (37 °C, 5 h), the residue was reconstituted in 500 µL chromatographic water and then processed identically to the unconjugated fraction. This single step cleaves sulfates and glucuronides simultaneously; conjugated steroids are therefore reported as total polar conjugates (sulfates, disulfates, and glucuronides). For the conjugate fraction, only D6-DHEA served as an internal standard, as D4-cortisol and D7-cortisone are not stable during methanolysis; the suitability of unconjugated D6-DHEA as a surrogate is supported by the high methanolysis efficiency (98 ± 11%, Section 7.2.5).

#### 7.2.4. GC–MS/MS Instrumentation and Chromatographic Conditions

Analyses were performed on a **Shimadzu GCMS-TQ8050 NX** triple-quadrupole system (Shimadzu, Kyoto, Japan) equipped with an AOC-20s autosampler (Shimadzu, Kyoto, Japan) and operated in multiple-reaction monitoring (MRM) mode. Chromatographic separation was achieved on a **RESTEK Rtx-50** capillary column (medium polarity; 15 m × 0.25 mm i.d.; 0.1 µm film thickness) (Restek Corporation, Bellefonte, PA, USA). Electron-impact ionization was applied at **60 V** (emission current 151 µA). The injection port, ion source, and interface temperatures were **220 °C**, **300 °C**, and **310 °C**, respectively. Samples (2 µL) were injected in splitless, high-pressure mode (200 kPa for 1 min) with helium as the carrier gas at constant linear velocity (60 cm/s); septum purge flow was 3 mL/min and detector voltage was 2.2 kV.

The oven temperature program was as follows: 80 °C (1 min hold), increased at 40 °C/min to 190 °C, 6 °C/min to 210 °C, 20 °C/min to 300 °C, and 40 °C/min to 320 °C (4 min hold). The initial inlet pressure was 34 kPa, and the total run time was 16.08 min. Time-segmented MRM acquisition windows were used to maximize sensitivity. For two highly abundant steroid sulfates (DHEA sulfate and androsterone sulfate) that exceeded the upper limit of the linear dynamic range at a 2 µL injection volume, a reduced injection volume (0.1 µL) was used; the method was validated for these analytes at a low-volume injection. Collision energies for all MRM transitions were optimized using the Shimadzu MRM Optimization Tool (Shimadzu, Kyoto, Japan).

#### 7.2.5. Method Validation

The method was validated in the male serum matrix using charcoal-stripped serum for calibration. A 9-point logarithmic calibration curve was constructed by plotting the logarithm of the response factor (analyte area/IS area) against the logarithm of the external-standard concentration; responses were corrected for procedural losses using internal-standard yields. The acceptance criterion for each back-calculated calibration level was ≤15% deviation from the nominal value.

**Linearity**

Linearity was satisfactory across the full concentration range (Table S5).

**Limit of detection (LOD) and limit of quantification (LOQ)**

The lowest non-zero standard on the calibration curve defined the analytical sensitivity. The analyte response at the LOQ was required to be at least five times the analyte response of the zero calibrator, and the bias at the LOQ had to remain within ±20% of the nominal concentration, as determined from replicates prepared in six independent runs. Imprecision at the LOQ was required to be ≤20% RSD, based on six replicates analyzed across four runs, in accordance with the Bioanalytical Method Validation [35].

The LOD was considered sufficient in all cases where intra- and/or inter-assay precision did not exceed 15% RSD. LOQ performance was borderline for several low-abundance analytes in non-pregnant subjects (AD3α5β17β, AD3α5β17β, E1, PD3α5β17) and for E2 and THP3α5α in men, reflecting physiological concentration ranges rather than methodological limitations.

**Precision**

Precision (intra-assay, *n* = 6 within one batch) and intermediate precision (inter-assay, *n* = 6 across days) were expressed as RSD; analytes exceeding 15% RSD in the male matrix were excluded (Table S5).

**Recovery**

Recovery was evaluated using pooled male serum spiked at three physiologically relevant concentrations. Spiked and non-spiked samples were processed identically, and recovery was calculated as follows: Recovery (%) = 100 × [Measured concentration (spiked serum) − Measured concentration (charcoal-stripped serum)]/Added concentration.

**Accuracy**

Accuracy was expressed as % bias relative to the true spiked concentration at three levels in intra- and inter-day experiments; the ±15% bias criterion was met for all reported analytes (Table S5).

**Stability**

Stability was confirmed after three freeze–thaw cycles, 24 h at room temperature, 3-day post-derivatization storage, and 1-month stock-solution storage, with no significant differences.

**Methanolysis efficiency**

Methanolysis efficiency for representative steroid sulfates was 85–116% (98 ± 11%), confirming near-complete deconjugation and justifying the use of unconjugated D6-DHEA as a surrogate internal standard for the conjugate fraction.

**Specificity and selectivity**

Specificity/selectivity was verified in accordance with the U.S. FDA Guidance for Industry: Bioanalytical Method Validation [35]; interferences among co-eluting analytes were resolved by transition selection (e.g., 360 → 270 for epiandrosterone, 388 → 173 for isopregnanolone).

**Transfer of validation from GCMS-TQ8040 to GCMS-TQ8050 NX**

Analytical performance criteria (linearity, precision, accuracy, LOQ/LOD) were adopted from the original validation performed on the GCMS-TQ8040 platform (Shimadzu, Kyoto, Japan) because the extraction procedure, derivatization chemistry, and MRM transitions remained unchanged. GCMS-TQ8050 NX (Shimadzu, Kyoto, Japan) provides higher sensitivity and lower electronic noise, ensuring that all criteria satisfied on the TQ8040 are conservatively met or exceeded on the TQ8050 NX.

### 7.3. Metabolic Ratios as Proxies of Enzyme Activities

Enzymatic activities were inferred from precursor–product molar ratios, interpreted as proxy metabolic balances rather than as direct measures of tissue enzyme activity. To approximate the activity of individual steroidogenic enzymes, product-to-substrate concentration ratios were calculated, with a higher ratio reflecting greater conversion of substrate(s) to product(s). Ratios were evaluated for unconjugated steroids and, where indicated, for their conjugated (sulfated) forms (subscript C) and as conjugated-to-unconjugated ratios (subscript C/U). Because such ratios are also affected by adrenal and gonadal secretion, hepatic metabolism, binding proteins, renal clearance, circadian variation, and parallel enzymatic routes, they should be interpreted as proxy indicators of metabolic balance rather than as direct enzyme activities.

These ratios were grouped into enzyme-defined blocks (spanning SULT2A1/STS, CYP17A1 hydroxylase and lyase activities, HSD3B1/2, SRD5A1/2, AKR1D1, AKR1C1, AKR1C2, HSD17B3/AKR1C3, HSD11B1/2, CYP11B1, and CYP7B1/CYP3A4/CYP3A7), matching Supplementary Table S2. Full definitions of each ratio are provided in Table 6 and in Supplementary Table S2. The blocks were defined as follows:

- CYP17A1 (17,20-lyase, i.e., hydroxylase + lyase combined): Conversion of Δ^5^-pregnene and Δ^4^-pregnene precursors to the corresponding C19 androgens, e.g., DHEA/pregnenolone, androstenedione/progesterone, and the analogous 5α-/5β-reduced androstane-to-pregnane pairs (e.g., 3α,5α-THA/3α,5α-THP), plus 11β-OH-androstenedione/corticosterone.
- CYP17A1 (17α-hydroxylase only): 17-hydroxylation of pregnanes, e.g., 17-OH-pregnenolone/pregnenolone, 17-OH-progesterone/progesterone, 17-OH-20α-dihydroprogesterone/20α-dihydroprogesterone, and the corresponding 5α-/5β-reduced 17-hydroxy-pregnane pairs, plus cortisol/corticosterone (F/B).
- CYP17A1 (17,20-lyase only): Cleavage of the C17–C20 bond of 17-hydroxylated pregnanes to C19 androgens, e.g., DHEA/17-OH-pregnenolone, androstenedione/17-OH-progesterone, and the corresponding reduced-steroid pairs, plus 11β-OH-androstenedione/cortisol.
- HSD3B1/HSD3B2: Conversion of Δ5-3β-hydroxy steroids to Δ4-3-keto steroids, e.g., progesterone/pregnenolone, 17-OH-progesterone/17-OH-pregnenolone, 16α-OH-progesterone/16α-OH-pregnenolone, androstenedione/DHEA, and testosterone/androstenediol.
- SULT2A1 (vs. STS): Sulfation status expressed as the conjugated-to-unconjugated (C/U) ratio of each steroid (e.g., DHEA,C/U; pregnenolone, C/U), reflecting the net balance between steroid sulfotransferase (SULT2A1) and steroid sulfatase (STS).
- HSD11B1 (vs. HSD11B2) and CYP11B1: 11-oxoreduction/oxidation balance, e.g., cortisol/cortisone (F/E), 7-oxo-DHEA/7α-OH-DHEA, and 7β-/7α-OH-DHEA; CYP11B1 as 11β-hydroxylation, e.g., 11β-OH-androstenedione/androstenedione and the 11β-OH-tetrahydro-androstane/tetrahydro-androstane pairs.
- CYP7B1/CYP3A4/CYP3A7: 7α-/7β- and 16α-hydroxylation of Δ5 steroids, e.g., 7α-OH-DHEA/DHEA, 7β-OH-DHEA/DHEA, 16α-OH-pregnenolone/pregnenolone, and the corresponding androstene-triol/androstenediol pairs.
- SRD5A1/SRD5A2 (5α-reduction): 5α-reduction in Δ4-3-keto steroids, expressed as the sum of the 5α-reduced products over the Δ4 substrate. Progesterone: (5α-DHP + 3α/β,5α-THP)/progesterone, with the corresponding conjugated (C) and conjugated + unconjugated (C+U) variants. 20α-dihydroprogesterone: (5α,20α-THP + 3α/β,5α,20α-PD)/20α-DHP and its C and C+U variants. 17-OH-progesterone: 3α,5α,17-PD/17-OH-P and its C and C+U variants; 17-OH-20α-dihydroprogesterone: 3α/β,5α,17,20α-PT/17-OH-20α-DHP and its C and C+U variants. Androstenedione: (5α-DHA + 3α/β,5α-THA)/androstenedione and its C+U variant. Testosterone: (5α-DHT + 3α/β,5α,17β-AD)/testosterone and its C and C+U variants. 11β-hydroxy-androstenedione: 11β-OH-3α/β,5α-THA/11β-OH-A and its C and C+U variants.
- AKR1D1 (5β-reduction): 5β-reduction in Δ4-3-keto steroids, expressed as the sum of 5β-reduced products over the Δ4 substrate. Progesterone: 3α,5β-THP/progesterone and 3α/β,5β-THP,C (and C+U)/progesterone. 20α-dihydroprogesterone: 3α/β,5β,20α-PD/20α-DHP and (5β,20α-THP + 3α/β,5β-THP)/20α-DHP (C and C+U variants). 17-OH-progesterone: 3α,5β,17-PD/17-OH-P and its C and C+U variants; 17-OH-20α-dihydroprogesterone: 3α,5β,17,20α-PT/17-OH-20α-DHP and its C and C+U variants. Androstenedione: 3α,5β-THA/androstenedione and its C and C+U variants. Testosterone: 3α,5β,17β-AD/testosterone and its C and C+U variants. 11β-hydroxy-androstenedione: 11β-OH-3α,5β-THA/11β-OH-A and its C and C+U variants.
- AKR1C1 (vs. HSD17B2): 20α-oxidoreduction in pregnanes, e.g., 20α-dihydroprogesterone/progesterone, 20α-dihydropregnenolone/pregnenolone, and the corresponding 5α-/5β-reduced 20α-diol/20-keto pairs.
- AKR1C2 (vs. HSD17B2/HSD17B6): 3α/3β-oxidoreduction, expressed as 3α- over 3β-hydroxy epimer ratios, e.g., 3α,5α-THP/3β,5α-THP, 3α,5α-THA/3β,5α-THA, and the corresponding androstane and 11β-hydroxy-androstane epimer pairs, and 3α-hydroxy/3-keto and 3α,20α-diol/20-keto pairs reflecting 3α-HSD-type reduction.
- HSD17B3/AKR1C3 (vs. HSD17B2): 17β-oxidoreduction between C19 17-keto and 17β-hydroxy androgens, e.g., testosterone/androstenedione (T/A), androstenediol/DHEA, 5α-DHT/5α-androstanedione, and the corresponding 3α/β,5α/β-17β-hydroxy/17-keto androstane pairs.

A higher ratio indicates greater estimated activity of the corresponding enzyme (or the forward direction of a reversible reaction). Steroid and metabolite abbreviations are defined in Table 2. The complete list of ratios is provided in Supplementary Table S2.

### 7.4. Statistical Analysis

#### 7.4.1. Data Completeness and Visit Structure

All analyses used V2 serum steroid concentrations. V1 steroid values were not incorporated into the primary models because the baseline (V1) steroidomic dataset was incomplete. At the participant level, V1 steroid data were available for 5 of the 6 clozapine-treated participants analyzed at V2 (olanzapine, *n* = 14; risperidone, *n* = 5, with identical participant numbers at both visits). At the analyte level, missingness at V1 reached up to 40% for some steroids and was not proportionally distributed across treatment groups. Because this missingness was non-random and group-dependent, a longitudinal steroidomic model could not be reliably fitted, and imputation of V1 values was not considered appropriate. The V2 comparisons therefore represent cross-sectional pharmacodynamic profiles. Available baseline steroid values are provided in Supplementary Table S3 and may inform future longitudinal modeling.

#### 7.4.2. Covariates and Sensitivity Analyses

Age was included as a covariate in all univariate and multivariate analyses. Additional covariates (treatment duration, cumulative dose, and PANSS change) were explored in sensitivity analyses (Supplementary Table S1). Sensitivity analyses were performed for clinical covariates available for all participants. For PANSS and GAF, both baseline (V1) values and their change (V1–V2) were entered as covariates. For cumulative chlorpromazine-equivalent exposure (CPZeq), no baseline value was defined because all patients were drug-naive at V1; CPZeq was therefore modeled only as cumulative exposure at V2. Given the small per-group sample sizes (Clo, *n* = 6; Ris, *n* = 5), covariates were entered individually to avoid overfitting. The robustness of the clozapine–olanzapine steroid signature to adjustment for baseline positive-symptom severity supports a drug-related contribution, whereas attenuation of the olanzapine–risperidone signature suggests that part of this difference may reflect a baseline symptom dimension rather than treatment alone.

#### 7.4.3. Missing Data Handling

All analytes in the steroid panel were quantified with complete data across all participants; no values fell below the limit of detection, and no clinical records (PANSS, GAF, CPZeq, treatment duration) were missing. No imputation was therefore required, and all models were fitted on complete-case data.

#### 7.4.4. Variable Rescaling and Transformation

Prior to transformation, each steroid variable was rescaled using a fixed, deterministic procedure applied independently to every variable. Denoting the original values as x with a minimum $x_{min}$ and a median ${\tilde{x}}_{0.5}$ (both computed on the original, unshifted variable), the rescaled values were obtained as follows:

$$x^{\prime }=\;\frac{{x-x}_{min}+0.05\cdot {\tilde{x}}_{0.5}}{{\tilde{x}}_{0.5}}$$

Subtracting the minimum shifts the variable so that its lowest value is zero; adding the constant offset $0.05\cdot {\tilde{x}}_{0.5}$ ensures strictly positive values suitable for transformation; and dividing by the median brings all variables to a comparable, near-unit order of magnitude, avoiding numerical instability when transforming large values. Because this rescaling is strictly monotonic and linear, it preserves the relative structure of the data and yields statistically equivalent results to the unscaled data prior to transformation.

The rescaled variables were then subjected to a Box–Cox transformation, applied consistently across all univariate and multivariate analyses. For multivariate modeling (OPLS), the Box–Cox-transformed variables were additionally mean-centered and unit-variance scaled, ensuring that model results were independent of the original measurement scale. Residuals were inspected for normality and homoscedasticity following the preprocessing described in Section 7.4.

#### 7.4.5. Interpretation Framework

Given the pilot nature of the study, the small sample sizes, and the correlated structure of steroid networks, the interpretation of results follows a cautious framework. Univariate findings are considered exploratory, whereas multivariate analyses emphasize pattern-level discrimination rather than individual metabolite effects. Enzymatic ratios are treated as proxy indicators of metabolic balance and, for certain pathways, also approximate shifts in the activity of individual enzymes or isoforms acting unidirectionally (e.g., AKR1D1). Pathway-level trends are interpreted as coordinated but non-independent shifts within steroidogenic systems. Primary conclusions therefore focus on effect sizes, consistency across analytical approaches, and biological plausibility, while avoiding causal claims.

OPLS models were considered statistically validated only when the predictive component achieved significance in CV-ANOVA (*p* < 0.05); models not meeting this criterion were classified as exploratory and reported separately.

#### 7.4.6. Statistical Software

Statistical analyses were performed using SIMCA-P v.12.0 (Umetrics AB, Umeå, Sweden), STATGRAPHICS Centurion v.18.1.16, and NCSS 12 Statistical Software (2018). Additional data preprocessing and descriptive calculations were conducted in Microsoft Excel Office 2021.

## 8. Conclusions

This pilot study provides preliminary evidence that antipsychotic treatment is associated with coordinated, pathway-level differences in steroidogenesis in men who initially presented with first-episode psychosis. Across the three treatment groups, clozapine—administered exclusively to individuals meeting criteria for treatment-resistant schizophrenia (TRS)—displayed the most internally coherent steroidomic pattern, characterized by convergent trends across steroid classes, conjugation states, and enzymatic proxy ratios. These exploratory trends included higher levels of several 5α-reduced pregnane neuroactive steroids, lower concentrations of selected Δ5-androgenic and excitatory steroids, and shifts in glucocorticoid-related ratios. When interpreted cautiously, these findings may reflect pharmacodynamic features relevant to inhibitory tone, stress responsivity, and metabolic regulation. However, any mechanistic interpretation must remain tentative given the modest sample size, male-only design, cross-sectional comparisons, and the non-independence of steroidogenic pathways.

Olanzapine and risperidone showed more limited or metabolically oriented steroidomic differences, and global pathway-level trend tests did not reveal significant distinctions between clozapine and olanzapine. The apparent specificity of the clozapine profile therefore lies not in the magnitude of individual metabolite changes, but in the coordinated multivariate pattern spanning multiple steroidogenic pathways. Because clozapine treatment in this cohort is inseparable from the TRS status that emerged during follow-up, the observed associations cannot be attributed to clozapine alone. TRS-related neurobiology may interact with drug-specific pharmacological profiles, and this interaction could contribute to the steroidomic differences observed here. Disentangling drug effects from the treatment-resistant phenotype will require designs that include TRS patients treated with non-clozapine agents or clozapine-treated non-TRS comparators.

Taken together, these findings suggest that neurosteroidogenesis may represent a mechanistic domain contributing to interindividual variability in antipsychotic response, particularly in treatment-resistant presentations. Yet, given the exploratory nature of the study and the small sample size, no causal or definitive inferences can be drawn. Establishing whether specific neuroactive steroids or enzymatic proxy ratios may serve as potential biomarkers—or inform precision-oriented treatment strategies in schizophrenia—will require replication in larger, adequately powered, indication-matched, and longitudinal cohorts.

## Figures and Tables

**Table 1:** Baseline characteristics and paired post-treatment changes (V1–V2) in patients treated with clozapine, olanzapine, or risperidone.

Variable	Stage	Clozapine	Olanzapine	Risperidone	ANOVA
Age [years]	V1	22.2 (19.4, 25.4) †	25.2 (23.1, 27.6)	30.1 (25.9, 35.4)	F(2,22) = 2.4, *p* = 0.111, ηp2 = 0.181
	V2	23.7 (20.9, 26.9)	26.3 (24.2, 28.7)	31 (26.8, 36.4)	F(2,22) = 2.1, *p* = 0.147, ηp2 = 0.16
DUP [months]	V1	2.1 (0.706, 5.92)	1.01 (0.481, 2.05)	1.69 (0.499, 5.29)	F(2,22) = 0.4, *p* = 0.678, ηp2 = 0.0348
DI [months]	V1	11.4 (6.25, 23.1) *	3.56 (2.5, 5.09)	3.97 (2.19, 7.32)	F(2,22) = 3.2, *p* = 0.061, ηp2 = 0.225
V1–V2 [months]	V1	12.6 (11.6, 13.7)	13.7 (13, 14.5)	12.9 (11.8, 14)	F(2,21) = 1.1, *p* = 0.361, ηp2 = 0.0925
AP treatment [months]	V1	9.88 (6.76, 14.3) ***†††	1.41 (1.03, 1.91)	1.15 (0.708, 1.8)	F(2,18) = 20.5, *p* < 0.001, ηp2 = 0.695
	V2	20.8 (17.7, 26) *††	15.6 (14.5, 17)	13.7 (12.4, 15.4)	F(2,21) = 5.3, *p* = 0.014, ηp2 = 0.335
AP treatment [cCPZeq]	V1	328 (235, 431)	395 (332, 462)	331 (238, 435)	F(2,20) = 0.5, *p* = 0.607, ηp2 = 0.0488
	V2	545 (430, 672) **†	294 (233, 359)	266 (170, 375)	F(2,22) = 4.7, *p* = 0.02, ηp2 = 0.3
	Δ	230 (91.8, 371) **†	−101 (−183, −18.1)	−66.3 (−199, 69.4)	F(2,20) = 4.8, *p* = 0.02, ηp2 = 0.323
Education [years]	V1	12.5 (11.5, 13.8)	13.4 (12.6, 14.4)	13.5 (12.1, 15.4)	F(2,22) = 0.4, *p* = 0.659, ηp2 = 0.0372
	V2	12.8 (11.7, 14.2)	13.9 (12.9, 15)	13.7 (12.3, 15.7)	F(2,22) = 0.5, *p* = 0.646, ηp2 = 0.039
BMI [kg/m^2^]	V1	24.5 (22.5, 27.2)	25.6 (24.1, 27.5)	24.7 (22.5, 27.7)	F(2,22) = 0.2, *p* = 0.829, ηp2 = 0.0169
	V2	24.2 (22.2, 26.5)	27.9 (26.1, 29.9)	26.7 (24.1, 29.9)	F(2,22) = 1.7, *p* = 0.212, ηp2 = 0.131
	Δ	0.177 (−1.04, 1.7)	1.79 (0.83, 2.88) ∘	1.15 (−0.259, 2.91)	F(2,21) = 0.9, *p* = 0.423, ηp2 = 0.0786
PANSS positive [score]	V1	12.8 (10.5, 15.6)	10.1 (8.82, 11.5)	13.5 (10.8, 16.9)	F(2,22) = 2, *p* = 0.163, ηp2 = 0.152
	V2	10.9 (9.08, 13.8)	8.89 (8.07, 9.92)	8.46 (7.28, 10.1)	F(2,22) = 1.3, *p* = 0.298, ηp2 = 0.104
	Δ	−2.21 (−4.79, 0.858)	−1.38 (−3.22, 0.661)	−4.06 (−6.48, −1.05)	F(2,22) = 0.6, *p* = 0.537, ηp2 = 0.0549
PANSS negative [score]	V1	18 (14.5, 22.7)	13.9 (12.1, 16)	18.6 (14.7, 24.1)	F(2,22) = 1.7, *p* = 0.201, ηp2 = 0.136
	V2	17.8 (14.5, 21.2)	13.7 (11.5, 15.9)	15.2 (11.5, 18.9)	F(2,22) = 1.1, *p* = 0.34, ηp2 = 0.0935
	Δ	0.479 (−2.1, 3.33) †	−1.35 (−2.81, 0.206) ‡	−5.17 (−7.16, −2.94)	F(2,21) = 3.3, *p* = 0.058, ηp2 = 0.238
PANSS general [score]	V1	33.8 (28.9, 38.7)	27.9 (24.7, 31.1)	33.4 (28, 38.8)	F(2,22) = 1.5, *p* = 0.249, ηp2 = 0.119
	V2	27.7 (23.4, 33.5)	22.6 (20.4, 25.1)	22.2 (18.9, 26.6)	F(2,22) = 1.3, *p* = 0.295, ηp2 = 0.105
	Δ	−3.07 (−7.48, 3.7)	−6.11 (−8.34, −3.33)	−9.97 (−12.4, −6.62)	F(2,21) = 1.8, *p* = 0.195, ηp2 = 0.144
PANSS Σ [score]	V1	65.5 (55.8, 76.6)	52.1 (46.6, 58.1)	66.1 (55.4, 78.3)	F(2,22) = 2.3, *p* = 0.126, ηp2 = 0.171
	V2	59.3 (50, 69.9)	45.9 (40.8, 51.4)	46.5 (38.2, 56.1)	F(2,22) = 1.8, *p* = 0.191, ηp2 = 0.14
	Δ	−2.35 (−10.7, 8.61) †	−8.55 (−12.9, −3.5) ‡	−18.9 (−23.7, −13)	F(2,21) = 3.2, *p* = 0.061, ηp2 = 0.234
GAF [score]	V1	66.3 (57.4, 74)	71.6 (66.4, 76.3)	69.5 (60.3, 77.4)	F(2,22) = 0.4, *p* = 0.711, ηp2 = 0.0305
	V2	64 (52.1, 72.8) *	80.9 (76.5, 84.8)	79.5 (71.5, 86.1)	F(2,22) = 3.6, *p* = 0.045, ηp2 = 0.246
	Δ	−0.159 (−10.2, 9.08)	9.25 (3.28, 15)	12.2 (2.23, 21.5)	F(2,22) = 1.1, *p* = 0.36, ηp2 = 0.0887
Prolactin [mIU/L]	V1	198 (78.5, 398) *†	567 (399, 785)	916 (557, 1440)	F(2,18) = 3.8, *p* = 0.044, ηp2 = 0.294
	V2	92.8 (50.1, 160) *†	268 (190, 377)	347 (197, 612)	F(2,17) = 4, *p* = 0.037, ηp2 = 0.321
	Δ	−94.8 (−390, 165)	−283 (−484, −101) ∘	−488 (−868, −178)	F(2,15) = 1, *p* = 0.407, ηp2 = 0.113

Between-group differences (Clo, Ola, Ris) were tested by one-way ANOVA on Box–Cox-transformed data with LSD pairwise contrasts computed separately for each stage (V1, V2) and for the V1–V2 change (Δ); no stage factor was modeled. Estimates are retransformed least-squares means with non-symmetric 95% CIs (LSD). Within-group V1 → V2 changes were tested separately per drug using a two-sided paired randomization test (Monte Carlo, 100,000 simulations; NCSS v.12.0.18). Symbols—between-group LSD: * Clo vs. Ola, † Clo vs. Ris, ‡ Ola vs. Ris; within-group paired change: ∘. One/two/three symbols = *p* < 0.05/<0.01/<0.001. Absence of ∘ on a Δ row denotes a non-significant within-group change (*p* > 0.05). ηp^2^ is the ANOVA treatment-factor effect size (~0.01 small, ~0.06 medium, and >0.14 large). Abbreviations: V1, V2—visits 1, 2; BMI—body mass index; DI—duration of illness prior to V1; DUP—duration of untreated psychosis; AP treatment—antipsychotic treatment duration; cCPZeq—cumulative chlorpromazine equivalents (mg).

**Table 2:** Steroid abbreviations.

No.	Steroid	Abbreviation
1	Pregnenolone	Preg
2	Pregnenolone sulfate	PregC
3	20α-Dihydropregnenolone	20α-DHPreg
4	20α-Dihydropregnenolone sulfate	20α-DHPregC
5	17-Hydroxypregnenolone	17-OH-Preg
6	17-Hydroxypregnenolone sulfate	17-OH-PregC
7	16α-Hydroxypregnenolone	16α-OH-Preg
8	Dehydroepiandrosterone (DHEA)	DHEA
9	DHEA sulfate	DHEAC
10	7α-Hydroxy-DHEA	7α-OH-DHEA
11	7-oxo-DHEA	7-oxo-DHEA
12	7β-Hydroxy-DHEA	7β-OH-DHEA
13	Androstenediol	Adiol
14	Androstenediol sulfate	AdiolC
15	5-Androstene-3β,7α,17β-triol	3β,7α,17β-AT
16	5-Androstene-3β,7β,17β-triol	3β,7β,17β-AT
17	5-Androstene-3β,16α,17β-triol	3β,16α,17β-AT
18	5-Androstene-3β,16α,17β-triol sulfate	3β,16α,17β-ATC
19	Progesterone	P
20	20α-Dihydroprogesterone	20α-DHP
21	Conjugated 20α-dihydroprogesterone	20α-DHPC
22	17-Hydroxyprogesterone	17-OH-P
23	16α-Hydroxyprogesterone	16α-OH-P
24	17,20α-Dihydroxy-4-pregnene-3-one	17-OH-20α-DHP
25	Conjugated 17,20α-dihydroxy-4-pregnene-3-one	17-OH-20α-DHPC
26	Androstenedione	A
27	Testosterone	T
28	Conjugated testosterone	TC
29	5α-Dihydroprogesterone	5α-DHP
30	Allopregnanolone	3α,5α-THP
31	Allopregnanolone sulfate	3α,5α-THPC
32	Isopregnanolone	3β,5α-THP
33	Isopregnanolone sulfate	3β,5α-THPC
34	Pregnanolone	3α,5β-THP
35	Conjugated pregnanolone	3α,5β-THPC
36	Conjugated epipregnanolone	3β,5β-THPC
37	17-Hydroxyallopregnanolone	3α,5α,17-PD
38	17-Hydroxyallopregnanolone sulfate	3α,5α,17-PDC
39	17-Hydroxypregnanolone	3α,5β,17-PD
40	Conjugated 17-hydroxypregnanolone	3α,5β,17-PDC
41	5α,20α-Tetrahydroprogesterone	5α,20α-THP
42	Conjugated 5α,20α-tetrahydroprogesterone	5α,20α-THPC
43	5α-Pregnane-3α,20α-diol	3α,5α,20α-PD
44	Conjugated 5α-pregnane-3α,20α-diol	3α,5α,20α-PDC
45	5α-Pregnane-3β,20α-diol	3β,5α,20α-PD
46	Conjugated 5α-pregnane-3β,20α-diol	3β,5α,20α-PDC
47	Conjugated 5β,20α-tetrahydroprogesterone	5β,20α-THPC
48	5β-Pregnane-3α,20α-diol	3α,5β,20α-PD
49	Conjugated 5β-pregnane-3α,20α-diol	3α,5β,20α-PDC
50	5β-Pregnane-3β,20α-diol	3β,5β,20α-PD
51	Conjugated 5β-pregnane-3β,20α-diol	3β,5β,20α-PDC
52	5α-Pregnane-3α,17,20α-triol	3α,5α,17,20α-PT
53	Conjugated 5α-pregnane-3α,17,20α-triol	3α,5α,17,20α-PTC
54	5α-Pregnane-3β,17,20α-triol	3β,5α,17,20α-PT
55	Conjugated 5α-pregnane-3β,17,20α-triol	3β,5α,17,20α-PTC
56	5β-Pregnane-3α,17,20α-triol	3α,5β,17,20α-PT
57	Conjugated 5β-pregnane-3α,17,20α-triol	3α,5β,17,20α-PTC
58	5α-Androstane-3,17-dione	5α-DHA
59	Androsterone	3α,5α-THA
60	Androsterone sulfate	3α,5α-THAC
61	Epiandrosterone	3β,5α-THA
62	Epiandrosterone sulfate	3β,5α-THAC
63	Etiocholanolone	3α,5β-THA
64	Etiocholanolone sulfate	3α,5β-THAC
65	Epietiocholanolone sulfate	3β,5β-THAC
66	5α-Dihydrotestosterone	5α-DHT
67	Conjugated 5α-dihydrotestosterone	5α-DHTC
68	5α-Androstane-3α,17β-diol	3α,5α,17β-AD
69	Conjugated 5α-androstane-3α,17β-diol	3α,5α,17β-ADC
70	5α-Androstane-3β,17β-diol	3β,5α,17β-AD
71	Conjugated 5α-androstane-3β,17β-diol	3β,5α,17β-ADC
72	5β-Androstane-3α,17β-diol	3α,5β,17β-AD
73	Conjugated 5β-androstane-3α,17β-diol	3α,5β,17β-ADC
74	Conjugated 5β-androstane-3β,17β-diol	3β,5β,17β-ADC
75	Cortisol	F
76	Cortisone	E
77	Corticosterone	B
78	21-Deoxycortisol	DOF
79	11β-Hydroxyandrostenedione	11β-OH-A
80	11β-Hydroxyandrosterone	11β-OH-3α,5α-THA
81	11β-Hydroxyandrosterone sulfate	11β-OH-3α,5α-THAC
82	11β-Hydroxyepiandrosterone	11β-OH-3β,5α-THA
83	11β-Hydroxyepiandrosterone sulfate	11β-OH-3β,5α-THAC
84	11β-Hydroxyetiocholanolone	11β-OH-3α,5β-THA
85	11β-Hydroxyetiocholanolone sulfate	11β-OH-3α,5β-THAC

**Table 3:** Discrimination between clozapine- and olanzapine-treated patients based on steroid profiles evaluated by OPLS and OMR models.

		Orthogonal Predictions to Latent Structure (OPLS), Predictive Component							Ordinary Multiple Regression (OMR)				
	Variable	Variable Importance	t-Statistic		Component Loading	t-Statistic	R ^a^		Regression Coefficient		t-Statistic		
Explanatory variables (**X**)	DHEA	0.923	4.77	**	−0.197	−4.34	−0.492	**	−0.064		−2.54	*	↓
	7-oxo-DHEA	0.955	2.23	*	−0.278	−3.56	−0.640	**	−0.066		−2.15	*	↓
	17-OH-20α-DHPC	1.106	4.39	**	0.268	4.07	0.668	**	0.077		3.81	**	↑
	A	1.038	2.45	*	−0.123	−1.02	−0.310		−0.072		−1.84		↓
	5α-DHT	1.068	2.66	*	0.284	5.24	0.709	**	0.074		1.98	*	↑
	3α,5β-THPC	0.884	2.31	*	0.315	5.71	0.786	**	0.061		2.50	*	↑
	3α,5α,17-PD	0.811	2.13	*	0.222	4.81	0.554	**	0.056		2.21	*	↑
	3α,5α,17-PDC	0.966	3.19	**	0.292	3.51	0.728	**	0.067		2.90	*	↑
	3α,5β,17-PDC	0.601	3.89	**	0.244	3.84	0.610	**	0.042		3.90	**	↑
	3β,5α,17,20α-PTC	1.12	3.18	**	0.330	4.46	0.824	**	0.077		6.08	**	↑
	3α,5β,17,20α-PTC	1.135	4.98	**	0.285	4.60	0.710	**	0.079		9.59	**	↑
	3α,5β-THA	1.022	2.66	*	−0.146	−1.35	−0.364		−0.071		−1.89		↓
	3β,5α,17β-AD	1.023	2.07	*	0.290	5.27	0.724	**	0.071		1.76		↑
	3α,5β,17β-AD	1.438	3.63	**	0.257	3.16	0.642	**	0.099		2.60	*	↑
	F	0.628	2.55	*	−0.190	−2.98	−0.437	*	−0.043		−1.96	*	↓
	E	1.281	7.98	**	−0.292	−6.71	−0.671	**	−0.089		−3.61	**	↓
	11β-OH-A	0.573	2.14	*	−0.107	−0.76	−0.268		−0.040		−1.47		↓
**Y**	Clozapine (vs. olanzapine), LLR				1.000	7.89	0.713	**					
	R^2^Y = shared variability with explanatory variables (after CV = Q^2^), CV-ANOVA	50.8% (40.7%), F(2, 17) = 5.9, *p* = 0.011											
Number of relevant/total explanatory variables = 17/86; shared variability among explanatory variables (R^2^X) = 38.2%; predictive/orthogonal components = 1/0; sensitivity = 0.667(0.223–0.957); specificity = 1(0.768–1) (values with 95% CI).													

^a^ R = Component loadings expressed as correlation coefficients with predictive components; LLR = logarithm of likelihood ratio; * *p* < 0.05 and ** *p* < 0.01; CV = cross-validation; the *p*-value of CV-ANOVA (analysis of variance testing of cross-validated predictive residuals) indicates the probability level where a model with this F-value may be the result of just chance. Abbreviations: OPLS, orthogonal projections to latent structures; OMR, ordinary multiple regression; VIP, variable importance in projection; R^2^X, fraction of X-variance explained; R^2^Y, fraction of Y-variance explained; Q^2^, predictive ability estimated by cross-validation; CV-ANOVA, cross-validated ANOVA; LLR, logarithm of likelihood ratio. Steroid abbreviations are defined in Table 2. Arrows indicate direction of group differences: ↑ higher levels in the first group listed; ↓ lower levels in the first group listed.

**Table 4:** Discrimination between clozapine- and olanzapine-treated patients based on conjugated-to-unconjugated steroid ratios (C/U), a proxy for the balance between SULT2A1 and STS activities, evaluated by OPLS and OMR models.

		Orthogonal Predictions to Latent Structure (OPLS), Predictive Component							Ordinary Multiple Regression (OMR)			
	Variable	Variable Importance	t-Statistic		Component Loading	t-Statistic	R ^a^		Regression Coefficient	t-Statistic		
Explanatory variables (**X**)	DHEA, C/U	0.771	2.46	*	0.412	4.23	0.663	**	0.133	3.11	**	↑
	17-OH-20α-DHP, C/U	0.681	2.23	*	0.298	4.83	0.479	**	0.117	1.52		↑
	5α-DHT, C/U	0.815	2.68	*	−0.295	−6.49	−0.475	**	−0.140	−3.01	**	↓
	3α,5β-THP, C/U	1.19	2.00	*	0.341	1.61	0.548		0.205	1.75		↑
	3β,5α,17,20α-PT, C/U	0.798	2.10	*	0.327	2.08	0.526	*	0.137	2.27	*	↑
	3α,5β,17,20α-PT, C/U	1.165	3.19	**	0.432	2.49	0.695	*	0.201	2.54	*	↑
	3β,5α-THA, C/U	0.838	4.98	**	0.424	3.44	0.683	**	0.144	3.75	**	↑
	3α,5β,17β-AD, C/U	1.472	4.93	**	−0.369	−4.08	−0.595	**	−0.254	−3.21	**	↓
**Y**	Clozapine (vs. olanzapine), LLR				1.000	6.77	0.784	**				
	R^2^Y = shared variability with explanatory variables (after CV = Q^2^), CV-ANOVA	61.5% (48%), F(2, 17) = 7.8, *p* = 0.004										
Number of relevant/total explanatory variables = 8/33; shared variability among explanatory variables (R^2^X) = 34.7%; predictive/orthogonal components = 1/0; sensitivity = 0.667(0.223–0.957); specificity = 1(0.768–1) (values with 95% CI).												

^a^ R = Component loadings expressed as correlation coefficients with predictive components; LLR = logarithm of likelihood ratio; * *p* < 0.05, ** *p* < 0.01; CV = cross-validation; the *p*-value of CV-ANOVA (analysis of variance testing of cross-validated predictive residuals) indicates the probability level where a model with this F-value may be the result of just chance. Abbreviations: OPLS, orthogonal projections to latent structures; OMR, ordinary multiple regression; R^2^X, fraction of X-variance explained; R^2^Y, fraction of Y-variance explained; Q^2^, predictive ability estimated by cross-validation; CV-ANOVA, cross-validated ANOVA; LLR, logarithm of likelihood ratio; SULT2A1, steroid sulfotransferase; STS, steroid sulfatase. Steroid abbreviations are defined in Table 2. Arrows indicate direction of group differences: ↑ higher levels in the first group listed; ↓ lower levels in the first group listed.

**Table 5:** Discrimination between clozapine- and olanzapine-treated patients based on 3α/3β steroid molar ratios, a proxy for the balance between AKR1C2 and HSD17B2/HSD17B6 activities, evaluated by OPLS and OMR models.

		Orthogonal Predictions to Latent Structure (OPLS), Predictive Component							Ordinary Multiple Regression (OMR)			
	Variable	Variable Importance	t-Statistic		Component Loading	t-Statistic	R ^a^		Regression Coefficient	t-Statistic		
Explanatory variables (**X**)	3α,5α-THP/3β,5α-THP, C	0.578	2.17	*	0.128	0.85	0.194		0.107	2.18	*	↑
	3α,5α,17β-AD/3β,5α,17β-AD	1.099	3.00	**	−0.601	−5.13	−0.909	**	−0.203	−3.83	**	↓
	11β-OH-3α,5α-THA/11β-OH-3β,5α-THA	1.398	2.54	*	−0.507	−2.10	−0.767	*	−0.258	−1.74		↓
	3α,5α,17β-AD/5α-DHT	1.012	2.65	*	−0.593	−8.21	−0.897	**	−0.187	−3.51	**	↓
	3α,5α,17β-AD/5α-DHT, C	0.692	2.37	*	0.280	1.42	0.423		0.128	2.17	*	↑
**Y**	Clozapine (vs. olanzapine), LLR				1.000	2.24	0.626	*				
	R^2^Y = shared variability with explanatory variables (after CV = Q^2^), CV-ANOVA	39.2% (33.5%), F(2, 17) = 4.3, *p* = 0.031										
Number of relevant/total explanatory variables = 5/31; shared variability among explanatory variables (R^2^X) = 48.8%; predictive/orthogonal components = 1/0; sensitivity = 0.667(0.223–0.957); specificity = 1(0.768–1) (values with 95% CI).												

^a^ R = Component loadings expressed as correlation coefficients with predictive components; LLR = logarithm of likelihood ratio; * *p* < 0.05, ** *p* < 0.01; CV = cross-validation; the *p*-value of CV-ANOVA (analysis of variance testing of cross-validated predictive residuals) indicates the probability level where a model with this F-value may be the result of just chance. Abbreviations: OPLS, orthogonal projections to latent structures; OMR, ordinary multiple regression; R^2^X, fraction of X-variance explained; R^2^Y, fraction of Y-variance explained; Q^2^, predictive ability estimated by cross-validation; CV-ANOVA, cross-validated ANOVA; LLR, logarithm of likelihood ratio; AKR1C2, aldo-keto reductase family 1 member C2; HSD17B2, 17β-hydroxysteroid dehydrogenase type 2; HSD17B6, 17β-hydroxysteroid dehydrogenase type 6. Steroid abbreviations are defined in Table 2. Arrows indicate direction of group differences: ↑ higher levels in the first group listed; ↓ lower levels in the first group listed.

**Table 6:** Discrimination between clozapine- and olanzapine-treated patients based on steroid profiles, specifically 17β-steroid to 17-oxo ratios as a proxy for the balance between HSD17B3/AKR1C3 and HSD17B2 activities, evaluated by OPLS and OMR models.

		Orthogonal Predictions to Latent Structure (OPLS) Predictive Component							Ordinary Multiple Regression (OMR)			
	Variable	Variable Importance	t-Statistic		Component Loading	t-Statistic	R ^a^		Regression Coefficient	t-Statistic		
Explanatory variables (**X**)	Adiol/DHEA	0.949	4.58	**	0.322	4.09	0.542	**	0.045	0.63		↑
	3β,7α,17β-AT/7α-OH-DHEA	0.647	2.80	*	0.172	1.32	0.290		0.095	0.61		↑
	3β,7β,17β-AT/7β-OH-DHEA	0.651	2.31	*	0.256	1.91	0.431	*	−0.040	−0.33		↑
	T/A	1.052	5.38	**	0.362	4.57	0.610	**	0.105	1.07		↑
	T, C/A	0.933	2.38	*	0.305	1.83	0.514		0.094	0.61		↑
	T, C+U/A	1.077	3.79	**	0.372	2.93	0.625	*	0.096	0.85		↑
	3β,5α,17β-AD/3β,5α-THA	0.962	3.92	**	0.408	5.93	0.686	**	0.098	0.73		↑
	3α,5β,17β-AD/3α,5β-THA	1.481	3.72	**	0.531	3.30	0.894	**	0.624	7.96	**	↑
**Y**	Clozapine (vs. olanzapine), LLR				1.000	5.29	0.833	**				
	R^2^Y = shared variability with explanatory variables (after CV = Q^2^), CV-ANOVA	69.3% (57.6%), F(4, 15) = 5.1, *p* = 0.008										
Number of relevant/total explanatory variables = 8/18; shared variability among explanatory variables (R^2^X) = 64.5%; predictive/orthogonal components = 1/1; sensitivity = 0.833(0.359–0.996); specificity = 1(0.768–1) (values with 95% CI).												

^a^ R = Component loadings expressed as a correlation coefficient with predictive components; LLR = logarithm of likelihood ratio; * *p* < 0.05 and ** *p* < 0.01; CV = cross-validation; the *p*-value of CV-ANOVA (analysis of variance testing of cross-validated predictive residuals) indicates the probability level where a model with this F-value may be the result of just chance. Abbreviations: OPLS, orthogonal projections to latent structures; OMR, ordinary multiple regression; R^2^X, fraction of X-variance explained; R^2^Y, fraction of Y-variance explained; Q^2^, predictive ability estimated by cross-validation; CV-ANOVA, cross-validated ANOVA; LLR, logarithm of likelihood ratio. Steroid abbreviations are defined in Table 2. Arrows indicate direction of group differences: ↑ higher levels in the first group listed; ↓ lower levels in the first group listed.

**Table 7:** Discrimination between clozapine- and risperidone-treated patients based on steroid profiles evaluated by OPLS and OMR models.

		Orthogonal Predictions to Latent Structure (OPLS) Predictive Component							Ordinary Multiple Regression (OMR)			
	Variable	Variable Importance	t-Statistic		Component Loading	t-Statistic	R ^a^		Regression Coefficient	t-Statistic		
Explanatory variables (**X**)	Age	0.891	1.92	*	−0.132	−3.18	−0.616	**	−0.024	−1.71		↓
	Pregnenolone	0.836	2.36	*	0.152	3.31	0.711	**	0.023	2.41	*	↑
	20α-Dihydropregnenolone	1.011	7.15	**	0.202	20.30	0.944	**	0.027	7.40	**	↑
	17-Hydroxypregnenolone	0.859	2.36	*	0.137	2.86	0.641	*	0.023	2.13	*	↑
	16α-Hydroxypregnenolone	1.041	2.82	*	0.157	2.09	0.737	*	0.028	2.71	*	↑
	7α-Hydroxy-DHEA	0.844	1.92	*	0.140	1.87	0.655		0.023	1.94	*	↑
	7β-Hydroxy-DHEA	1.094	2.82	*	0.160	3.01	0.749	**	0.030	3.10	**	↑
	5-Androstene-3β,7α,17β-triol	0.972	2.31	*	0.098	1.19	0.457		0.026	2.41	*	↑
	5-Androstene-3β,7β,17β-triol	1.01	2.80	*	0.100	1.43	0.467		0.027	2.73	*	↑
	5-Androstene-3β,16α,17β-triol	0.813	2.08	*	0.173	4.49	0.812	**	0.022	2.03	*	↑
	Progesterone	0.872	3.81	**	0.167	3.18	0.768	**	0.024	4.19	**	↑
	20α-Dihydroprogesterone	1.049	3.94	**	0.180	4.34	0.841	**	0.028	2.77	*	↑
	20α-Dihydroprogesterone, C	0.747	2.30	*	0.119	1.85	0.555		0.020	1.94	*	↑
	17,20α-Dihydroxy-4-pregnene-3-one	1.226	3.54	**	0.159	2.78	0.743	*	0.033	3.52	**	↑
	17,20α-Dihydroxy-4-pregnene-3-one, C	0.934	3.35	**	0.178	6.53	0.831	**	0.025	3.83	**	↑
	Allopregnanolone	0.758	2.49	*	0.157	4.13	0.735	**	0.021	2.73	*	↑
	Allopregnanolone, C	0.941	3.45	**	0.165	5.01	0.772	**	0.026	2.96	*	↑
	Isopregnanolone	1.061	3.78	**	0.176	4.75	0.824	**	0.029	3.34	**	↑
	Isopregnanolone, C	0.853	2.98	*	0.176	4.26	0.825	**	0.023	3.33	**	↑
	Pregnanolone, C	1.079	3.28	**	0.170	5.81	0.794	**	0.029	3.62	**	↑
	Epipregnanolone, C	0.909	3.19	**	0.184	5.51	0.861	**	0.025	3.88	**	↑
	17-Hydroxyallopregnanolone	1.146	3.83	**	0.187	6.47	0.875	**	0.031	3.97	**	↑
	17-Hydroxyallopregnanolone, C	1.252	8.91	**	0.179	6.82	0.835	**	0.034	5.21	**	↑
	17-Hydroxypregnanolone, C	1.128	4.30	**	0.167	6.11	0.781	**	0.031	6.43	**	↑
	5α,20α-Tetrahydroprogesterone	1.141	5.77	**	0.189	6.83	0.887	**	0.031	3.76	**	↑
	5α-Pregnane-3β,20α-diol	0.762	1.97	*	0.169	3.43	0.788	**	0.021	1.86		↑
	5β,20α-Tetrahydroprogesterone, C	0.704	2.04	*	0.002	0.04	0.008		−0.019	−1.82		↑
	5α-Pregnane-3α,17,20α-triol	1.031	2.89	*	0.165	2.94	0.772	*	0.028	2.53	*	↑
	5α-Pregnane-3β,17,20α-triol	0.884	2.45	*	0.170	4.07	0.797	**	0.024	2.37	*	↑
	5α-Pregnane-3β,17,20α-triol, C	0.794	2.75	*	0.180	5.41	0.799	**	0.022	2.69	*	↑
	5β-Pregnane-3α,17,20α-triol	0.809	2.16	*	0.169	3.22	0.790	**	0.022	2.22	*	↑
	5β-Pregnane-3α,17,20α-triol, C	0.885	3.48	**	0.191	10.62	0.892	**	0.024	4.37	**	↑
	5α-Androstane-3,17-dione	1.051	2.32	*	0.162	2.73	0.758	*	0.029	2.19	*	↑
	Androsterone	1.237	4.43	**	0.174	3.56	0.816	**	0.034	4.49	**	↑
	Epiandrosterone	1.237	5.94	**	0.200	8.10	0.935	**	0.034	4.63	**	↑
	5α-Androstane-3β,17β-diol	1.099	2.61	*	0.174	3.11	0.813	**	0.030	2.17	*	↑
	5β-Androstane-3α,17β-diol	1.4	4.57	**	0.188	5.38	0.880	**	0.038	3.30	**	↑
	11β-Hydroxyepiandrosterone	1.255	3.06	**	0.163	5.15	0.761	**	0.034	2.67	*	↑
	11β-Hydroxyepiandrosterone, C	0.821	2.11	*	0.133	1.82	0.624		0.022	1.85		↑
**Y**	Clozapine (vs. Risperidone), LLR				1.000	14.88	0.793	**				
	R^2^Y = shared variability with explanatory variables (after CV = Q^2^), CV-ANOVA	62.8% (56.2%), F(2, 8) = 5.1, *p* = 0.037										
Number of relevant/total explanatory variables = 39/86; shared variability among explanatory variables (R^2^X) = 58.5%; predictive/orthogonal components = 1/0; sensitivity = 1(1–1); specificity = 0.6(0.171–1.03) (values with 95% CI).												

^a^ R = Component loadings expressed as correlation coefficients with predictive components; LLR = logarithm of likelihood ratio; * *p* < 0.05, ** *p* < 0.01; CV = cross-validation; the *p*-value of CV-ANOVA (analysis of variance testing of cross-validated predictive residuals) indicates the probability level where a model with this F-value may be the result of just chance. Abbreviations: OPLS, orthogonal projections to latent structures; OMR, ordinary multiple regression; R^2^X, fraction of X-variance explained; R^2^Y, fraction of Y-variance explained; Q^2^, predictive ability estimated by cross-validation; CV-ANOVA, cross-validated ANOVA; LLR, logarithm of likelihood ratio. Steroid abbreviations are defined in Table 2. All variables listed in Table 7 reached statistical significance in at least one of the two modeling frameworks (OPLS or OMR). No non-significant predictors (n.s.) were retained in the final models, consistent with the cross-validated OPLS discrimination (CV-ANOVA *p* = 0.037). Arrows indicate direction of group differences: ↑ higher levels in the first group listed; ↓ lower levels in the first group listed.

**Table 8:** Discrimination between clozapine- and risperidone-treated patients based on steroid profiles, a proxy for AKR1D1 activity, evaluated by OPLS and OMR models.

		Orthogonal Predictions to Latent Structure (OPLS) Predictive Component							Ordinary Multiple Regression (OMR)			
	Variable	Variable Importance	t-Statistic		Component Loading	t-Statistic	R ^a^		Regression Coefficient	t-Statistic		
Explanatory variables (**X**)	Age	0.863	2.00	*	−0.432	−2.33	−0.610	*	−0.266	−1.82		↓
	3α,5β-THP/P	0.909	2.64	*	−0.485	−4.19	−0.591	**	−0.280	−3.05	**	↓
	3α,5β,17β-AD/T	1.272	3.93	**	0.574	4.43	0.810	**	0.392	3.32	**	↑
	11β-OH-3α,5β-THA/11β-OH-A	0.9	3.84	**	0.506	5.10	0.714	**	0.277	2.30	*	↑
**Y**	Clozapine (vs. risperidone), LLR				1.000	7.59	0.870	**				
	R^2^Y = shared variability with explanatory variables (after CV = Q^2^), CV-ANOVA	75.7% (57.7%), F(2, 8) = 5.8, *p* = 0.028										
Number of relevant/total explanatory variables = 4/22; shared variability among explanatory variables (R^2^X) = 47.5%; predictive/orthogonal components = 1/0; sensitivity = 1(0.541–1); specificity = 1(0.478–1) (values with 95% CI).												

^a^ R = Component loadings expressed as correlation coefficients with predictive components; LLR = logarithm of likelihood ratio; * *p* < 0.05, ** *p* < 0.01; CV = cross-validation; the *p*-value of CV-ANOVA (analysis of variance testing of cross-validated predictive residuals) indicates the probability level where a model with this F-value may be the result of just chance. Arrows indicate direction of group differences: ↑ higher levels in the first group listed; ↓ lower levels in the first group listed.

**Table 9:** Discrimination between clozapine- and risperidone-treated patients based on 3α/3β steroid molar ratios, a proxy for the balance between AKR1C2 and HSD17B2/HSD17B6 activities, evaluated by OPLS and OMR models.

		Orthogonal Predictions to Latent Structure (OPLS) Predictive Component							Ordinary Multiple Regression (OMR)			
	Variable	Variable Importance	t-Statistic		Component Loading	t-Statistic	R ^a^		Regression Coefficient	t-Statistic		
Explanatory variables (**X**)	Age	0.887	2.04	*	−0.399	−3.77	−0.655	**	−0.196	−2.08	*	↓
	3α,5α-THP/3β,5α-THP, C	0.776	2.17	*	0.406	2.33	0.667	*	0.172	2.43	*	↑
	3α,5β,20α-PD/3β,5β,20α-PD, C	1.233	4.16	**	0.481	3.65	0.789	**	0.273	5.98	**	↑
	3α,5α-THA/3β,5α-THA	0.497	2.11	*	−0.266	−2.65	−0.437	*	−0.110	−1.92	*	↓
	11β-OH-3α,5α-THA/11β-OH-3β,5α-THA	1.26	3.48	**	−0.535	−3.07	−0.878	**	−0.279	−2.67	*	↓
	3α,5β,20α-PD/5β,20α-THP, C	1.12	3.19	**	0.340	3.43	0.558	**	0.248	2.58	*	↑
**Y**	Clozapine (vs. risperidone), LLR				1.000	8.03	0.890	**				
	R^2^Y = shared variability with explanatory variables (after CV = Q^2^), CV-ANOVA	79.2% (69.4%), F(2, 8) = 9.1, *p* = 0.009										
Number of relevant/total explanatory variables = 6/31; shared variability among explanatory variables (R^2^X) = 46.2%; predictive/orthogonal components = 1/0; sensitivity = 1(0.541–1); specificity = 1(0.478–1) (values with 95% CI).												

^a^ R = Component loadings expressed as correlation coefficients with predictive components; LLR = logarithm of likelihood ratio; * *p* < 0.05, ** *p* < 0.01; CV = cross-validation; the *p*-value of CV-ANOVA (analysis of variance testing of cross-validated predictive residuals) indicates the probability level where a model with this F-value may be the result of just chance. Arrows indicate direction of group differences: ↑ higher levels in the first group listed; ↓ lower levels in the first group listed.

**Table 10:** Discrimination between clozapine- and risperidone-treated patients based on steroid profiles, specifically 17β-steroid to 17-oxo ratios as a proxy for the balance between HSD17B3/AKR1C3 and HSD17B2 activities, evaluated by OPLS and OMR models.

		Orthogonal Predictions to Latent Structure (OPLS), Predictive Component							Ordinary Multiple Regression (OMR)			
	Variable	Variable Importance	t-Statistic		Component Loading	t-Statistic	R ^a^		Regression Coefficient	t-Statistic		
Explanatory variables (**X**)	Age	0.867	2.18	*	−0.391	−2.21	−0.600	*	−0.225	−1.79		↓
	5α-DHTC/3α,5α-THA	0.803	2.99	*	−0.551	−5.46	−0.845	**	−0.208	−5.13	**	↓
	5α-DHTCU/3α,5α-THA	0.792	2.65	*	−0.540	−5.13	−0.829	**	−0.205	−4.64	**	↓
	3α,5β,17β-ADCU/3α,5β-THA	1.406	4.01	**	0.563	3.35	0.864	**	0.365	2.65	*	↑
**Y**	Clozapine (vs. risperidone), LLR				1.000	5.52	0.796	**				
	R^2^Y = shared variability with explanatory variables (after CV = Q^2^), CV-ANOVA	63.4% (54.6%), F(2, 8) = 4.8, *p* = 0.042										
Number of relevant/total explanatory variables = 4/18; shared variability among explanatory variables (R^2^X) = 62.7%; predictive/orthogonal components = 1/0; sensitivity = 1(0.541–1); specificity = 0.8(0.284–0.995) (values with 95% CI).												

^a^ R = Component loadings expressed as correlation coefficients with predictive components; LLR = logarithm of likelihood ratio; * *p* < 0.05, ** *p* < 0.01; CV = cross-validation; the *p*-value of CV-ANOVA (analysis of variance testing of cross-validated predictive residuals) indicates the probability level where a model with this F-value may be the result of just chance. Arrows indicate direction of group differences: ↑ higher levels in the first group listed; ↓ lower levels in the first group listed.

**Table 11:** Discrimination between olanzapine- and risperidone-treated patients based on conjugated-to-unconjugated steroid ratios (C/U), a proxy for the balance between SULT2A1 and STS activities, evaluated by OPLS and OMR models.

		Orthogonal Predictions to Latent Structure (OPLS), Predictive Component							Ordinary Multiple Regression (OMR)			
	Variable	Variable Importance	t-Statistic		Component Loading	t-Statistic	R ^a^		Regression Coefficient	t-Statistic		
Explanatory variables (**X**)	Preg, C/U	0.799	2.36	*	−0.283	−3.40	−0.661	**	−0.066	−1.94	*	↓
	20α-DHPreg, C/U	0.917	2.77	*	−0.296	−6.42	−0.687	**	−0.076	−2.30	*	↓
	DHEA, C/U	1.221	2.61	*	−0.285	−3.06	−0.664	**	−0.101	−3.31	**	↓
	3β,16α,17β-AT, C/U	0.902	2.95	*	−0.297	−3.37	−0.684	**	−0.075	−2.40	*	↓
	3β,5α-THP, C/U	0.971	4.49	**	−0.346	−7.19	−0.804	**	−0.081	−2.78	*	↓
	3α,5β-THP, C/U	0.941	4.12	**	−0.291	−4.84	−0.673	**	−0.078	−3.79	**	↓
	5α,20α-THP, C/U	1.14	3.59	**	−0.232	−6.25	−0.540	**	−0.094	−3.22	**	↓
	3β,5α,17,20α-PT, C/U	1.352	3.13	**	−0.269	−3.06	−0.584	**	−0.112	−2.16	*	↓
	3β,5α-THA, C/U	1.203	3.86	**	−0.323	−7.61	−0.748	**	−0.100	−5.24	**	↓
	3α,5β-THA, C/U	0.388	2.80	*	−0.277	−1.85	−0.636		−0.032	−0.50		↓
	3β,5α,17β-AD, C/U	0.853	2.26	*	−0.321	−4.40	−0.745	**	−0.071	−1.77		↓
	3α,5β,17β-AD, C/U	1.075	3.52	**	−0.297	−4.50	−0.686	**	−0.089	−2.50	*	↓
	11β-OH-3β,5α-THA, C/U	0.883	2.61	*	−0.186	−2.41	−0.425	*	−0.073	−2.83	*	↓
**Y**	Olanzapine (vs. risperidone), LLR				1.000	5.70	0.694	**				
	R^2^Y = shared variability with explanatory variables (after CV = Q^2^), CV-ANOVA	48.2% (37.1%), F(2, 16) = 4.8, *p* = 0.024										
Number of relevant/total explanatory variables = 13/33; shared variability among explanatory variables (R^2^X) = 44.1%; predictive/orthogonal components = 1/0; sensitivity = 1(0.768–1); specificity = 0.6(0.147–0.947) (values with 95% CI).												

^a^ R = Component loadings expressed as correlation coefficients with predictive components; LLR = logarithm of likelihood ratio; * *p* < 0.05, ** *p* < 0.01; CV = cross-validation; the *p*-value of CV-ANOVA (analysis of variance testing of cross-validated predictive residuals) indicates the probability level where a model with this F-value may be the result of just chance. Arrows indicate direction of group differences: ↑ higher levels in the first group listed; ↓ lower levels in the first group listed.

**Table 12:** Trends in steroids and steroid molar ratios that may reflect functioning of steroidogenic enzymes and balances between them (evaluated by Wilcoxon’s robust test).

	Clozapine (vs. Olanzapine)				Clozapine (vs. Risperidone)				Olanzapine (vs. Risperidone)			
Variable	Clo < Ola	Clo ~ Ola	Clo > Ola	*p*-Value	Clo < Ris	Clo ~ Ris	Clo > Ris	*p*-Value	Ola < Ris	Ola ~ Ris	Ola > Ris	*p*-Value
1. Steroids	9 (10.6%)	64 (75.3%)	12 (14.1%)	0.515	**1 (1.2%)**	**44 (51.8%)**	**40 (47.1%)**	**<0.001**	**0 (0%)**	**43 (50.6%)**	**42 (49.4%)**	**<0.001**
2. § Unconjugated steroids	8 (16.3%)	36 (73.5%)	5 (10.2%)	0.409	**0 (0%)**	**23 (46.9%)**	**26 (53.1%)**	**<0.001**	**0 (0%)**	**15 (30.6%)**	**34 (69.4%)**	**<0.001**
3. **§ Conjugated steroids**	**1 (2.8%)**	**28 (77.8%)**	**7 (19.4%)**	**0.035**	**1 (2.8%)**	**21 (58.3%)**	**14 (38.9%)**	**0.001**	**0 (0%)**	**28 (77.8%)**	**8 (22.2%)**	**0.005**
4. ***CYP17A1*, hydroxylase + lyase** ^c^	**6 (26.1%)**	**17 (73.9%)**	**0 (0%)**	**0.015**	**5 (21.7%)**	**18 (78.3%)**	**0 (0%)**	**0.027**	0 (0%)	23 (100%)	0 (0%)	1
5. *CYP17A1*, hydroxylase ^c^	0 (0%)	13 (81.3%)	3 (18.8%)	0.09	1 (6.3%)	14 (87.5%)	1 (6.3%)	1	0 (0%)	16 (100%)	0 (0%)	1
6. ***CYP17A1*, lyase** ^c^	**9 (56.3%)**	**7 (43.8%)**	**0 (0%)**	**0.003**	**4 (25%)**	**12 (75%)**	**0 (0%)**	**0.049**	1 (6.3%)	15 (93.8%)	0 (0%)	0.349
7. *HSD3B1*, *HSD3B2*	0 (0%)	13 (100%)	0 (0%)	1	1 (7.7%)	10 (76.9%)	2 (15.4%)	0.597	1 (7.7%)	8 (61.5%)	4 (30.8%)	0.193
8. *SULT2A1* (vs. *STS*) ^c^	2 (6.3%)	24 (75%)	6 (18.8%)	0.161	**9 (28.1%)**	**23 (71.9%)**	**0 (0%)**	**0.003**	**14 (43.8%)**	**18 (56.3%)**	**0 (0%)**	**<0.001**
9. *HSD11B1* (vs. *HSD11B2*) ^c^	0 (0%)	1 (25%)	3 (75%)	0.124	0 (0%)	3 (75%)	1 (25%)	0.453	0 (0%)	4 (100%)	0 (0%)	1
10. *CYP11B1*	0 (0%)	7 (100%)	0 (0%)	1	0 (0%)	7 (100%)	0 (0%)	1	0 (0%)	7 (100%)	0 (0%)	1
11. *CYP7B1*, *CYP3A4*, *CYP3A7*	0 (0%)	7 (87.5%)	1 (12.5%)	0.382	0 (0%)	8 (100%)	0 (0%)	1	0 (0%)	8 (100%)	0 (0%)	1
12. ***SRD5A1*, *SRD5A2***	**0 (0%)**	**14 (66.7%)**	**7 (33.3%)**	**0.009**	**0 (0%)**	**12 (57.1%)**	**9 (42.9%)**	**0.003**	1 (4.8%)	20 (95.2%)	0 (0%)	0.341
13. *AKR1D1*	0 (0%)	20 (95.2%)	1 (4.8%)	0.341	1 (4.8%)	18 (85.7%)	2 (9.5%)	0.583	0 (0%)	20 (95.2%)	1 (4.8%)	0.341
14. *AKR1C1* (vs. *HSD17B2*) ^c^	2 (10.5%)	15 (78.9%)	2 (10.5%)	1	0 (0%)	19 (100%)	0 (0%)	1	0 (0%)	19 (100%)	0 (0%)	1
15. *AKR1C2* (vs. *HSD17B2*, *HSD17B6*) ^c^	3 (10%)	21 (70%)	6 (20%)	0.324	2 (6.7%)	24 (80%)	4 (13.3%)	0.423	1 (3.3%)	28 (93.3%)	1 (3.3%)	1
16. ***HSD17B3*, *AKR1C3* (vs. *HSD17B2*)** ^c^	**0 (0%)**	**9 (52.9%)**	**8 (47.1%)**	**0.005**	3 (17.6%)	13 (76.5%)	1 (5.9%)	0.333	**7 (41.2%)**	**10 (58.8%)**	**0 (0%)**	**0.009**

Note. Clo—clozapine; Ola—olanzapine; Ris—risperidone. Trends were evaluated by Wilcoxon’s robust test. Rows marked § are complementary subsets of “Steroids” (*n* = 85): “Unconjugated steroids” and “Conjugated steroids”. Rows marked ^c^ share metabolites and are therefore not independent. The three CYP17A1 ratios are algebraically linked through a common intermediate: “CYP17A1, hydroxylase” is the ratio of C21 17-hydroxy steroids to their C21 deoxy analogs; “CYP17A1, lyase” is the ratio of C19 steroids to their C21 17-hydroxy analogues; and “CYP17A1, hydroxylase + lyase” is the ratio of C19 steroids to their C21 deoxy analogues; the combined ratio thus shares its numerator with the lyase ratio and its denominator with the hydroxylase ratio, with the C21 17-hydroxy intermediate being the product of the hydroxylase step and the substrate of the lyase step simultaneously. Likewise, the enzyme-ratio rows share common numerator or denominator metabolites (e.g., HSD17B2, common to the AKR1C1, AKR1C2, and HSD17B3/AKR1C3 ratios), and substrate–product pairs such as SRD5A1 and SRD5A2, which are biochemically coupled. Because these rows are nested, overlapping, or algebraically linked and strongly intercorrelated, the per-row counts and *p*-values are not statistically independent, are not additive, and should be interpreted as coordinated, pathway-level directional trends rather than the number of independent significant steroids. Bold formatting indicates variables with *p* < 0.05. Rows marked with § denote complementary subsets of “Steroids” (n = 85): “Unconjugated steroids” and “Conjugated steroids.” The numbers of altered steroids reported here (trend-based test) differ from those in Table 2 (OPLS/OMR), because the two methods use different counting frameworks and are not directly comparable.

**Table 13:** Age distribution and standardized between-group differences (Hedges’ g) among patients treated with clozapine, olanzapine, or risperidone.

Antipsychotic Drug	Mean ± SD (Years)	Median (Quartiles)	Range (Min–Max)
Clozapine (Clo)	24 ± 3.8	23.1 (21.9, 27)	19–29.7
Olanzapine (Ola)	27 ± 5.7	27.4 (22.3, 30.7)	19.4–38.9
Risperidone (Ris)	32.6 ± 8.2	36 (33.4, 36.4)	18.4–39
Comparison, effect size (95% CI)	Hedges’ g (Bias-Corrected)		
Clo vs. Ola	−0.548 (−1.48, 0.39)		
Clo vs. Ris	−1.277 (−2.51, −0.04)		
Ola vs. Rise	−0.06 (−1.04, 0.92)		
ANOVA Omnibus test	F(2, 22) = 3.04, *p* = 0.069, η_p_^2^ = 0.216, ω^2^ = 0.14		

The table reports mean ± SD, median (Q1, Q3), and range (min–max), together with bias-corrected Hedges’ g (95% CI) for each pairwise comparison; negative values indicate lower mean age in the first group. Effect sizes: negligible (|g| < 0.20), small (0.20–0.49), medium (0.50–0.79), or large (≥0.80); a 95% CI excluding zero indicates significance. ηp^2^ and ω^2^ are ANOVA effect-size estimates (ηp^2^ > 0.14 large). Despite a non-significant omnibus test (*p* = 0.069), the large ηp^2^ (0.216) and clozapine-vs-risperidone contrast (g = −1.28) indicate a non-trivial age difference; age was retained as a covariate in all analyses. Abbreviations: ANOVA—one-way analysis of variance; ηp^2^—partial eta squared (equal to η^2^ in one-way ANOVA); SD—standard deviation; Q1, Q3—25th/75th percentiles; CI—confidence interval; Clo—clozapine; Ola—olanzapine; Ris—risperidone.

## Data Availability

The datasets generated and analyzed during the current study are not publicly available due to ethical and privacy restrictions but are available from the corresponding author upon reasonable request.

## Supplementary Materials

The following supporting information can be downloaded at: https://www.mdpi.com/article/10.3390/ijms27198909/s1.
