# Supplementary material for: Steroidomic Differences Among Clozapine, Olanzapine, and Risperidone in Male Patients with First-Episode Psychosis, Including a Treatment-Resistant Schizophrenia Subgroup
Source: Int J Mol Sci. 2026 Oct 7;27(19):8909. doi: 10.3390/ijms27198909 (PMC13665165; doi:10.3390/ijms27198909)
Supplement: Supplementary file 1 [file ijms-27-08909-s001.zip › Supplementary Table S1 covariates.pdf]

Table S1. Discrimination between antipsychotic treatment groups based on steroid profiles, evaluated by OPLS and OMR models with relevant clinical covariates

Subtable S1A. Discrimination between **clozapine- and olanzapine**-treated patients based on steroid profiles, evaluated by OPLS and OMR models with **change in CPZeq** after treatment as a covariate

		Orthogonal predictions to latent structure (**OPLS**), **predictive component**					Ordinary multiple regression (**OMR**)		
	**Variable**	Variable importance	t-statistic	Component loading	t-statistic	R[a]	Regression coefficient	t-statistic	
EXPLANATORY VARIABLES (X)	CPZeq21	1.324	2.25*	0.321	2.26	0.682*	0.407	4.96**	↑
	DHEA	0.777	2.14*	-0.180	-1.99	-0.342*	0.063	0.94	↓
	7-oxo-DHEA	1.073	2.27*	0.248	2.05	0.527*	0.165	1.51	↑
	17-OH-20α-DHPC	0.927	2.15*	-0.216	-2.24	-0.452*	-0.265	-3.25**	↓
	A	1.224	3.16**	0.283	2.97	0.604*	0.117	2.18*	↑
	5α-DHT	0.882	2.98*	0.210	2.94	0.448*	0.030	0.44	↑
	3α,5α,17-PD	0.779	2.40*	0.182	2.19	0.391*	0.022	0.31	↑
	3α,5α,17-PDC	0.835	2.47*	0.195	2.23	0.418*	-0.032	-0.48	↑
	3α,5β,17-PDC	0.674	3.23**	0.158	3.38	0.338**	-0.006	-0.07	↑
	5α,20α-THPC	0.839	2.00*	-0.174	-1.77	-0.371	-0.193	-1.44	↓
	3β,5α,17,20α-PTC	0.973	3.25**	0.226	2.73	0.483*	0.021	0.64	↑
	3α,5β,17,20α-PTC	0.942	2.90*	0.218	2.83	0.467*	0.073	1.40	↑
	3β,5α-THAC	0.868	2.08*	0.207	2.11	0.446*	0.015	0.13	↑
	3α,5β-THA	0.912	2.30*	-0.209	-2.44	-0.448*	-0.118	-1.70	↓
	3α,5α,17β-ADC	0.935	5.31**	0.233	4.38	0.501**	0.019	0.20	↑
	3β,5α,17β-AD	1.151	5.38**	0.271	5.19	0.579**	0.074	0.71	↑
	3α,5β,17β-AD	1.376	6.74**	0.337	5.51	0.715**	0.229	4.07**	↑
	E	1.176	5.35**	-0.283	-5.46	-0.582**	-0.089	-1.83	↓
Y	Clozapine (vs. Olanzapine), LLR			1.000	16.78	0.979**			
	R^2Y=shared variability with explanatory variables (after CV=Q^2), CV-ANOVA	95.9% (82%), F(6, 12)=8.4, p<0.001							

Number of relevant/total explanatory variables=18/87, Shared variability among explanatory variables (R^2X)=62.4%, Predictive/orthogonal components=1/2, Sensitivity=1(0.478-1), Specificity=1(0.478-1) (values with 95% CI)

[a]*R=Component loadings expressed as correlation coefficients with predictive components; LLR=logarithm of likelihood ratio; *p<0.05, **p<0.01; CV=cross validation; The p-value of CV-ANOVA (Analysis of variance testing of Cross-Validated predictive residuals) indicates the probability level where a model with this F-value may be the result of just chance.*

Subtable S1B. Discrimination between clozapine- and olanzapine-treated patients based on steroid profiles, evaluated by OPLS and OMR models with basal PANSS-P and post-treatment change in PANSS-P included as covariates

		Orthogonal predictions to latent structure (**OPLS**), **predictive component**					Ordinary multiple regression (**OMR**)		
	**Variable**	Variable importance	t-statistic	Component loading	t-statistic	R[a]	Regression coefficient	t-statistic	
EXPLAINATORY VARIABLES (X)	PANSSP1	0.715	2.98*	0.209	7.23	0.600**	0.037	1.83	↑
	DHEA	0.94	2.37*	-0.147	-2.05	-0.423*	-0.048	-1.75	↓
	7-oxo-DHEA	0.989	2.44*	-0.232	-3.80	-0.584**	-0.051	-2.60*	↓
	17-OH-20α-DHPC	1.127	3.36**	0.239	3.55	0.687**	0.058	2.33*	↑
	A	1.059	3.95**	-0.080	-0.55	-0.228	-0.054	-1.95*	↓
	5α-DHT	1.088	3.34**	0.240	4.00	0.691**	0.056	1.92*	↑
	3α,5β,20α-PDC	0.901	2.71*	0.266	5.05	0.766**	0.046	2.14*	↑
	3α,5α,17-PD	0.827	2.64*	0.209	5.03	0.600**	0.042	2.19*	↑
	3α,5α,17-PDC	0.985	1.98*	0.261	3.31	0.751**	0.050	1.80	↑
	3α,5β,17-PDC	0.613	2.32*	0.211	5.32	0.608**	0.031	1.47	↑
	3α,5α,20α-PD	0.867	2.13*	0.230	7.23	0.663**	0.044	2.19*	↑
	3α,5α,20α-PDC	0.932	1.98*	0.190	4.38	0.546**	0.048	2.16*	↑
	3β,5α,17,20α-PTC	1.141	3.36**	0.291	7.38	0.836**	0.058	3.84**	↑
	3α,5β,17,20α-PTC	1.157	3.32**	0.251	5.25	0.722**	0.059	2.83*	↑
	3β,5α-THAC	0.819	2.11*	0.265	3.85	0.763**	0.042	1.95*	↑
	3α,5β-THA	1.042	2.04*	-0.121	-1.17	-0.348	-0.053	-1.91*	↓
	3α,5α,17β-ADC	0.68	2.03*	0.195	2.65	0.562*	0.035	1.38	↑
	3β,5α,17β-AD	1.043	3.01**	0.263	4.31	0.756**	0.053	1.87	↑
	3α,5β,17β-AD	1.466	4.30**	0.236	5.45	0.678**	0.075	2.48*	↑
	E	1.333	4.82**	-0.265	-5.11	-0.665**	-0.068	-3.62**	↑
	11β-OH-3β,5α-THAC	0.846	2.16*	0.241	3.90	0.692**	0.043	3.28**	↑
Y	Clozapine (vs. Olanzapine), LLR			1.000	9.20	0.674**			
	R^2Y=shared variability with explanatory variables (after CV=Q^2), CV-ANOVA	45.4% (31.7%), F(2, 17)=4, p=0.038							

Number of relevant/total explanatory variables=21/88, Shared variability among explanatory variables (R^2X)=41.4%, Predictive/orthogonal components=1/0, Sensitivity=0.5(0.118-0.882), Specificity=0.5(0.118-0.882) (values with 95% CI)

*[a]R=Component loadings expressed as correlation coefficients with predictive components; LLR=logarithm of likelihood ratio; *p<0.05, **p<0.01; CV=cross validation; The p-value of CV-ANOVA (Analysis of variance testing of Cross-Validated predictive residuals) indicates the probability level where a model with this F-value may be the result of just chance.*

Subtable S1C. Discrimination between olanzapine- and risperidone-treated patients based on steroid profiles, evaluated by OPLS and OMR models with basal PANSS-P and post-treatment change in PANSS-P included as covariates

		Orthogonal predictions to latent structure (**OPLS**), **predictive component**					Ordinary multiple regression (**OMR**)		
	**Variable**	Variable importance	t-statistic	Component loading	t-statistic	R[a]	Regression coefficient	t-statistic	
EXPLAINATORY VARIABLES (X)x	PANSSP1	1.071	2.01*	-0.067	-1.25	-0.273	-0.026	-2.28*	↓
	Preg	1.050	3.28**	0.201	5.11	0.813**	0.025	2.36*	↑
	20α-DHPreg	0.996	2.03*	0.171	4.06	0.692**	0.024	1.52	↑
	17-OH-Preg	0.897	2.98*	0.160	4.91	0.648**	0.022	2.38*	↑
	17-OH-PregC	1.155	6.18**	0.179	9.46	0.724**	0.028	2.91*	↑
	DHEA	1.283	4.07**	0.192	4.42	0.779**	0.031	3.03**	↑
	7α-OH-DHEA	1.243	5.44**	0.223	9.44	0.903**	0.030	3.70**	↑
	7β-OH-DHEA	1.173	5.70**	0.207	8.49	0.838**	0.028	2.62*	↑
	3β,7α,17β-AT	1.013	2.48*	0.188	2.90	0.761*	0.025	4.15**	↑
	3β,7β,17β-AT	0.766	2.19*	0.171	2.95	0.694*	0.019	2.65*	↑
	20α-DHP	0.917	2.75*	0.208	4.75	0.844**	0.022	2.12*	↑
	20α-DHPC	0.660	2.16*	0.040	1.59	0.160	0.016	1.92*	↑
	16α-OH-P	0.624	2.22*	0.131	4.88	0.533**	0.015	2.26*	↑
	17-OH-20α-DHP	1.106	2.94*	0.180	3.77	0.728**	0.027	3.71**	↑
	A	1.216	3.89**	0.171	3.15	0.692**	0.030	4.48**	↑
	5α-DHP	1.044	3.27**	0.176	3.49	0.711**	0.025	2.47*	↑
	3α,5α-THP	0.728	4.24**	0.188	4.93	0.762**	0.018	3.89**	↑
	3α,5α-THPC	0.885	2.14*	0.199	4.55	0.805**	0.021	1.58	↑
	3β,5α-THP	1.063	6.83**	0.205	10.86	0.832**	0.026	2.98*	↑
	3β,5α-THPC	0.866	3.02**	0.172	7.26	0.698**	0.021	1.84	↑
	3α,5β-THP	0.666	1.93*	0.133	2.12	0.540*	0.016	1.73	↑
	3α,5β-THPC	0.716	2.51*	0.139	3.27	0.565**	0.017	1.72	↑
	3β,5β-THPC	0.875	2.41*	0.162	4.16	0.655**	0.021	1.63	↑
	3α,5α,17-PDC	0.861	3.70**	0.194	7.23	0.788**	0.021	2.72*	↑
	3α,5β,17-PDC	0.871	3.92**	0.135	3.81	0.548**	0.021	5.14**	↑
	5α,20α-THP	1.127	3.51**	0.217	7.59	0.878**	0.027	2.06*	↑
	3α,5α,17,20α-PT	1.060	4.45**	0.179	4.41	0.726**	0.026	3.14**	↑
	5α-DHA	1.145	7.26**	0.141	4.57	0.572**	0.028	3.13**	↑
	3α,5α-THA	1.077	3.07**	0.076	1.50	0.306	0.026	3.04**	↑
	3β,5α-THA	1.283	4.02**	0.164	3.19	0.667**	0.031	4.47**	↑
	3α,5β-THA	0.908	1.95*	0.123	3.49	0.498**	0.022	1.55	↑
	3α,5α,17β-AD	0.920	2.13*	0.121	4.23	0.490**	0.022	2.27*	↑
	3β,5α,17β-AD	0.798	2.48*	0.127	2.55	0.515*	0.019	1.64	↑
	F	1.094	3.29**	0.171	15.76	0.703**	0.027	4.25**	↑
	B	0.987	4.09**	0.181	5.90	0.732**	0.024	4.19**	↑
	11β-OH-3α,5α-THA	1.226	2.75*	0.188	3.96	0.763**	0.030	3.76**	↑
	11β-OH-3β,5α-THA	1.036	2.31*	0.193	3.12	0.784**	0.025	2.30*	↑
Y	Olanzapine (vs. Risperidone), LLR			1.000	8.33	0.598**			
	R^2Y=shared variability with explanatory variables (after CV=Q^2), CV-ANOVA			35.8% (26.7%), F(2, 16)=2.9, p=0.084					

Number of relevant/total explanatory variables=37/88, Shared variability among explanatory variables (R^2X)=47%, Predictive/orthogonal components=1/0, Sensitivity=0.857(0.572-0.982), Specificity=0.857(0.572-0.982) (values with 95% CI)

*[a]R=Component loadings expressed as correlation coefficients with predictive components; LLR=logarithm of likelihood ratio; *p<0.05, **p<0.01; CV=cross validation; The p-value of CV-ANOVA (Analysis of variance testing of Cross-Validated predictive residuals) indicates the probability level where a model with this F-value may be the result of just chance.*

Subtable S1D. Discrimination between olanzapine- and risperidone-treated patients based on steroid profiles, evaluated by OPLS and OMR models with basal PANSS-Σ and post-treatment change in PANSS-Σ included as covariates

		Orthogonal predictions to latent structure (**OPLS**), **predictive component**					Ordinary multiple regression (**OMR**)		
	**Variable**	Variable importance	t-statistic	Component loading	t-statistic	R[a]	Regression coefficient	t-statistic	
EXPLANATORY VARIABLES (X)	PANSSsum21	1.046	2.02*	0.044	0.55	0.175	0.026	2.65*	↑
	Preg	1.048	3.36**	0.200	4.75	0.803**	0.026	2.41*	↑
	20α-DHPreg	0.995	2.03*	0.169	3.84	0.679**	0.025	1.54	↑
	17-OH-Preg	0.895	3.14**	0.161	4.71	0.647**	0.022	2.32*	↑
	17-OH-PregC	1.153	5.76**	0.180	9.28	0.724**	0.029	3.05**	↑
	DHEA	1.281	3.89**	0.191	4.15	0.768**	0.032	3.06**	↑
	7α-OH-DHEA	1.241	5.52**	0.227	9.54	0.909**	0.031	3.85**	↑
	7β-OH-DHEA	1.171	5.40**	0.210	8.54	0.844**	0.029	2.69*	↑
	3β,7α,17β-AT	1.011	2.46*	0.192	2.88	0.772*	0.025	4.06**	↑
	3β,7β,17β-AT	0.764	2.19*	0.176	2.97	0.706*	0.019	2.65*	↑
	20α-DHP	0.916	2.78*	0.211	4.73	0.849**	0.023	2.22*	↑
	20α-DHPC	0.659	2.20*	0.042	1.72	0.169	0.016	1.87	↑
	16α-OH-P	0.623	2.35*	0.135	5.08	0.541**	0.015	2.16*	↑
	17-OH-20α-DHP	1.105	2.98*	0.182	3.69	0.729**	0.027	3.77**	↑
	A	1.214	4.21**	0.177	3.17	0.712**	0.030	4.15**	↑
	5α-DHP	1.043	3.17**	0.178	3.41	0.713**	0.026	2.46*	↑
	3α,5α-THP	0.727	4.25**	0.190	4.89	0.764**	0.018	4.19**	↑
	3α,5α-THPC	0.884	2.05*	0.200	4.52	0.802**	0.022	1.64	↑
	3β,5α-THP	1.062	6.73**	0.205	9.86	0.822**	0.026	3.12**	↑
	3β,5α-THPC	0.865	2.91*	0.171	7.27	0.686**	0.021	1.91*	↑
	3α,5β-THP	0.665	1.92*	0.132	2.08	0.531*	0.016	1.75	↑
	3α,5β-THPC	0.715	2.46*	0.139	3.25	0.556**	0.018	1.76	↑
	3β,5β-THPC	0.873	2.36*	0.159	3.96	0.638**	0.022	1.67	↑
	3α,5α,17-PDC	0.86	3.86**	0.198	8.48	0.794**	0.021	2.68*	↑
	3α,5β,17-PDC	0.869	4.03**	0.136	3.87	0.544**	0.022	5.01**	↑
	5α,20α-THP	1.125	3.42**	0.219	7.48	0.879**	0.028	2.14*	↑
	3α,5α,17,20α-PT	1.059	4.44**	0.182	4.35	0.731**	0.026	3.18**	↑
	5α-DHA	1.144	7.24**	0.145	4.66	0.580**	0.028	3.15**	↑
	3α,5α-THA	1.075	2.99*	0.069	1.24	0.277	0.027	3.05**	↑
	3β,5α-THA	1.281	3.85**	0.164	3.08	0.657**	0.032	4.94**	↑
	3α,5α,17β-AD	0.919	2.14*	0.123	4.46	0.493**	0.023	2.17*	↑
	3β,5α,17β-AD	0.797	2.44*	0.127	2.53	0.511*	0.020	1.69	↑
	F	1.085	3.47**	0.177	13.72	0.714**	0.027	4.04**	↑
	B	0.985	4.33**	0.187	5.58	0.750**	0.024	4.09**	↑
	11β-OH-3α,5α-THA	1.225	2.78*	0.194	4.03	0.778**	0.030	3.61**	↑
	11β-OH-3β,5α-THA	1.034	2.37*	0.198	3.18	0.795**	0.026	2.25*	↑
Y	Olanzapine (vs. Risperidone), LLR			1.000	8.18	0.597**			
	R²Y=shared variability with explanatory variables (after CV=Q²), CV-ANOVA			35.6% (27.2%), F(2, 16)=3, p=0.079					

Number of relevant/total explanatory variables=36/88, Shared variability among explanatory variables (R^2X)=47.7%, Predictive/orthogonal components=1/0, Sensitivity=0.929(0.661-0.998), Specificity=0.929(0.661-0.998) (values with 95% CI)

*[a]R=Component loadings expressed as correlation coefficients with predictive components; LLR=logarithm of likelihood ratio; *p<0.05, **p<0.01; CV=cross validation; The p-value of CV-ANOVA (Analysis of variance testing of Cross-Validated predictive residuals) indicates the probability level where a model with this F-value may be the result of just chance.*
