# Supplementary material for: Steroidomic Differences Among Clozapine, Olanzapine, and Risperidone in Male Patients with First-Episode Psychosis, Including a Treatment-Resistant Schizophrenia Subgroup
Source: Int J Mol Sci. 2026 Oct 7;27(19):8909. doi: 10.3390/ijms27198909 (PMC13665165; doi:10.3390/ijms27198909)
Supplement: Supplementary file 1 [file ijms-27-08909-s001.zip › Supplementary Table S2 ANOVA.pdf]

Supplementary Table S2. Age, BMI, and steroid levels in men with schizophrenia treated with clozapine, olanzapine, and risperidone, as evaluated by age-adjusted ANCOVA and LSD multiple comparisons (age differences were evaluated using one-way ANOVA and LSD multiple comparisons). Data are shown as re-transformed means with their 95% confidence intervals.

**Variable**	Clozapine	Olanzapine	Risperidone
Age [years]	23.7 (20.9, 26.9)	26.3 (24.2, 28.7)	31 (26.8, 36.4)
	Drug: $F=2.1$, $p=0.147$, $\eta p^2=0.16$;		
BMI [kg/m^2]	24.5 (22.4, 27.1)	27.9 (26.1, 29.9)	26.1 (23.5, 29.5)
	Drug: $F=1.4$, $p=0.281$, $\eta_p^2=0.114$; Age: $F=0.7$, $p=0.418$, $\eta_p^2=0.0315$		
	(a) **Δ⁵-pregnanes**		
Pregnenolone [nM]	1.82 (1.2, 2.61)	2.07 (1.62, 2.58)	1.18 (0.666, 1.87)
	Drug: $F=1.3$, $p=0.305$, $\eta_p^2=0.107$; Age: $F=1.6$, $p=0.219$, $\eta_p^2=0.0711$		
Pregnenolone sulfate [nM]	503 (376, 662)	514 (425, 616)‡	299 (204, 423)
	Drug: $F=2.1$, $p=0.152$, $\eta_p^2=0.172$; Age: $F=6.5$, $p=0.02$, $\eta_p^2=0.244$		
20α-Dihydropregnenolone [nM]	3.47 (2.7, 4.24)	3.26 (2.78, 3.75)	2.22 (1.37, 3.08)
	Drug: $F=1.4$, $p=0.263$, $\eta_p^2=0.12$; Age: $F=1.1$, $p=0.297$, $\eta_p^2=0.0517$		
20α-Dihydropregnenolone sulfate [μM]	2.05 (1.44, 2.97)	1.76 (1.41, 2.2)	1.44 (0.976, 2.13)
	Drug: $F=0.5$, $p=0.644$, $\eta_p^2=0.0411$; Age: $F=3.3$, $p=0.085$, $\eta_p^2=0.135$		
17-Hydroxypregnenolone [nM]	13 (9.18, 18)	16.5 (13.4, 20.3)	10.4 (6.94, 15.1)
	Drug: $F=1.4$, $p=0.263$, $\eta_p^2=0.12$; Age: $F=4.1$, $p=0.055$, $\eta_p^2=0.164$		
17-Hydroxypregnenolone sulfate [nM]	41 (29.6, 56.2)	42.5 (34.7, 51.9)	24.7 (16.4, 36)
	Drug: $F=1.8$, $p=0.194$, $\eta_p^2=0.145$; Age: $F=0.2$, $p=0.691$, $\eta_p^2=0.00768$		
16α-Hydroxypregnenolone [nM]	0.608 (0.443, 0.817)	0.742 (0.61, 0.896)	0.43 (0.29, 0.614)
	Drug: $F=2.1$, $p=0.154$, $\eta_p^2=0.171$; Age: $F=3.7$, $p=0.07$, $\eta_p^2=0.155$		
	(b) **Δ⁵-androstanes**		
**Dehydroepiandrosterone (DHEA) [nM]**	**9.81 (7.45, 12.6)****	**18 (15.6, 20.7)‡‡**	**9.94 (7.36, 13.1)**
	**Drug: $F=7.6$, $p=0.004$, $\eta_p^2=0.432$; Age: $F=8$, $p=0.01$, $\eta_p^2=0.286$**		
DHEA sulfate [μM]	8.38 (6.2, 11.6)	7.5 (6.21, 9.11)	7.42 (5.34, 10.5)
	Drug: $F=0.1$, $p=0.895$, $\eta_p^2=0.0105$; Age: $F=1.3$, $p=0.272$, $\eta_p^2=0.0572$		
7α-Hydroxy-DHEA [nM]	1.23 (0.845, 1.71)	1.75 (1.43, 2.12)‡	0.927 (0.577, 1.38)
	Drug: $F=2.7$, $p=0.091$, $\eta_p^2=0.204$; Age: $F=1.9$, $p=0.179$, $\eta_p^2=0.0844$		
7-oxo-DHEA [nM]	0.149 (0.0613, 0.331)	0.502 (0.302, 0.828)	0.302 (0.111, 0.779)
	Drug: $F=1.9$, $p=0.184$, $\eta_p^2=0.163$; Age: $F=1$, $p=0.326$, $\eta_p^2=0.0508$		
7β-Hydroxy-DHEA [nM]	0.804 (0.601, 1.03)	0.852 (0.718, 0.995)	0.523 (0.335, 0.741)
	Drug: $F=1.8$, $p=0.196$, $\eta_p^2=0.144$; Age: $F=0.8$, $p=0.38$, $\eta_p^2=0.0368$		
Androstenediol [nM]	3.55 (2.6, 4.95)	3.42 (2.82, 4.2)	2.45 (1.77, 3.45)
	Drug: $F=0.9$, $p=0.437$, $\eta_p^2=0.0759$; Age: $F=4.9$, $p=0.038$, $\eta_p^2=0.19$		
Androstenediol sulfate [μM]	2.92 (1.94, 4.45)	2.14 (1.65, 2.78)	2.26 (1.42, 3.59)
	Drug: $F=0.5$, $p=0.645$, $\eta_p^2=0.0408$; Age: $F=7.2$, $p=0.014$, $\eta_p^2=0.254$		
5-Androstene-3β,7α,17β-triol [nM]	0.756 (0.601, 0.911)	0.737 (0.631, 0.842)	0.539 (0.367, 0.711)
	Drug: $F=1.2$, $p=0.33$, $\eta_p^2=0.11$; Age: $F=1.3$, $p=0.275$, $\eta_p^2=0.0623$		
5-Androstene-3β,7β,17β-triol [nM]	0.411 (0.311, 0.516)	0.37 (0.308, 0.435)	0.301 (0.199, 0.412)
	Drug: $F=0.6$, $p=0.582$, $\eta_p^2=0.0502$; Age: $F=2.8$, $p=0.112$, $\eta_p^2=0.116$		
5-Androstene-3β,16α,17β-triol [nM]	0.196 (0.101, 0.337)	0.248 (0.172, 0.345)	0.184 (0.0848, 0.338)
	Drug: $F=0.9$, $p=0.432$, $\eta_p^2=0.0769$; Age: $F=24.9$, $p<0.001$, $\eta_p^2=0.543$		

5-Androstene-3β,16α,17β-triol sulfate [nM]	188 (119, 302)	161 (122, 216)	190 (115, 323)
	Drug: F=0.1, p=0.873, η_p^2=0.0129; Age: F=3.2, p=0.089, η_p^2=0.132		
(c) **Δ^4-pregnanes**			
Progesterone [pM]	136 (57.9, 382)	130 (73.6, 247)	42.6 (16, 119)
	Drug: F=1.1, p=0.362, η_p^2=0.101; Age: F=0, p=0.987, η_p^2=0.0000154		
20α-Dihydroprogesterone [pM]	202 (124, 299)	196 (145, 254)	101 (43.5, 181)
	Drug: F=1.2, p=0.334, η_p^2=0.0992; Age: F=2.5, p=0.128, η_p^2=0.107		
Conjugated 20α-dihydroprogesterone [nM]	1.42 (0.956, 2.15)	1.48 (1.15, 1.92)	1.11 (0.721, 1.74)
	Drug: F=0.3, p=0.713, η_p^2=0.0317; Age: F=0.7, p=0.399, η_p^2=0.0341		
17-Hydroxyprogesterone [nM]	3.56 (2.83, 4.44)	3.94 (3.41, 4.54)	2.99 (2.28, 3.85)
	Drug: F=1, p=0.383, η_p^2=0.0916; Age: F=8.3, p=0.009, η_p^2=0.294		
16α-Hydroxyprogesterone [nM]	0.699 (0.523, 0.933)	0.726 (0.604, 0.871)	0.61 (0.439, 0.842)
	Drug: F=0.2, p=0.791, η_p^2=0.0221; Age: F=4.7, p=0.041, η_p^2=0.183		
17,20α-Dihydroxy-4-pregnene-3-one [nM]	2.19 (1.6, 2.95)†	1.9 (1.56, 2.31)	1.08 (0.709, 1.57)
	Drug: F=2.4, p=0.115, η_p^2=0.186; Age: F=0.3, p=0.583, η_p^2=0.0146		
Conjugated 17,20α-dihydroxy-4-pregnene-3-one [nM]	16.7 (11.1, 25.5)*, †	8.49 (6.58, 10.9)	6.11 (3.8, 9.58)
	Drug: F=3.1, p=0.066, η_p^2=0.229; Age: F=0, p=0.861, η_p^2=0.00149		
(d) **Δ^4-androstanes**			
Androstenedione [nM]	3.5 (2.87, 4.29)*	5.18 (4.52, 5.96)‡	3.3 (2.65, 4.13)
	Drug: F=4.9, p=0.018, η_p^2=0.318; Age: F=2.1, p=0.162, η_p^2=0.0908		
Testosterone [nM]	16.8 (12.5, 22.5)	15.4 (12.7, 18.5)	14.4 (10.3, 20)
	Drug: F=0.1, p=0.881, η_p^2=0.012; Age: F=1.7, p=0.213, η_p^2=0.0727		
Conjugated testosterone [nM]	16.5 (10, 25.6)	9.44 (6.57, 13.1)	13 (7.12, 21.8)
	Drug: F=1.1, p=0.364, η_p^2=0.0918; Age: F=0.1, p=0.712, η_p^2=0.0066		
(e) **5α-/5β-reduced pregnanes**			
5α-Dihydroprogesterone [pM]	36.1 (17.4, 69.8)	41.3 (26.6, 62.6)	15.3 (5.17, 35)
	Drug: F=1.2, p=0.319, η_p^2=0.103; Age: F=0, p=0.987, η_p^2=0.0000129		
Allopregnanolone [pM]	138 (74.8, 234)	144 (99.5, 202)	92.1 (41.8, 176)
	Drug: F=0.4, p=0.69, η_p^2=0.0347; Age: F=5.2, p=0.033, η_p^2=0.198		
Allopregnanolone sulfate [nM]	8.23 (5.96, 11)	6.34 (5.09, 7.77)	4.04 (2.48, 6.07)
	Drug: F=1.9, p=0.175, η_p^2=0.153; Age: F=0.5, p=0.498, η_p^2=0.0221		
Isopregnanolone [pM]	115 (60.5, 200)	111 (74.7, 159)	40.5 (14.5, 88.1)
	Drug: F=1.5, p=0.237, η_p^2=0.128; Age: F=2.3, p=0.145, η_p^2=0.0984		
Isopregnanolone sulfate [nM]	23.6 (17.1, 33)	21.8 (17.7, 26.8)	14.7 (10.3, 20.9)
	Drug: F=1.2, p=0.327, η_p^2=0.101; Age: F=0, p=0.835, η_p^2=0.0021		
Pregnanolone [pM]	4.79 (1.74, 11.5)	19 (11.1, 32.8)	5.52 (1.82, 14.4)
	Drug: F=2.8, p=0.085, η_p^2=0.209; Age: F=0.4, p=0.562, η_p^2=0.0163		
Conjugated pregnanolone [nM]	44.8 (32.9, 62.2)†	28.9 (23.9, 35)	19.7 (14, 27.5)
	Drug: F=3.3, p=0.056, η_p^2=0.24; Age: F=0.2, p=0.636, η_p^2=0.0109		

Conjugated epipregnanolone [nM]	4.58 (3.39, 6.16)	4.1 (3.39, 4.95)	2.75 (1.91, 3.87)
	Drug: F=1.4, p=0.264, η_p^2=0.119; Age: F=0, p=0.894, η_p^2=0.000872		
17-Hydroxyallopregnanolone [pM]	53.3 (28, 92.5)	26.7 (16.4, 40.8)	15.1 (4.75, 34.8)
	Drug: F=1.5, p=0.24, η_p^2=0.127; Age: F=1, p=0.33, η_p^2=0.0453		
17-Hydroxyallopregnanolone sulfate [nM]	7.94 (6.05, 10.1)†	5.54 (4.51, 6.66)	3.79 (2.32, 5.55)
	Drug: F=2.6, p=0.097, η_p^2=0.199; Age: F=1.4, p=0.252, η_p^2=0.0619		
17-Hydroxypregnanolone [pM]	45.2 (17.9, 104)	64.4 (37.5, 108)	39.4 (13.5, 100)
	Drug: F=0.3, p=0.747, η_p^2=0.0274; Age: F=0.9, p=0.356, η_p^2=0.0406		
Conjugated 17-hydroxypregnanolone [nM]	26.8 (18.6, 39.4)†	18.7 (14.8, 23.5)	12.2 (8.03, 18.4)
	Drug: F=2.1, p=0.153, η_p^2=0.164; Age: F=0.1, p=0.766, η_p^2=0.00431		
5α,20α-Tetrahydroprogesterone [pM]	114 (77.1, 155)	103 (80.1, 128)‡	44.1 (16.8, 78.8)
	Drug: F=2.4, p=0.117, η_p^2=0.185; Age: F=1.4, p=0.256, η_p^2=0.0609		
Conjugated 5α,20α-tetrahydroprogesterone [pM]	82.1 (45.5, 133)	111 (83.9, 144)	102 (59.4, 161)
	Drug: F=0.3, p=0.735, η_p^2=0.0304; Age: F=0.7, p=0.403, η_p^2=0.0353		
**5α-Pregnane-3α,20α-diol [pM]**	**584 (400, 817)**, †††**	**226 (168, 295)**	**74 (29.3, 143)**
	**Drug: F=8.6, p=0.002, η_p^2=0.462; Age: F=1.5, p=0.242, η_p^2=0.0678**		
**Conjugated 5α-pregnane-3α,20α-diol [nM]**	**58.8 (44.9, 77.4)**, †††**	**27.1 (23.2, 31.5)‡‡**	**14 (10.1, 19)**
	**Drug: F=13.4, p<0.001, η_p^2=0.585; Age: F=0, p=0.871, η_p^2=0.00143**		
5α-Pregnane-3β,20α-diol [nM]	1.83 (1.07, 2.89)	1.54 (1.09, 2.1)	1.07 (0.517, 1.92)
	Drug: F=0.5, p=0.634, η_p^2=0.0424; Age: F=1.1, p=0.305, η_p^2=0.0501		
Conjugated 5α-pregnane-3β,20α-diol [μM]	3.6 (2.34, 5.49)	2.6 (1.96, 3.41)	2.02 (1.2, 3.29)
	Drug: F=0.8, p=0.454, η_p^2=0.0724; Age: F=1.6, p=0.225, η_p^2=0.0694		
Conjugated 5β,20α-tetrahydroprogesterone [pM]	96.6 (67.3, 138)**, †	246 (191, 321)	222 (146, 348)
	Drug: F=5, p=0.018, η_p^2=0.333; Age: F=3, p=0.097, η_p^2=0.132		
5β-Pregnane-3α,20α-diol [pM]	86.8 (37.9, 158)	115 (75.6, 164)	44.6 (10.1, 105)
	Drug: F=1.1, p=0.354, η_p^2=0.0941; Age: F=0, p=0.862, η_p^2=0.00147		
Conjugated 5β-pregnane-3α,20α-diol [nM]	14.4 (9.57, 22.4)	16.1 (12.3, 21.2)	8.77 (5.62, 13.8)
	Drug: F=1.4, p=0.268, η_p^2=0.118; Age: F=0.3, p=0.578, η_p^2=0.015		
5β-Pregnane-3β,20α-diol [pM]	183 (80.7, 411)	137 (80.4, 231)	63.5 (13.6, 228)
	Drug: F=0.5, p=0.62, η_p^2=0.0911; Age: F=1.7, p=0.22, η_p^2=0.146		
Conjugated 5β-pregnane-3β,20α-diol [nM]	7.71 (5.07, 11.5)	8.68 (6.65, 11.2)	7.94 (4.99, 12.3)
	Drug: F=0.1, p=0.923, η_p^2=0.00802; Age: F=2.7, p=0.118, η_p^2=0.117		
5α-Pregnane-3α,17,20α-triol [pM]	391 (233, 595)	378 (285, 485)	184 (82.1, 327)
	Drug: F=1.5, p=0.258, η_p^2=0.127; Age: F=4.2, p=0.053, η_p^2=0.175		
Conjugated 5α-pregnane-3α,17,20α-triol [nM]	94.9 (58.5, 157)	56.4 (41.6, 76.4)	52.9 (30.8, 90.6)
	Drug: F=1, p=0.386, ηp2=0.0867; Age: F=0.8, p=0.372, ηp2=0.0381		
5α-Pregnane-3β,17,20α-triol [pM]	453 (203, 999)	285 (170, 472)	144 (52.3, 357)
	Drug: F=0.9, p=0.425, η_p^2=0.0783; Age: F=1.9, p=0.184, η_p^2=0.0826		

Conjugated 5α-pregnane-3β,17,20α-triol [nM]	12.8 (5.82, 30.9)	3.92 (2.43, 6.34)	4.94 (1.95, 13)
	Drug: F=1.8, p=0.193, η_p^2=0.152; Age: F=0.8, p=0.395, η_p^2=0.0365		
5β-Pregnane-3α,17,20α-triol [nM]	2.32 (1.52, 3.39)	2.33 (1.79, 2.97)	1.29 (0.709, 2.11)
	Drug: F=1.3, p=0.306, η_p^2=0.107; Age: F=0.3, p=0.616, η_p^2=0.0122		
Conjugated 5β-pregnane-3α,17,20α-triol [nM]	254 (162, 386)	133 (97.1, 180)	104 (55.9, 179)
	Drug: F=2, p=0.163, η_p^2=0.159; Age: F=0.6, p=0.468, η_p^2=0.0253		
(f) **5α-/5β-reduced androstanes**			
5α-Androstane-3,17-dione [pM]	349 (248, 479)†	291 (233, 359)‡	149 (89.6, 231)
	Drug: F=2.7, p=0.094, η_p^2=0.202; Age: F=0.5, p=0.508, η_p^2=0.0211		
Androsterone [nM]	1.15 (0.924, 1.4)†	1.17 (1.02, 1.33)‡	0.683 (0.467, 0.918)
	Drug: F=3.3, p=0.058, η_p^2=0.248; Age: F=3.3, p=0.083, η_p^2=0.143		
Androsterone sulfate [μM]	5.17 (3.65, 7.07)	4.1 (3.24, 5.09)	3.1 (1.91, 4.66)
	Drug: F=0.9, p=0.421, η_p^2=0.0791; Age: F=2.5, p=0.13, η_p^2=0.106		
**Epiandrosterone [nM]**	**0.42 (0.304, 0.569)*,**	**0.694 (0.575, 0.834)‡‡‡**	**0.231 (0.152, 0.336)**
	**Drug: F=8.9, p=0.002, η_p^2=0.471; Age: F=5.8, p=0.026, η_p^2=0.225**		
Epiandrosterone sulfate [nM]	1160 (927, 1500)†	809 (708, 930)	668 (540, 838)
	Drug: F=3.3, p=0.06, η_p^2=0.246; Age: F=6.8, p=0.017, η_p^2=0.253		
Etiocholanolone [nM]	0.146 (0.102, 0.204)	0.242 (0.197, 0.295)	0.144 (0.096, 0.209)
	Drug: F=2.9, p=0.08, η_p^2=0.214; Age: F=0, p=0.912, η_p^2=0.000602		
Etiocholanolone sulfate [nM]	135 (93.1, 197)	149 (118, 189)	93.9 (61.3, 143)
	Drug: F=1, p=0.385, η_p^2=0.0869; Age: F=4.7, p=0.043, η_p^2=0.181		
Epietiocholanolone sulfate [nM]	39 (24.6, 64.4)	38.8 (28.9, 52.9)	28.5 (17.3, 48.4)
	Drug: F=0.3, p=0.745, η_p^2=0.0276; Age: F=2.3, p=0.146, η_p^2=0.0979		
5α-Dihydrotestosterone [nM]	1.21 (0.862, 1.6)	0.76 (0.575, 0.964)	0.839 (0.511, 1.22)
	Drug: F=1.4, p=0.277, η_p^2=0.115; Age: F=1.2, p=0.287, η_p^2=0.0538		
Conjugated 5α-dihydrotestosterone [nM]	4.36 (2.98, 6.27)	4.63 (3.62, 5.89)	3.21 (1.94, 5.12)
	Drug: F=0.5, p=0.604, η_p^2=0.0517; Age: F=4.8, p=0.041, η_p^2=0.202		
5α-Androstane-3α,17β-diol [nM]	0.299 (0.161, 0.483)	0.424 (0.311, 0.556)	0.292 (0.143, 0.498)
	Drug: F=0.6, p=0.541, η_p^2=0.0569; Age: F=3.1, p=0.091, η_p^2=0.13		
Conjugated 5α-androstane-3α,17β-diol [nM]	254 (155, 450)	169 (125, 230)	168 (100, 295)
	Drug: F=0.6, p=0.584, η_p^2=0.05; Age: F=4.9, p=0.039, η_p^2=0.188		
5α-Androstane-3β,17β-diol [pM]	201 (122, 320)†	102 (71.7, 141)	50.9 (23.9, 96.7)
	Drug: F=2.9, p=0.076, η_p^2=0.218; Age: F=0.4, p=0.529, η_p^2=0.0192		
Conjugated 5α-androstane-3β,17β-diol [nM]	254 (155, 450)	169 (125, 230)	168 (100, 295)
	Drug: F=0.6, p=0.584, ηp2=0.05; Age: F=4.9, p=0.039, ηp2=0.188		
**5β-Androstane-3α,17β-diol [pM]**	**27.4 (13.3, 63.7)**, †††**	**5.23 (3.62, 7.67)‡**	**1.65 (0.835, 3.11)**
	**Drug: F=9.2, p=0.001, η_p^2=0.468; Age: F=0.1, p=0.821, η_p^2=0.00249**		
Conjugated 5β-androstane-3α,17β-diol [nM]	15.9 (11.5, 22.6)	16.5 (13.4, 20.5)	15.3 (10.7, 22.6)
	Drug: F=0, p=0.966, η_p^2=0.00327; Age: F=0.9, p=0.347, η_p^2=0.0423		

Conjugated 5β-androstane-3β,17β-diol [nM]	0.644 (0.39, 1.02)	0.724 (0.534, 0.97)	0.765 (0.443, 1.27)
	Drug: F=0.1, p=0.936, η_p^2=0.00633; Age: F=0.2, p=0.657, η_p^2=0.00956		
	(g) **corticoids and 11β-hydroxy-androstanes**		
Cortisol [nM]	379 (324, 431)	432 (398, 465)	353 (291, 413)
	Drug: F=1.8, p=0.185, η_p^2=0.155; Age: F=0.6, p=0.457, η_p^2=0.028		
Cortisone [nM]	66.1 (46.9, 89.9)**	134 (111, 159)	112 (78.4, 157)
	Drug: F=4.4, p=0.027, η_p^2=0.317; Age: F=2.7, p=0.115, η_p^2=0.125		
Corticosterone [nM]	21 (15.9, 27.4)	22 (18.4, 26.2)	13.6 (9.58, 18.8)
	Drug: F=1.9, p=0.182, η_p^2=0.157; Age: F=1.5, p=0.24, η_p^2=0.0682		
21-Deoxycortisol [pM]	30.7 (14.2, 68.6)	45.7 (27.8, 77.2)	29.1 (12.3, 71.3)
	Drug: F=0.4, p=0.711, η_p^2=0.0319; Age: F=0, p=0.906, ηp2=0.000683		
11β-Hydroxyandrostenedione [nM]	33.4 (22.7, 46.6)	46 (37.4, 55.8)	35.3 (23.1, 50.8)
	Drug: F=0.9, p=0.405, η_p^2=0.0824; Age: F=0.4, p=0.529, η_p^2=0.0192		
11β-Hydroxyandrosterone [nM]	2.71 (1.81, 3.9)	3.06 (2.4, 3.83)	1.52 (0.872, 2.44)
	Drug: F=1.9, p=0.173, η_p^2=0.154; Age: F=2.9, p=0.106, η_p^2=0.12		
11β-Hydroxyandrosterone sulfate [nM]	53.3 (42.4, 67.7)†	50.2 (43.5, 58.3)‡	30.4 (23.9, 38.9)
	Drug: F=3.7, p=0.042, η_p^2=0.26; Age: F=0.1, p=0.814, η_p^2=0.00269		
11β-Hydroxyepiandrosterone [pM]	181 (129, 247)†	131 (102, 165)‡	58.2 (30.9, 96)
	Drug: F=3.8, p=0.039, η_p^2=0.276; Age: F=6.5, p=0.019, η_p^2=0.245		
11β-Hydroxyepiandrosterone sulfate [nM]	1.56 (0.987, 2.53)	0.918 (0.691, 1.22)	0.807 (0.485, 1.34)
	Drug: F=1.3, p=0.307, η_p^2=0.106; Age: F=0.2, p=0.64, η_p^2=0.0106		
11β-Hydroxyetiocholanolone [nM]	1.62 (1.11, 2.22)	1.68 (1.34, 2.05)	1.05 (0.588, 1.62)
	Drug: F=1, p=0.392, η_p^2=0.0853; Age: F=0, p=0.978, η_p^2=0.0000365		
11β-Hydroxyetiocholanolone sulfate [nM]	10.3 (6.35, 16.2)	7.7 (5.61, 10.4)	4.96 (2.56, 8.7)
	Drug: F=1, p=0.395, η_p^2=0.0847; Age: F=1.6, p=0.224, η_p^2=0.0697		
	(i) **CYP17A1, hydroxylase + lyase**		
DHEA/Preg	6.22 (4.55, 8.45)	8.45 (6.97, 10.2)	7.94 (5.64, 11.2)
	Drug: F=0.8, p=0.472, η_p^2=0.069; Age: F=0.2, p=0.68, η_p^2=0.00826		
DHEAC/Preg, C	18.9 (16.7, 21.4)	17.9 (16.4, 19.5)	21.6 (18.8, 24.8)
	Drug: F=1.4, p=0.262, η_p^2=0.131; Age: F=0.4, p=0.555, η_p^2=0.0186		
DHEA/20α-DHPreg	3.17 (2.43, 4.18)*	5.27 (4.36, 6.46)	4.55 (3.32, 6.46)
	Drug: F=2.5, p=0.11, η_p^2=0.19; Age: F=0.1, p=0.78, η_p^2=0.00379		
DHEA/20α-DHPreg, C	4.1 (3.29, 5.14)	4.26 (3.7, 4.92)	5.07 (3.94, 6.63)
	Drug: F=0.5, p=0.633, η_p^2=0.0426; Age: F=1.9, p=0.179, η_p^2=0.0844		
A/P	25.4 (8.79, 64.2)	40.3 (21.4, 72.9)	89.5 (29.3, 250)
	Drug: F=0.8, p=0.472, η_p^2=0.0759; Age: F=0.1, p=0.818, η_p^2=0.00287		
A/20α-DHP	17 (12.4, 24.6)	29.1 (22.5, 39.2)	35.1 (21.9, 67)
	Drug: F=1.9, p=0.178, η_p^2=0.152; Age: F=0.9, p=0.367, η_p^2=0.039		
5α-DHA/5α-DHP	8.14 (4.46, 16.5)	6.72 (4.62, 10.1)	8.62 (4.39, 19.5)
	Drug: F=0.2, p=0.865, η_p^2=0.0137; Age: F=0.2, p=0.646, η_p^2=0.0102		
5α-DHA/5α,20α-THP	3.08 (1.98, 5.09)	3 (2.27, 4.07)	3.91 (2.33, 7.32)
	Drug: F=0.2, p=0.82, η_p^2=0.0187; Age: F=0.8, p=0.397, η_p^2=0.0344		
3α,5α-THA/3α,5α-THP	8.18 (5.09, 14.1)	8.92 (6.54, 12.5)	8.26 (4.87, 15.3)

	Drug: F=0, p=0.969, η_p^2=0.00297; Age: F=3.3, p=0.082, η_p^2=0.137		
3α,5α-THA/3α,5α,20α-PD	2.66 (1.56, 4.7)	5 (3.44, 7.61)	7.52 (3.71, 20.3)
	Drug: F=1.4, p=0.259, η_p^2=0.121; Age: F=2.1, p=0.165, η_p^2=0.0896		
3α,5α-THA/3α,5α-THP, C	672 (448, 1030)	610 (472, 793)	683 (434, 1110)
	Drug: F=0.1, p=0.926, η_p^2=0.00728; Age: F=7, p=0.015, η_p^2=0.25		
3α,5α-THA/3α,5α,20α-PD, C	122 (81.5, 182)	140 (108, 180)	124 (79, 194)
	Drug: F=0.1, p=0.881, η_p^2=0.012; Age: F=3, p=0.099, η_p^2=0.124		
3β,5α-THA/3β,5α-THP	4.1 (2.78, 6.43)	5.37 (4.08, 7.31)	5.99 (3.69, 11.1)
	Drug: F=0.4, p=0.682, η_p^2=0.0358; Age: F=2.2, p=0.149, η_p^2=0.0966		
3β,5α-THA/3β,5α,20α-PD	0.26 (0.18, 0.391)	0.399 (0.306, 0.534)	0.212 (0.143, 0.327)
	Drug: F=2.1, p=0.149, η_p^2=0.166; Age: F=0.3, p=0.588, η_p^2=0.0142		
3β,5α-THA/3β,5α-THP, C	51.2 (37.3, 71.4)	33.5 (27.6, 40.8)	44.9 (31.7, 64.6)
	Drug: F=1.7, p=0.212, η_p^2=0.137; Age: F=2.2, p=0.156, η_p^2=0.0936		
3β,5α-THA/3β,5α,20α-PD, C	0.355 (0.242, 0.506)	0.307 (0.24, 0.388)	0.356 (0.231, 0.528)
	Drug: F=0.2, p=0.833, η_p^2=0.0173; Age: F=0.1, p=0.762, η_p^2=0.00445		
3α,5β-THA/3α,5β-THP	29.4 (14, 74.9)	11.8 (7.81, 18.2)	23.6 (10.8, 62.8)
	Drug: F=1.6, p=0.233, η_p^2=0.13; Age: F=0.8, p=0.391, η_p^2=0.0353		
3α,5β-THA/3α,5β,20α-PD	2.08 (1.26, 3.57)	1.86 (1.35, 2.62)	2.57 (1.38, 5.29)
	Drug: F=0.2, p=0.801, η_p^2=0.023; Age: F=0.1, p=0.755, η_p^2=0.00527		
3α,5β-THA/3α,5β-THP, C	2.86 (2.06, 4.02)	4.98 (3.97, 6.36)	4.34 (2.95, 6.63)
	Drug: F=2, p=0.164, η_p^2=0.158; Age: F=3, p=0.097, η_p^2=0.125		
3α,5β-THA/3α,5β,20α-PD, C	8.78 (6, 13.1)	8.86 (6.95, 11.4)	9.88 (6.43, 15.7)
	Drug: F=0.1, p=0.947, η_p^2=0.0052; Age: F=2, p=0.172, η_p^2=0.0871		
3β,5β-THA/3β,5β-THP, C	8.69 (5.47, 14)	9.95 (7.41, 13.5)	10.5 (6.26, 18.1)
	Drug: F=0.1, p=0.916, η_p^2=0.00828; Age: F=2.5, p=0.131, η_p^2=0.105		
3β,5β-THA/3β,5β,20α-PD, C	5.83 (3.86, 8.78)	4.24 (3.25, 5.5)	3.75 (2.32, 5.95)
	Drug: F=0.6, p=0.554, η_p^2=0.0548; Age: F=0.1, p=0.737, η_p^2=0.0055		
11β-OH-A/B	1.53 (1.08, 2.15)	2.4 (1.95, 2.97)	2.55 (1.76, 3.72)
	Drug: F=1.6, p=0.236, η_p^2=0.128; Age: F=0, p=0.926, η_p^2=0.000418		
(ii) **CYP17A1, hydroxylase only**			
17-OH-Preg/Preg	7.22 (5.35, 10.2)	8.07 (6.6, 10.1)	8.32 (5.86, 12.6)
	Drug: F=0.1, p=0.897, η_p^2=0.0103; Age: F=0.2, p=0.661, η_p^2=0.00934		
10^3·17-OH-Preg/Preg, C	91.4 (73.7, 111)	94.5 (82.8, 107)	75.3 (57.7, 95.5)
	Drug: F=0.7, p=0.488, η_p^2=0.066; Age: F=5.9, p=0.024, η_p^2=0.219		
17-OH-P/P	30.4 (14, 60.7)	21.9 (12.7, 36)	67.7 (29.4, 145)
	Drug: F=1.5, p=0.248, η_p^2=0.144; Age: F=0.1, p=0.788, η_p^2=0.00412		
17-OH-20α-DHP/20α-DHP	10.6 (7.41, 16.1)	10.7 (8.51, 13.9)	12.5 (8.19, 20.9)
	Drug: F=0.1, p=0.905, η_p^2=0.00943; Age: F=2.5, p=0.133, η_p^2=0.104		
17-OH-20α-DHP/20α-DHP, C	11.2 (7.21, 18.7)*	5.35 (4.2, 6.88)	5.18 (3.38, 8.13)
	Drug: F=2.5, p=0.105, η_p^2=0.193; Age: F=0.8, p=0.397, η_p^2=0.0345		
3α,5α,17-PD/3α,5α-THP	0.425 (0.196, 0.892)	0.168 (0.0985, 0.277)	0.251 (0.101, 0.587)
	Drug: F=1.2, p=0.337, η_p^2=0.0985; Age: F=0.7, p=0.405, η_p^2=0.0333		
3α,5α,17-PD/3α,5α-THP, C	1.02 (0.759, 1.36)	0.844 (0.696, 1.02)	0.881 (0.626, 1.22)
	Drug: F=0.3, p=0.725, η_p^2=0.0301; Age: F=0, p=0.844, η_p^2=0.00189		
3α,5β,17-PD/3α,5β-THP	8.73 (3.44, 26.4)	2.83 (1.64, 4.95)	4.7 (1.77, 14.2)

	Drug: F=1.2, p=0.312, η_p^2=0.105; Age: F=0.2, p=0.697, η_p^2=0.00738		
3α,5β,17-PD/3α,5β-THP, C	0.604 (0.456, 0.789)	0.654 (0.55, 0.773)	0.625 (0.457, 0.841)
	Drug: F=0.1, p=0.93, η_p^2=0.00689; Age: F=0, p=0.916, η_p^2=0.000543		
3α,5α,17,20α-PT/3α,5α,20α-PD	1.58 (0.814, 3.04)	1.56 (1.03, 2.36)	2.56 (1.24, 5.33)
	Drug: F=0.4, p=0.683, η_p^2=0.0356; Age: F=1.8, p=0.195, η_p^2=0.0788		
3α,5α,17,20α-PT/3α,5α,20α-PD, C	2.21 (1.18, 4.27)	2.06 (1.38, 3.09)	2.33 (1.16, 4.91)
	Drug: F=0, p=0.97, η_p^2=0.00287; Age: F=0.7, p=0.425, η_p^2=0.0306		
3β,5α,17,20α-PT/3β,5α,20α-PD	0.269 (0.106, 0.689)	0.225 (0.125, 0.405)	0.135 (0.0455, 0.383)
	Drug: F=0.3, p=0.77, η_p^2=0.0245; Age: F=0.1, p=0.728, η_p^2=0.00588		
10^3·3β,5α,17,20α-PT/3β,5α,20α-PD, C	3.99 (1.92, 8.65)	1.57 (0.989, 2.48)	2.33 (0.958, 5.82)
	Drug: F=1.3, p=0.292, η_p^2=0.116; Age: F=2.3, p=0.148, η_p^2=0.102		
3α,5β,17,20α-PT/3α,5β,20α-PD	29.2 (17.8, 53.4)	20.1 (14.8, 27.9)	32.2 (17.4, 72.7)
	Drug: F=0.8, p=0.487, η_p^2=0.0729; Age: F=0, p=0.924, η_p^2=0.000494		
3α,5β,17,20α-PT/3α,5β,20α-PD, C	15.9 (10.3, 25)*	7.77 (5.92, 10.2)	9.46 (5.86, 15.3)
	Drug: F=2.1, p=0.144, η_p^2=0.169; Age: F=2.3, p=0.142, η_p^2=0.0997		
F/B	17.7 (14.8, 21.1)†	19.6 (17.5, 22)	25.9 (21.3, 31.6)
	Drug: F=2.3, p=0.128, η_p^2=0.186; Age: F=1.6, p=0.225, η_p^2=0.0726		
	(iii) **CYP17A1, lyase only**		
DHEA/17-OH-Preg	0.82 (0.689, 0.974)	0.933 (0.834, 1.04)	0.887 (0.732, 1.07)
	Drug: F=0.4, p=0.653, η_p^2=0.0417; Age: F=2.3, p=0.142, η_p^2=0.105		
DHEA/17-OH-Preg, C	206 (159, 270)	181 (154, 214)‡	299 (219, 420)
	Drug: F=2.2, p=0.133, η_p^2=0.175; Age: F=2.9, p=0.102, η_p^2=0.122		
A/17-OH-P	0.963 (0.796, 1.19)*,	1.43 (1.23, 1.69)	1.11 (0.887, 1.44)
	Drug: F=2.9, p=0.08, η_p^2=0.214; Age: F=1.3, p=0.261, η_p^2=0.0597		
A/17-OH-20α-DHP	1.64 (1.21, 2.2)*, †	2.83 (2.34, 3.43)	3.11 (2.22, 4.42)
	Drug: F=3, p=0.073, η_p^2=0.221; Age: F=0.1, p=0.719, η_p^2=0.00628		
3α,5α-THA/3α,5α,17-PD	22.1 (12.6, 40)	44.3 (30, 69.1)	44.4 (22.7, 104)
	Drug: F=1.1, p=0.354, η_p^2=0.0942; Age: F=0.3, p=0.567, η_p^2=0.0158		
3α,5α-THA/3α,5α,17-PD, C	683 (455, 1030)	745 (576, 966)	814 (517, 1300)
	Drug: F=0.1, p=0.922, η_p^2=0.00774; Age: F=8, p=0.01, η_p^2=0.276		
3α,5β-THA/3α,5β,17-PD	3.42 (1.45, 8.36)	3.91 (2.28, 6.86)	4.38 (1.69, 12.4)
	Drug: F=0, p=0.963, η_p^2=0.00358; Age: F=1, p=0.322, η_p^2=0.0468		
3α,5β-THA/3α,5β,17-PD, C	5.22 (3.44, 7.51)	8.33 (6.7, 10.2)	8.29 (5.58, 11.9)
	Drug: F=1.4, p=0.274, ηp2=0.116; Age: F=3.9, p=0.061, ηp2=0.158		
3α,5α-THA/3α,5α,17,20α-PT	2.84 (1.88, 4.76)	3 (2.34, 3.98)	3.8 (2.38, 7.18)
	Drug: F=0.2, p=0.807, η_p^2=0.0213; Age: F=1.7, p=0.21, η_p^2=0.0775		
3α,5α-THA/3α,5α,17,20α-PT, C	54 (29.9, 97.6)	68.2 (47.1, 99.4)	53.3 (27.5, 103)
	Drug: F=0.2, p=0.82, η_p^2=0.0187; Age: F=3.7, p=0.067, η_p^2=0.151		
3β,5α-THA/3β,5α,17,20α-PT	0.998 (0.485, 2.12)	1.89 (1.17, 3.19)	1.44 (0.643, 3.59)
	Drug: F=0.6, p=0.566, η_p^2=0.0527; Age: F=1.1, p=0.305, η_p^2=0.0499		
3β,5α-THA/3β,5α,17,20α-PT, C	89.7 (43.3, 176)	193 (126, 294)	155 (66.8, 353)
	Drug: F=1, p=0.387, η_p^2=0.0906; Age: F=2.5, p=0.126, η_p^2=0.113		
10^3·3α,5β-THA/3α,5β,17,20α-PT	62.7 (40, 99.6)	103 (76.4, 141)	110 (65.3, 196)
	Drug: F=1, p=0.393, η_p^2=0.0852; Age: F=0.5, p=0.475, η_p^2=0.0245		
3α,5β-THA/3α,5β,17,20α-PT, C	0.513 (0.363, 0.729)*	1.13 (0.892, 1.43)	0.63 (0.412, 0.976)

	Drug: F=4.4, p=0.025, η_p^2=0.307; Age: F=5.9, p=0.024, η_p^2=0.229		
10^3·11β-OH-A/F	87.3 (66.5, 113)	118 (100, 138)	99.5 (74.2, 131)
	Drug: F=1.2, p=0.318, η_p^2=0.108; Age: F=0.4, p=0.558, η_p^2=0.0174		
10^{-3}·11β-OH-A/DOF	1.05 (0.484, 2.26)	0.891 (0.545, 1.44)	1.14 (0.48, 2.68)
	Drug: F=0.1, p=0.915, η_p^2=0.00843; Age: F=0.1, p=0.733, η_p^2=0.00565		
(iv) **HSD3B1, HSD3B2**			
10^3·P/Preg	79.8 (37.6, 201)	60 (37.1, 102)	38.9 (16.5, 101)
	Drug: F=0.4, p=0.695, η_p^2=0.0375; Age: F=0.1, p=0.733, η_p^2=0.00626		
10^3·P/PregC	0.281 (0.128, 0.719)	0.26 (0.155, 0.466)	0.115 (0.0472, 0.304)
	Drug: F=0.7, p=0.517, η_p^2=0.0671; Age: F=0.6, p=0.461, η_p^2=0.029		
10^3·20α-DHP/20α-DHPreg	58.9 (39.3, 83.8)	60.4 (47.2, 75.7)	44.6 (26.6, 68.5)
	Drug: F=0.4, p=0.682, η_p^2=0.0358; Age: F=0.8, p=0.383, η_p^2=0.0365		
10^6·20α-DHP/20α-DHPregC	84.9 (63.8, 114)	101 (83.3, 122)‡	51.6 (37.6, 70.7)
	Drug: F=3.6, p=0.048, η_p^2=0.262; Age: F=29.4, p<0.001, η_p^2=0.595		
10^3·20α-DHP/20α-DHPreg, C	0.689 (0.409, 1.13)	0.899 (0.657, 1.22)	0.808 (0.457, 1.39)
	Drug: F=0.2, p=0.796, η_p^2=0.0215; Age: F=4, p=0.059, η_p^2=0.16		
17-OH-P/17-OH-Preg	0.278 (0.203, 0.385)	0.212 (0.174, 0.258)	0.288 (0.202, 0.415)
	Drug: F=1, p=0.389, η_p^2=0.0861; Age: F=0.1, p=0.755, η_p^2=0.00476		
10^3·17-OH-P/17-OH-PregC	89.1 (60.5, 131)	83.7 (65.6, 107)	116 (75.4, 178)
	Drug: F=0.5, p=0.634, η_p^2=0.0425; Age: F=1.5, p=0.24, η_p^2=0.0652		
16α-OH-P/16α-OH-Preg	1.16 (0.917, 1.47)	1.03 (0.88, 1.2)	1.5 (1.16, 1.95)
	Drug: F=1.7, p=0.211, η_p^2=0.144; Age: F=0, p=0.951, η_p^2=0.000193		
A/DHEA	0.337 (0.249, 0.465)	0.319 (0.264, 0.389)	0.367 (0.261, 0.532)
	Drug: F=0.1, p=0.874, η_p^2=0.0128; Age: F=0.2, p=0.653, η_p^2=0.00981		
10^3·A/DHEAC	0.427 (0.299, 0.592)	0.706 (0.581, 0.853)	0.467 (0.316, 0.667)
	Drug: F=2.6, p=0.102, η_p^2=0.195; Age: F=3.8, p=0.066, η_p^2=0.152		
T/Adiol	4.59 (3.62, 5.9)	4.26 (3.67, 4.97)	5.47 (4.16, 7.34)
	Drug: F=0.7, p=0.517, η_p^2=0.0609; Age: F=1.4, p=0.257, η_p^2=0.0608		
10^3·T/AdiolC	5.88 (3.53, 9.68)	7.11 (5.18, 9.74)	6.69 (3.8, 11.7)
	Drug: F=0.1, p=0.893, η_p^2=0.0107; Age: F=8.7, p=0.008, η_p^2=0.294		
10^3·T/Adiol, C	5.62 (3.06, 10)	4.2 (2.84, 6.12)	5.49 (2.77, 10.5)
	Drug: F=0.3, p=0.771, η_p^2=0.0245; Age: F=5.9, p=0.025, η_p^2=0.218		
(v) **SULT2A1 (vs. STS)**			
Preg, C/U	274 (202, 379)	232 (191, 282)	291 (206, 419)
	Drug: F=0.5, p=0.626, η_p^2=0.0436; Age: F=9.8, p=0.005, η_p^2=0.317		
20α-DHPreg, C/U	648 (507, 831)	598 (512, 698)	776 (589, 1030)
	Drug: F=0.7, p=0.488, η_p^2=0.0661; Age: F=23.3, p<0.001, η_p^2=0.526		
17-OH-Preg, C/U	3.26 (2.51, 4.14)	2.65 (2.22, 3.12)	2.63 (1.91, 3.5)
	Drug: F=0.5, p=0.595, η_p^2=0.0482; Age: F=4.5, p=0.046, η_p^2=0.176		
DHEA, C/U	761 (564, 1070)*	466 (394, 554)	754 (542, 1110)
	Drug: F=3.4, p=0.053, η_p^2=0.244; Age: F=6.3, p=0.021, η_p^2=0.23		
Adiol, C/U	790 (502, 1220)	606 (450, 806)	648 (362, 1110)
	Drug: F=0.3, p=0.767, η_p^2=0.0262; Age: F=9.7, p=0.006, η_p^2=0.326		
3β,16α,17β-AT, C/U	984 (653, 1530)	611 (470, 796)	1230 (768, 2060)
	Drug: F=2.4, p=0.117, η_p^2=0.193; Age: F=4.4, p=0.048, η_p^2=0.182		

20α-DHP, C/U	7.5 (4.36, 13.5)	8.91 (6.26, 12.9)	13.6 (7.1, 29.3)
	Drug: F=0.5, p=0.623, η_p^2=0.0441; Age: F=0.7, p=0.416, η_p^2=0.0317		
17-OH-20α-DHP, C/U	7.17 (4.63, 12)	4.42 (3.43, 5.77)	5.39 (3.41, 9.03)
	Drug: F=1, p=0.386, η_p^2=0.0866; Age: F=0.4, p=0.56, η_p^2=0.0165		
T, C/U	0.869 (0.494, 1.58)	0.562 (0.393, 0.802)	0.782 (0.417, 1.51)
	Drug: F=0.6, p=0.569, η_p^2=0.0523; Age: F=0, p=0.964, η_p^2=0.0000985		
3α,5α-THP, C/U	61.7 (41.1, 97.6)	45.4 (35.5, 58.8)	52.7 (34, 85.9)
	Drug: F=0.5, p=0.637, η_p^2=0.0421; Age: F=8.6, p=0.008, η_p^2=0.291		
3β,5α-THP, C/U	218 (142, 349)	216 (164, 289)	382 (223, 752)
	Drug: F=1, p=0.398, η_p^2=0.0839; Age: F=6.2, p=0.021, η_p^2=0.228		
**10^{-3}·3α,5β-THP, C/U**	**9.04 (3.77, 26.9)****	**1.45 (0.932, 2.29)**	**3.62 (1.57, 9.62)**
	**Drug: F=4.4, p=0.026, η_p^2=0.294; Age: F=0.9, p=0.359, η_p^2=0.0401**		
3α,5α,17-PD, C/U	150 (90.5, 262)	219 (155, 322)	249 (135, 533)
	Drug: F=0.5, p=0.632, η_p^2=0.0427; Age: F=0.4, p=0.529, η_p^2=0.0192		
3α,5β,17-PD, C/U	613 (299, 1480)	310 (203, 484)	319 (151, 725)
	Drug: F=0.7, p=0.501, η_p^2=0.0637; Age: F=1.7, p=0.205, η_p^2=0.0755		
5α,20α-THP, C/U	0.677 (0.461, 1.01)††	1.16 (0.905, 1.53)‡	2.91 (1.66, 6.14)
	Drug: F=4.8, p=0.021, η_p^2=0.334; Age: F=0.2, p=0.646, η_p^2=0.0114		
3α,5α,20α-PD, C/U	123 (80.1, 190)	131 (99.8, 173)	214 (125, 376)
	Drug: F=0.8, p=0.464, η_p^2=0.074; Age: F=0.3, p=0.613, η_p^2=0.013		
10^{-3}·3β,5α,20α-PD, C/U	2.03 (1.43, 3)	2.17 (1.71, 2.8)	1.93 (1.31, 2.97)
	Drug: F=0.1, p=0.927, η_p^2=0.00756; Age: F=11.2, p=0.003, η_p^2=0.359		
3α,5β,20α-PD, C/U	220 (126, 405)	140 (98.1, 201)	193 (98.6, 407)
	Drug: F=0.6, p=0.563, η_p^2=0.0587; Age: F=0.4, p=0.517, η_p^2=0.0224		
3β,5β,20α-PD, C/U	47.3 (12.2, 163)	66.7 (29.4, 149)	152 (21.2, 1170)
	Drug: F=0.3, p=0.771, η_p^2=0.0508; Age: F=0.1, p=0.833, η_p^2=0.00469		
3α,5α,17,20α-PT, C/U	165 (90.7, 324)	153 (105, 230)	305 (146, 808)
	Drug: F=0.7, p=0.507, η_p^2=0.0627; Age: F=0.1, p=0.792, η_p^2=0.0034		
3β,5α,17,20α-PT, C/U	30.4 (18.7, 50.9)	14.6 (10.7, 19.8)‡	38.7 (21.1, 75.3)
	Drug: F=3.4, p=0.054, η_p^2=0.254; Age: F=1.2, p=0.297, η_p^2=0.0542		
3α,5β,17,20α-PT, C/U	108 (70.4, 165)	59.3 (44.9, 77.8)	80.2 (49.5, 129)
	Drug: F=1.6, p=0.22, η_p^2=0.134; Age: F=0.1, p=0.72, η_p^2=0.00627		
10^{-3}·3α,5α-THA, C/U	4.74 (3.31, 6.86)	3.83 (3.03, 4.84)	4.36 (2.94, 6.57)
	Drug: F=0.3, p=0.741, η_p^2=0.0296; Age: F=12.2, p=0.002, η_p^2=0.378		
**10^{-3}·3β,5α-THA, C/U**	**2.93 (2.16, 4.08)****	**1.28 (1.07, 1.53)‡‡**	**2.86 (2.06, 4.11)**
	**Drug: F=9.6, p=0.001, η_p^2=0.502; Age: F=17.5, p<0.001, η_p^2=0.48**		
10^{-3}·3α,5β-THA, C/U	0.902 (0.659, 1.29)	0.611 (0.511, 0.739)	0.621 (0.454, 0.877)
	Drug: F=1.3, p=0.304, η_p^2=0.107; Age: F=5.2, p=0.034, η_p^2=0.198		
5α-DHT, C/U	3.38 (2.35, 4.78)*, ††	6.23 (5.02, 7.7)‡	14.2 (8.9, 22.9)
	Drug: F=5.8, p=0.011, η_p^2=0.378; Age: F=5.3, p=0.033, η_p^2=0.218		
10^{-3}·3α,5α,17β-AD, C/U	0.889 (0.536, 1.7)	0.446 (0.339, 0.597)	0.59 (0.358, 1.07)
	Drug: F=1.6, p=0.227, η_p^2=0.132; Age: F=6.1, p=0.022, η_p^2=0.225		
10^{-3}·3β,5α,17β-AD, C/U	1.43 (0.922, 2.28)	2.03 (1.49, 2.82)	3.16 (1.81, 6.14)
	Drug: F=1.2, p=0.324, η_p^2=0.107; Age: F=10, p=0.005, η_p^2=0.333		
**10^{-3}·3α,5β,17β-AD, C/U**	**0.569 (0.264, 1.07)**, †††**	**3.11 (2.17, 4.48)‡**	**9.55 (4.9, 19.4)**

	**Drug: F=10.7, p<0.001, η_p^2=0.505; Age: F=0.1, p=0.832, η_p^2=0.00218**		
11β-OH-3α,5α-THA, C/U	19.6 (13.2, 31.1)	16 (12.6, 20.8)	19.2 (12.4, 32.3)
	Drug: F=0.3, p=0.772, η_p^2=0.0244; Age: F=2, p=0.174, η_p^2=0.086		
11β-OH-3β,5α-THA, C/U	8.68 (5.5, 14.6)	8.61 (6.43, 11.8)	15.4 (8.59, 32.9)
	Drug: F=0.8, p=0.448, η_p^2=0.0736; Age: F=1.7, p=0.201, η_p^2=0.0766		
11β-OH-3α,5β-THA, C/U	7.29 (4.02, 13)	4.96 (3.36, 7.2)	5.57 (2.79, 10.7)
	Drug: F=0.3, p=0.719, ηp2=0.0309; Age: F=1.1, p=0.31, ηp2=0.0489		
	(vi) **HSD11B1 (vs. HSD11B2)**		
7-oxo-DHEA/7α-OH-DHEA	0.127 (0.0698, 0.213)	0.22 (0.157, 0.303)	0.323 (0.181, 0.545)
	Drug: F=1.6, p=0.233, η_p^2=0.149; Age: F=0.6, p=0.469, η_p^2=0.0295		
7β-OH-DHEA/7α-OH-DHEA	0.647 (0.536, 0.771)	0.486 (0.426, 0.551)	0.601 (0.484, 0.734)
	Drug: F=2.1, p=0.146, η_p^2=0.168; Age: F=0.7, p=0.399, η_p^2=0.0341		
3β,7β,17β-AT/3β,7α,17β-AT	0.536 (0.46, 0.638)	0.438 (0.401, 0.481)	0.569 (0.476, 0.704)
	Drug: F=2.7, p=0.091, η_p^2=0.213; Age: F=0.1, p=0.804, η_p^2=0.00317		
F/E	5.66 (4.39, 7.28)*	3.29 (2.76, 3.91)	3.28 (2.34, 4.51)
	Drug: F=3.6, p=0.049, η_p^2=0.272; Age: F=2.4, p=0.139, η_p^2=0.112		
	(vii) **CYP11B1**		
11β-OH-A/A	8.94 (6.8, 11.7)	8.29 (6.97, 9.85)	7.61 (5.39, 10.6)
	Drug: F=0.1, p=0.866, η_p^2=0.0143; Age: F=0, p=0.895, η_p^2=0.000893		
11β-OH-3α,5α-THA/3α,5α-THA	2.65 (1.49, 4.19)	2.77 (1.98, 3.72)	2.61 (1.35, 4.34)
	Drug: F=0, p=0.987, η_p^2=0.00128; Age: F=0, p=0.835, η_p^2=0.00211		
10^3·11β-OH-3α,5α-THA/3α,5α-THA, C	10.3 (6.44, 16.8)	12.7 (9.35, 17.4)	10.7 (6.35, 18.6)
	Drug: F=0.2, p=0.828, η_p^2=0.0178; Age: F=2.4, p=0.137, η_p^2=0.102		
11β-OH-3β,5α-THA/3β,5α-THA	0.381 (0.24, 0.564)	0.209 (0.145, 0.287)	0.275 (0.15, 0.448)
	Drug: F=1.3, p=0.296, η_p^2=0.11; Age: F=2.2, p=0.155, η_p^2=0.0939		
10^3·11β-OH-3β,5α-THA/3β,5α-THA, C	1.35 (0.877, 2.05)	1.25 (0.952, 1.63)	1.26 (0.772, 2)
	Drug: F=0, p=0.974, η_p^2=0.00252; Age: F=3.4, p=0.081, η_p^2=0.138		
11β-OH-3α,5β-THA/3α,5β-THA	10.5 (6.63, 15.4)	7.25 (5.24, 9.67)	8.42 (4.77, 13.4)
	Drug: F=0.5, p=0.595, η_p^2=0.0482; Age: F=0, p=0.882, η_p^2=0.00108		
10^3·11β-OH-3α,5β-THA/3α,5β-THA, C	73.6 (45.3, 120)	48.3 (35.4, 65.6)	52.2 (30, 89.7)
	(viii) **CYP7B1, CYP3A4, CYP3A7**		
10^3·7α-OH-DHEA/DHEA	120 (92.9, 147)	113 (95.7, 130)	114 (84.2, 144)
	Drug: F=0.1, p=0.947, η_p^2=0.00518; Age: F=0.1, p=0.767, η_p^2=0.00426		
10^3·3β,7α,17β-AT/Adiol	196 (143, 264)	226 (187, 272)	205 (144, 284)
	Drug: F=0.2, p=0.817, η_p^2=0.0191; Age: F=0.5, p=0.505, η_p^2=0.0215		
10^3·7β-OH-DHEA/DHEA	74.9 (58.1, 96)*,	49.1 (41.5, 57.9)	59.5 (44.5, 78.9)
	Drug: F=2.2, p=0.135, η_p^2=0.173; Age: F=0.2, p=0.707, η_p^2=0.00686		
10^3·3β,7β,17β-AT/Adiol	106 (73.3, 149)	98.4 (77.7, 123)	121 (80.6, 174)
	Drug: F=0.2, p=0.797, η_p^2=0.0214; Age: F=0.1, p=0.726, η_p^2=0.00599		
10^3·16α-OH-Preg/Preg	354 (249, 505)	357 (283, 450)	368 (248, 549)
	Drug: F=0, p=0.994, η_p^2=0.000644; Age: F=0, p=0.916, η_p^2=0.000566		
10^3·3β,16α,17β-AT/Adiol	52.8 (37.7, 73)	66.3 (54, 81.1)	56.7 (39, 81.1)
	Drug: F=0.5, p=0.641, η_p^2=0.0415; Age: F=9.3, p=0.006, η_p^2=0.306		
10^3·3β,16α,17β-AT/Adiol, C	67.2 (43.2, 109)	68.9 (51.9, 93.1)	70.3 (42.9, 122)
	Drug: F=0, p=0.996, η_p^2=0.00041; Age: F=13.4, p=0.002, η_p^2=0.389		

16α-OH-P/P	5.3 (1.94, 12)	6.26 (3.43, 10.7)	16.1 (6.11, 36.7)
	Drug: F=1, p=0.405, η_p^2=0.0908; Age: F=0.3, p=0.596, η_p^2=0.0151		
	(ix) **SRD5A1, SRD5A2**		
(5α-DHP+3α/β,5α-THP)/P	2.08 (0.938, 4.33)	2.24 (1.35, 3.64)	2.14 (0.793, 5.24)
	Drug: F=0, p=0.992, η_p^2=0.000854; Age: F=1.4, p=0.248, η_p^2=0.0696		
3α/β,5α-THP, C/P	240 (96.9, 535)	242 (136, 413)	462 (166, 1160)
	Drug: F=0.4, p=0.69, η_p^2=0.0383; Age: F=0.1, p=0.792, η_p^2=0.00374		
(5α-DHP+3α/β,5α-THP, C+U)/P	242 (98.2, 540)	245 (137, 418)	466 (168, 1170)
	Drug: F=0.4, p=0.692, η_p^2=0.038; Age: F=0.1, p=0.799, η_p^2=0.00348		
(5α,20α-THP+3α/β,5α,20α-PD)/20α-DHP	13 (10.2, 16.6)	8.78 (7.48, 10.3)‡‡	19.1 (14.2, 25.9)
	Drug: F=6.7, p=0.007, η_p^2=0.412; Age: F=7.2, p=0.015, η_p^2=0.274		
10^{-3}·(5α,20α-THP+3α/β,5α,20α-PD)/20α-DHP, C	2.47 (1.47, 4.34)	1.61 (1.17, 2.23)	1.69 (0.957, 3.07)
	Drug: F=0.5, p=0.601, η_p^2=0.0474; Age: F=2.4, p=0.134, η_p^2=0.104		
10^{-3}·(5α,20α-THP+3α/β,5α,20α-PD)/20α-DHP, C+U	2.15 (1.34, 3.61)	1.39 (1.04, 1.87)	1.52 (0.912, 2.61)
	Drug: F=0.7, p=0.53, η_p^2=0.0587; Age: F=3.4, p=0.079, η_p^2=0.14		
10^{3}·3α,5α,17-PD/17-OH-P	13.9 (7.53, 24.7)	6.3 (4.09, 9.42)	4.26 (1.76, 8.9)
	Drug: F=1.8, p=0.196, η_p^2=0.144; Age: F=0.2, p=0.663, η_p^2=0.00921		
3α,5α,17-PDC/17-OH-P	2.18 (1.68, 2.76)†	1.51 (1.25, 1.8)	1.2 (0.817, 1.67)
	Drug: F=2.2, p=0.134, η_p^2=0.174; Age: F=0.4, p=0.555, η_p^2=0.0169		
3α,5α,17-PD, C+U/17-OH-P	2.2 (1.69, 2.79)†	1.52 (1.25, 1.81)	1.21 (0.821, 1.68)
	Drug: F=2.2, p=0.132, η_p^2=0.176; Age: F=0.4, p=0.554, η_p^2=0.0169		
3α/β,5α,17,20α-PT/17-OH-20α-DHP	0.446 (0.25, 0.82)	0.327 (0.227, 0.473)	0.228 (0.117, 0.435)
	Drug: F=0.6, p=0.561, η_p^2=0.0536; Age: F=1.9, p=0.187, η_p^2=0.0815		
3α/β,5α,17,20α-PT/17-OH-20α-DHP, C	6.87 (4.33, 11.3)	7.25 (5.38, 9.91)	11.8 (6.42, 24.3)
	Drug: F=0.6, p=0.555, η_p^2=0.0572; Age: F=0.7, p=0.405, η_p^2=0.0349		
3α/β,5α,17,20α-PT/17-OH-20α-DHP, C+U	6.03 (3.88, 9.71)	5.88 (4.44, 7.9)	10.3 (5.75, 20.8)
	Drug: F=0.8, p=0.459, η_p^2=0.0749; Age: F=0.6, p=0.456, η_p^2=0.028		
(5α-DHA+3α/β,5α-THA)/A	0.529 (0.392, 0.763)†	0.391 (0.33, 0.47)	0.279 (0.214, 0.373)
	Drug: F=2.3, p=0.122, η_p^2=0.182; Age: F=0.8, p=0.384, η_p^2=0.0364		
10^{-3}·(5α-DHA+3α/β,5α-THA, C+U)/A	1.84 (1.34, 2.57)**	0.874 (0.721, 1.06)	1.03 (0.736, 1.46)
	Drug: F=4.4, p=0.025, η_p^2=0.295; Age: F=10.2, p=0.004, η_p^2=0.328		
10^{3}·(5α-DHT+3α/β,5α,17β-AD)/T	104 (83.8, 129)	80.3 (70.1, 92)	67 (52.5, 85.2)
	Drug: F=1.9, p=0.169, η_p^2=0.156; Age: F=1.3, p=0.272, η_p^2=0.0571		
(5α-DHT+3α/β,5α,17β-AD)/T, C	40.4 (23.9, 68.2)	45.8 (33, 63.9)	33.8 (18.7, 60.5)
	Drug: F=0.2, p=0.789, η_p^2=0.0223; Age: F=5.4, p=0.031, η_p^2=0.203		
(5α-DHT+3α/β,5α,17β-AD)/T, C+U	24.9 (18.2, 34.1)	15.4 (12.8, 18.4)	13.6 (9.75, 18.7)
	Drug: F=2.3, p=0.125, η_p^2=0.188; Age: F=17.4, p<0.001, η_p^2=0.465		
10^{3}·11β-OH-3α/β,5α-THA/11β-OH-A	86.4 (67.2, 109)†	66.2 (55.4, 78.3)	46.2 (32.3, 63.3)
	Drug: F=2.5, p=0.112, η_p^2=0.197; Age: F=3.6, p=0.072, η_p^2=0.153		
11β-OH-3α/β,5α-THA, C/11β-OH-A	1.75 (1.19, 2.62)	1.01 (0.794, 1.3)	0.918 (0.605, 1.4)
	Drug: F=1.8, p=0.189, ηp2=0.153; Age: F=0.4, p=0.524, ηp2=0.0206		
11β-OH-3α/β,5α-THA, C+U/11β-OH-A	1.86 (1.29, 2.74)	1.09 (0.862, 1.39)	0.976 (0.649, 1.46)

	Drug: F=1.9, p=0.175, η_P^2=0.16; Age: F=0.4, p=0.563, η_P^2=0.017		
	(x) **AKR1D1**		
10^3·3α,5β-THP/P	31 (7.11, 96.6)	134 (65.8, 264)	140 (35.8, 492)
	Drug: F=1.4, p=0.277, η_P^2=0.127; Age: F=0.1, p=0.721, η_P^2=0.00689		
3α/β,5β-THP, C/P	374 (141, 893)	282 (147, 514)	585 (186, 1620)
	Drug: F=0.4, p=0.686, η_P^2=0.0389; Age: F=0, p=0.85, η_P^2=0.00193		
3α/β,5β-THP, C+U/P	374 (141, 893)	283 (147, 515)	585 (186, 1620)
	Drug: F=0.4, p=0.686, η_P^2=0.0388; Age: F=0, p=0.851, η_P^2=0.00192		
3α/β,5β,20α-PD/20α-DHP	1.25 (0.64, 3.12)	1.39 (0.872, 2.48)	1.38 (0.499, 9.04)
	Drug: F=0, p=0.983, η_P^2=0.00345; Age: F=1.2, p=0.309, η_P^2=0.103		
(5β,20α-THP+3α/β,5β-THP)/20α-DHP, C	16.8 (10.3, 25.9)	18.7 (14, 24.6)	17.2 (9.88, 27.7)
	Drug: F=0.1, p=0.944, η_P^2=0.00548; Age: F=2.5, p=0.132, η_P^2=0.105		
(5β,20α-THP+3α/β,5β-THP)/20α-DHP, C+U	20.1 (11.3, 34.9)	16.4 (11.3, 23.5)	13.1 (4.73, 31.2)
	Drug: F=0.2, p=0.829, η_P^2=0.0369; Age: F=3.4, p=0.093, η_P^2=0.256		
10^3·3α,5β,17-PD/17-OH-P	12.8 (5.6, 26.6)	17.7 (11, 27.9)	11.9 (4.63, 27)
	Drug: F=7.2, p=0.004, η_P^2=0.406; Age: F=0.6, p=0.434, η_P^2=0.0294		
3α,5β,17-PDC/17-OH-P	7.56 (5.67, 10.2)	5.37 (4.48, 6.43)	4.33 (3.14, 5.94)
	Drug: F=1.8, p=0.187, η_P^2=0.147; Age: F=6.3, p=0.02, η_P^2=0.231		
3α,5β,17-PD, C+U/17-OH-P	7.57 (5.68, 10.2)	5.39 (4.5, 6.46)	4.35 (3.17, 5.98)
	Drug: F=1.8, p=0.192, η_P^2=0.145; Age: F=6.2, p=0.021, η_P^2=0.229		
3α,5β,17,20α-PT/17-OH-20α-DHP	1.03 (0.759, 1.41)	1.17 (0.958, 1.42)	1.04 (0.735, 1.47)
	Drug: F=0.2, p=0.841, η_P^2=0.0163; Age: F=0.1, p=0.811, η_P^2=0.0028		
3α,5β,17,20α-PT/17-OH-20α-DHP, C	14 (9.45, 20.7)	15.2 (11.9, 19.5)	14.4 (9.28, 22.2)
	Drug: F=0, p=0.959, η_P^2=0.00397; Age: F=1.7, p=0.208, η_P^2=0.0744		
3α,5β,17,20α-PT/17-OH-20α-DHP, C+U	12.3 (8.43, 17.5)	12.5 (9.93, 15.7)	12.4 (8.17, 18.5)
	Drug: F=0, p=0.997, η_P^2=0.000292; Age: F=1.6, p=0.227, η_P^2=0.0688		
10^3·3α,5β-THA/A	40.4 (27.8, 59.3)	44.5 (35.1, 56.7)	40.5 (26.8, 62.2)
	Drug: F=0.1, p=0.927, η_P^2=0.00719; Age: F=1, p=0.336, η_P^2=0.0442		
3α,5β-THAC/A	51 (34.8, 76.3)	37 (29.2, 47.1)	37.4 (24.6, 57.6)
	Drug: F=0.6, p=0.579, η_P^2=0.0507; Age: F=7.9, p=0.01, η_P^2=0.274		
3α,5β-THA, C+U/A	51.1 (34.9, 76.3)	37.1 (29.2, 47.2)	37.5 (24.6, 57.7)
	Drug: F=0.6, p=0.58, η_P^2=0.0506; Age: F=7.9, p=0.011, η_P^2=0.273		
10^3·3α,5β,17β-AD/T	1.61 (0.764, 3.85)*, †††	0.348 (0.237, 0.521)‡	0.12 (0.0585, 0.235)
	Drug: F=7.2, p=0.004, η_P^2=0.406; Age: F=0.6, p=0.434, η_P^2=0.0294		
3α,5β,17β-AD/T, C	1.09 (0.641, 1.88)	1.92 (1.35, 2.8)	1.41 (0.778, 2.69)
	Drug: F=0.9, p=0.422, η_P^2=0.0789; Age: F=1.3, p=0.273, η_P^2=0.057		
3α,5β,17β-AD/T, C+U/A	0.504 (0.351, 0.729)	0.665 (0.527, 0.846)	0.581 (0.388, 0.885)
	Drug: F=0.5, p=0.629, η_P^2=0.0432; Age: F=2.2, p=0.153, η_P^2=0.0946		
10^3·11β-OH-3α,5β-THA/11β-OH-A	45.3 (35.8, 56.1)	36.2 (30.9, 42.1)	27.2 (19.4, 36.3)
	Drug: F=1.9, p=0.178, η_P^2=0.152; Age: F=0.7, p=0.421, η_P^2=0.0311		
10^3·11β-OH-3α,5β-THAC/11β-OH-A	306 (171, 558)	168 (116, 242)	144 (73.5, 274)
	Drug: F=1, p=0.386, η_P^2=0.0866; Age: F=1.9, p=0.183, η_P^2=0.083		
10^3·11β-OH-3α,5β-THA, C+U/11β-OH-A	366 (223, 624)	209 (154, 284)	176 (103, 303)
	Drug: F=1.3, p=0.307, η_P^2=0.107; Age: F=2.1, p=0.167, η_P^2=0.0889		

(xi) **AKR1C1 vs. HSD17B2**			
20α-DHPreg/Preg	1.9 (1.46, 2.48)	1.55 (1.31, 1.82)	1.65 (1.23, 2.21)
	Drug: F=0.5, p=0.622, η_P^2=0.0442; Age: F=0, p=0.871, η_P^2=0.00128		
20α-DHPreg/Preg, C	4.43 (3.88, 5.15)	3.89 (3.6, 4.23)	4.2 (3.65, 4.92)
	Drug: F=0.8, p=0.446, η_P^2=0.074; Age: F=3.3, p=0.083, η_P^2=0.136		
20α-DHP/P	1.38 (0.615, 2.82)	1.58 (0.951, 2.52)	2.55 (1.03, 5.68)
	Drug: F=0.3, p=0.726, η_P^2=0.0332; Age: F=0.4, p=0.541, η_P^2=0.02		
20α-DHPC/P	10.1 (3.19, 30)	12.3 (5.9, 25.1)	28.6 (7.44, 109)
	Drug: F=0.4, p=0.663, η_P^2=0.0424; Age: F=0, p=0.857, η_P^2=0.00175		
17-OH-20α-DHP/17-OH-P	0.587 (0.465, 0.757)	0.521 (0.451, 0.606)	0.371 (0.294, 0.474)
	Drug: F=2, p=0.157, η_P^2=0.161; Age: F=3.1, p=0.092, η_P^2=0.129		
17-OH-20α-DHPC/17-OH-P	4.68 (3.07, 7.22)	2.45 (1.88, 3.2)	2.13 (1.31, 3.39)
	Drug: F=2.1, p=0.143, η_P^2=0.169; Age: F=2.4, p=0.137, η_P^2=0.102		
5α,20α-THP/5α-DHP	3.07 (1.83, 5.39)	2.24 (1.62, 3.11)	1.85 (1.05, 3.29)
	Drug: F=0.5, p=0.636, η_P^2=0.0422; Age: F=2.1, p=0.16, η_P^2=0.0916		
5α,20α-THPC/5α-DHP	2.83 (1.35, 6.68)	2.26 (1.42, 3.71)	6.85 (2.64, 26)
	Drug: F=1.2, p=0.337, η_P^2=0.0984; Age: F=0.5, p=0.506, η_P^2=0.0214		
3α,5α,20α-PD/3α,5α-THP	2.5 (1.39, 5.12)	1.62 (1.14, 2.39)	1.54 (0.826, 3.3)
	Drug: F=0.4, p=0.65, η_P^2=0.0444; Age: F=8.3, p=0.009, η_P^2=0.305		
3α,5α,20α-PD/3α,5α-THP, C	5.53 (4.07, 7.75)	4.29 (3.57, 5.21)	5.54 (3.94, 8.1)
	Drug: F=0.8, p=0.459, η_P^2=0.0715; Age: F=1.8, p=0.198, η_P^2=0.0776		
3β,5α,20α-PD/3β,5α-THP	16.6 (9.43, 28.5)	14.9 (10.4, 21.1)	31 (17.1, 55.4)
	Drug: F=1.2, p=0.319, η_P^2=0.103; Age: F=0.5, p=0.481, η_P^2=0.0239		
3β,5α,20α-PD/3β,5α-THP, C	149 (109, 203)	117 (95, 142)	138 (96.5, 195)
	Drug: F=0.6, p=0.574, η_P^2=0.0515; Age: F=1.9, p=0.18, η_P^2=0.0839		
3α,5β,20α-PD/3α,5β-THP	16.2 (4.61, 116)	3.64 (1.96, 7.36)	2.47 (0.883, 8.28)
	Drug: F=1.5, p=0.256, η_P^2=0.122; Age: F=0.2, p=0.693, η_P^2=0.00758		
3α,5β,20α-PD/3α,5β-THP, C	0.339 (0.244, 0.461)*	0.583 (0.485, 0.696)	0.456 (0.323, 0.63)
	Drug: F=2.7, p=0.093, η_P^2=0.203; Age: F=0.1, p=0.745, η_P^2=0.00515		
3β,5β,20α-PD/3β,5β-THP, C	1.75 (1.07, 2.64)	2.57 (1.99, 3.25)	3.16 (2.05, 4.63)
	Drug: F=1.1, p=0.364, η_P^2=0.0918; Age: F=2.3, p=0.149, η_P^2=0.0967		
3α,5α,17,20α-PT/3α,5α,17-PD	10.2 (6.3, 17.7)	13.7 (9.82, 19.9)	9.65 (5.7, 17.8)
	Drug: F=0.4, p=0.647, η_P^2=0.0406; Age: F=0, p=0.874, η_P^2=0.00122		
3α,5α,17,20α-PT/3α,5α,17-PD, C	11.7 (6.9, 21.3)	10.5 (7.5, 15)	14 (7.62, 28.4)
	Drug: F=0.2, p=0.835, η_P^2=0.017; Age: F=0, p=0.839, η_P^2=0.002		
3α,5β,17,20α-PT/3α,5β,17-PD	47.1 (28, 86.4)	27.5 (20.2, 38.3)	36 (20.9, 67.4)
	Drug: F=0.9, p=0.43, η_P^2=0.081; Age: F=1.2, p=0.285, η_P^2=0.057		
3α,5β,17,20α-PT/3α,5β,17-PD, C	9.08 (6.74, 12.5)	6.85 (5.72, 8.26)	8.66 (6.06, 12.8)
	Drug: F=0.9, p=0.43, η_P^2=0.081; Age: F=1, p=0.325, η_P^2=0.0485		
(xii) **AKR1C2 vs. HSD17B2, HSD17B6**			
3α,5α-THP/3β,5α-THP	1.22 (0.685, 2.04)	1.46 (1.04, 2)	1.99 (1.12, 3.39)
	Drug: F=0.4, p=0.666, η_P^2=0.038; Age: F=0.6, p=0.464, η_P^2=0.0258		
10^3·3α,5α-THP/3β,5α-THP, C	328 (255, 414)	286 (242, 334)	261 (191, 344)
	Drug: F=0.4, p=0.678, η_P^2=0.0363; Age: F=3.1, p=0.091, η_P^2=0.13		
3α,5β-THP/3β,5β-THP, C	10.1 (7.83, 13)	7.2 (6.14, 8.44)	7.23 (5.44, 9.57)

	Drug: F=1.4, p=0.261, η_p^2=0.12; Age: F=0.9, p=0.363, η_p^2=0.0395		
10^3·3α,5α,20α-PD/3β,5α,20α-PD	194 (86.1, 472)	149 (89.7, 254)	62 (24.7, 151)
	Drug: F=1, p=0.382, η_p^2=0.0875; Age: F=1.2, p=0.297, η_p^2=0.0518		
10^3·3α,5α,20α-PD/3β,5α,20α-PD, C	11.9 (9.07, 15.7)	10.6 (8.97, 12.6)	10.8 (8, 14.7)
	Drug: F=0.1, p=0.878, η_p^2=0.0123; Age: F=2.8, p=0.112, η_p^2=0.116		
3α,5β,20α-PD/3β,5β,20α-PD	0.452 (0.0565, 1.41)	1.06 (0.494, 1.96)	2.03 (0.362, 6.47)
	Drug: F=0.7, p=0.529, η_p^2=0.12; Age: F=2, p=0.191, η_p^2=0.164		
3α,5β,20α-PD/3β,5β,20α-PD, C	2.11 (1.65, 2.73)†	1.69 (1.45, 1.97)	1.12 (0.856, 1.45)
	Drug: F=3.2, p=0.062, η_p^2=0.233; Age: F=1.6, p=0.219, η_p^2=0.071		
3α,5α,17,20α-PT/3β,5α,17,20α-PT	1.2 (0.816, 1.73)	1.25 (0.987, 1.58)	0.917 (0.583, 1.4)
	Drug: F=0.4, p=0.647, η_p^2=0.0406; Age: F=0.8, p=0.39, η_p^2=0.0354		
3α,5α,17,20α-PT/3β,5α,17,20α-PT, C	6.78 (3.13, 14.8)	12.8 (7.79, 21.9)	12.2 (4.73, 36.6)
	Drug: F=0.5, p=0.599, η_p^2=0.05; Age: F=0.1, p=0.819, η_p^2=0.00267		
3α,5α-THA/3β,5α-THA	2.58 (2.06, 3.25)*	1.78 (1.54, 2.06)‡	2.87 (2.24, 3.72)
	Drug: F=4.3, p=0.028, η_p^2=0.301; Age: F=1, p=0.34, η_p^2=0.0456		
3α,5α-THA/3β,5α-THA, C	4.27 (3.26, 5.6)	5.05 (4.26, 6)	4.01 (2.96, 5.42)
	Drug: F=0.7, p=0.533, η_p^2=0.0582; Age: F=0.6, p=0.465, η_p^2=0.0257		
3α,5β-THA/3β,5β-THA, C	3.69 (2.41, 5.33)	3.92 (3.04, 4.95)	3.29 (1.99, 5.03)
	Drug: F=0.1, p=0.878, η_p^2=0.0124; Age: F=0.1, p=0.719, η_p^2=0.00631		
3α,5α,17β-AD/3β,5α,17β-AD	4.25 (2.38, 9.22)	3.74 (2.78, 5.27)	4.62 (2.67, 9.45)
	Drug: F=0.1, p=0.884, η_p^2=0.0129; Age: F=0.2, p=0.68, η_p^2=0.00915		
3α,5α,17β-AD/3β,5α,17β-AD, C	1.04 (0.646, 1.57)	1.14 (0.859, 1.48)	1.04 (0.61, 1.65)
	Drug: F=0.1, p=0.95, η_p^2=0.00485; Age: F=2.1, p=0.162, η_p^2=0.0908		
3α,5β,17β-AD/3β,5β,17β-AD, C	25.7 (18.3, 37.3)	23.9 (19.3, 29.9)	20.7 (14.3, 30.7)
	Drug: F=0.2, p=0.836, η_p^2=0.0169; Age: F=0, p=0.877, η_p^2=0.00117		
11β-OH-3α,5α-THA/11β-OH-3β,5α-THA	14.7 (11.5, 18.9)**, †	27.7 (23, 33.9)	30 (21.6, 44.8)
	Drug: F=4.7, p=0.021, η_p^2=0.308; Age: F=0.7, p=0.404, η_p^2=0.0333		
11β-OH-3α,5α-THA/11β-OH-3β,5α-THA, C	33.3 (21, 52.7)	53.7 (40.1, 72.6)	37.2 (22.3, 62.5)
	Drug: F=1.1, p=0.365, η_p^2=0.0915; Age: F=0.1, p=0.742, η_p^2=0.00526		
3α,5α,17β-AD/5α-DHT	0.206 (0.102, 0.383)*	0.612 (0.427, 0.864)	0.414 (0.208, 0.776)
	Drug: F=2.6, p=0.097, η_p^2=0.2; Age: F=0.5, p=0.503, η_p^2=0.0216		
3α,5α,17β-AD/5α-DHT, C	55.8 (41.8, 76.8)	35.9 (30.3, 42.6)	40.3 (29.8, 55.5)
	Drug: F=1.9, p=0.174, η_p^2=0.154; Age: F=6.2, p=0.021, η_p^2=0.229		
3α,5α,17β-AD/5α-DHT, C+U	44.1 (33.8, 59.1)	30.8 (26.4, 36.2)	34.2 (25.9, 46.1)
	Drug: F=1.5, p=0.252, η_p^2=0.123; Age: F=7.1, p=0.014, η_p^2=0.254		
3α,5α-THP/5α-DHP	4.33 (2.39, 7.72)	3.3 (2.26, 4.78)	4.11 (2.1, 7.84)
	Drug: F=0.2, p=0.809, η_p^2=0.02; Age: F=6.6, p=0.018, η_p^2=0.238		
3α,5α-THPC/5α-DHP	232 (134, 427)	151 (108, 214)	253 (137, 514)
	Drug: F=0.9, p=0.439, η_p^2=0.0753; Age: F=0.2, p=0.66, η_p^2=0.00938		
3α,5α-THP, C+U/5α-DHP	237 (137, 435)	155 (111, 219)	259 (140, 525)
	Drug: F=0.9, p=0.441, η_p^2=0.075; Age: F=0.2, p=0.628, η_p^2=0.0114		
3α,5α,20α-PD/5α,20α-THP	4.64 (2.78, 9.39)*	2.03 (1.63, 2.57)	2.31 (1.51, 3.91)
	Drug: F=2.5, p=0.107, η_p^2=0.21; Age: F=5.9, p=0.025, η_p^2=0.238		
3α,5α-,20α-PD/5α,20α-THP, C	817 (419, 1870)*, †	258 (191, 356)	182 (112, 311)

	Drug: F=3.6, p=0.045, η_P^2=0.266; Age: F=2.2, p=0.152, η_P^2=0.0999		
3α,5α,20α-PD/5α,20α-THP, C+U	319 (199, 567)*,	131 (106, 163)	133 (92.7, 200)
	Drug: F=3.5, p=0.049, η_P^2=0.26; Age: F=4, p=0.058, η_P^2=0.168		
3α,5β,20α-PD/5β,20α-THP, C	157 (109, 231)*, ††	75.7 (60.5, 94.7)‡	40.2 (26.1, 60.2)
	Drug: F=6.5, p=0.007, η_P^2=0.381; Age: F=6.5, p=0.019, η_P^2=0.235		
3α,5α-THA/5α-DHA	3.24 (2.19, 4.9)	4.04 (3.13, 5.3)	4.5 (2.86, 7.48)
	Drug: F=0.3, p=0.726, η_P^2=0.03; Age: F=0.4, p=0.533, η_P^2=0.0188		
10^{-3}·3α,5α-THAC/5α-DHA	15.8 (10.6, 23.6)	13.5 (10.5, 17.3)	20 (12.8, 31.7)
	Drug: F=0.7, p=0.519, η_P^2=0.0605; Age: F=6.9, p=0.016, η_P^2=0.248		
10^{-3}·3α,5α-THA, C+U/5α-DHA	15.8 (10.6, 23.6)	13.5 (10.5, 17.3)	20 (12.8, 31.7)
	Drug: F=0.7, p=0.519, η_P^2=0.0606; Age: F=6.9, p=0.016, η_P^2=0.248		
(xiii) **HSD17B3, AKR1C3 vs. HSD17B2**			
Adiol/DHEA	0.347 (0.26, 0.47)*	0.215 (0.181, 0.256)	0.288 (0.211, 0.4)
	Drug: F=2.5, p=0.105, η_P^2=0.194; Age: F=0.5, p=0.481, η_P^2=0.024		
Adiol/DHEA, C	0.321 (0.23, 0.471)	0.265 (0.216, 0.33)	0.269 (0.188, 0.401)
	Drug: F=0.3, p=0.784, η_P^2=0.023; Age: F=4.5, p=0.046, η_P^2=0.177		
3β,7α,17β-AT/7α-OH-DHEA	0.603 (0.501, 0.719)	0.476 (0.42, 0.536)‡	0.73 (0.588, 0.897)
	Drug: F=3.8, p=0.04, η_P^2=0.276; Age: F=0.6, p=0.464, η_P^2=0.0271		
3β,7β,17β-AT/7β-OH-DHEA	0.492 (0.406, 0.602)	0.418 (0.37, 0.472)	0.567 (0.455, 0.715)
	Drug: F=1.8, p=0.185, η_P^2=0.148; Age: F=1.3, p=0.261, η_P^2=0.0597		
T/A	4.89 (3.84, 6.07)*	3.03 (2.49, 3.62)	4.43 (3.32, 5.69)
	Drug: F=3.4, p=0.052, η_P^2=0.245; Age: F=0.1, p=0.745, η_P^2=0.00514		
TC/A	4.46 (2.63, 7.25)	1.83 (1.22, 2.65)	3.67 (1.97, 6.39)
	Drug: F=2.6, p=0.101, η_P^2=0.197; Age: F=0, p=0.955, η_P^2=0.000158		
T, C+U/A	9.61 (7.15, 12.8)*	5.08 (4.11, 6.21)	8.65 (6.18, 11.9)
	Drug: F=4.5, p=0.024, η_P^2=0.3; Age: F=0, p=0.871, η_P^2=0.00129		
5α-DHT/5α-DHA	3.5 (2.59, 4.61)†	2.55 (2.07, 3.11)‡‡	6.49 (4.76, 8.67)
	Drug: F=6.8, p=0.006, η_P^2=0.403; Age: F=0, p=0.927, η_P^2=0.000436		
5α-DHTC/5α-DHA	13.6 (8.22, 22.6)	17.4 (12.7, 24.1)	31.7 (17.7, 60.9)
	Drug: F=1.3, p=0.304, η_P^2=0.107; Age: F=0.1, p=0.81, η_P^2=0.00282		
5α-DHT, C+U/5α-DHA	17.3 (10.8, 27.9)	20.4 (15.1, 27.6)	37 (21.4, 68.1)
	Drug: F=1.2, p=0.311, η_P^2=0.105; Age: F=0.1, p=0.822, η_P^2=0.00246		
3α,5α,17β-A/3α,5α-THA	0.377 (0.244, 0.515)	0.372 (0.287, 0.458)	0.416 (0.266, 0.571)
	Drug: F=0.1, p=0.933, η_P^2=0.00661; Age: F=0.8, p=0.395, η_P^2=0.0347		
10^{3}·3α,5α,17β-A/3α,5α-THA, C	48.4 (32.1, 75.5)	46.1 (35.5, 60.5)	60.8 (37.9, 104)
	Drug: F=0.3, p=0.766, η_P^2=0.0251; Age: F=0.8, p=0.383, η_P^2=0.0365		
10^{3}·3β,5α,17β-A/3β,5α-THA	437 (267, 680)*	186 (126, 263)	238 (123, 417)
	Drug: F=2.1, p=0.143, η_P^2=0.169; Age: F=0, p=0.902, η_P^2=0.000735		
10^{3}·3β,5α,17β-A/3β,5α-THA, C	230 (149, 353)	240 (183, 315)	275 (170, 443)
	Drug: F=0.1, p=0.92, η_P^2=0.00791; Age: F=1.7, p=0.204, η_P^2=0.0757		
**10^{3}·3α,5β,17β-AD/3α,5β-THA**	**194 (91.6, 467)***, †††**	**22 (15.4, 31.9)**	**12 (6.4, 22.4)**
	**Drug: F=10.5, p<0.001, η_P^2=0.501; Age: F=0, p=0.945, η_P^2=0.000229**		
10^{3}·3α,5β,17β-AD/3α,5β-THA, C	127 (91.8, 172)	115 (93.6, 140)	173 (123, 238)
	Drug: F=1.2, p=0.333, η_P^2=0.0995; Age: F=2.9, p=0.102, η_P^2=0.123		
10^{3}·3β,5β,17β-AD/3β,5β-THA, C	15.9 (11.6, 21.4)	15.2 (12.4, 18.5)	25.5 (18.4, 35.2)

Drug: $F=2$, $p=0.161$, $\eta_p^2=0.167$; Age: $F=2$, $p=0.177$, $\eta_p^2=0.0891$

Statistical significance of the treatment effect (Drug) was tested using an age-adjusted ANCOVA with $F(2, 21)$. Symbols indicate significant pairwise differences from the LSD post-hoc test between treatment groups: significantly higher values in clozapine vs. olanzapine (*$p < 0.05$, **$p < 0.01$, ***$p < 0.001$), in olanzapine vs. risperidone (‡$p < 0.05$, ‡‡$p < 0.01$, ‡‡‡$p < 0.001$), and in clozapine vs. risperidone (†$p < 0.05$, ††$p < 0.01$, †††$p < 0.001$).

To account for multiple testing, exact p-values for the Drug effect were recalculated from the reported F statistics [$F(2, 21)$] and subjected to Benjamini–Hochberg false discovery rate (BH-FDR) correction at $\alpha = 0.05$, applied separately within each predefined block reflecting the functional organization of the steroid metabolome. The native (unconjugated and conjugated) steroids were divided into seven biosynthetically homogeneous sub-blocks: (a) Δ^5 pregnanes; (b) Δ^5 androstanes; (c) Δ^4 pregnanes; (d) Δ^4 androstanes; (e) 5α-/5β-reduced pregnanes; (f) 5α-/5β-reduced androstanes; and (g) corticoids and 11β-hydroxy-androstanes. The enzyme-activity ratios were grouped into: (i) CYP17A1, hydroxylase + lyase; (ii) CYP17A1, hydroxylase only; (iii) CYP17A1, lyase only; (iv) HSD3B1/HSD3B2; (v) SULT2A1 (vs. STS); (vi) HSD11B1 (vs. HSD11B2) and CYP11B1; (vii) CYP7B1/CYP3A4/CYP3A7; (viii) SRD5A1/SRD5A2 and AKR1D1; (ix) AKR1C1 (vs. HSD17B2); (x) AKR1C2 (vs. HSD17B2/HSD17B6); and (xi) HSD17B3/AKR1C3 (vs. HSD17B2). **Variables shown in bold indicate a Drug effect that remained statistically significant after within-block BH-FDR correction.** After this correction, a significant Drug effect was retained for DHEA (Δ^5 androstanes), 5α-pregnane-3α,20α-diol and conjugated 5α-pregnane-3α,20α-diol (5α-/5β-reduced pregnanes), epiandrosterone and 5β-androstane-3α,17β-diol (5α-/5β-reduced androstanes), 10^{-3}·3α,5β,17β-AD,C/U and 10^{-3}·3β,5α-THA,C/U (SULT2A1), and 10^{3}·3α,5β,17β-AD/3α,5β-THA (HSD17B3/AKR1C3).

Abbreviations: C, conjugated (sulfated) form; U, unconjugated form; C/U, ratio of conjugated to unconjugated form; ηp^2, partial eta-squared (effect size). Steroid and metabolite abbreviations are as defined in the Materials and Methods section.
