# Supplementary material for: Steroidomic Differences Among Clozapine, Olanzapine, and Risperidone in Male Patients with First-Episode Psychosis, Including a Treatment-Resistant Schizophrenia Subgroup
Source: Int J Mol Sci. 2026 Oct 7;27(19):8909. doi: 10.3390/ijms27198909 (PMC13665165; doi:10.3390/ijms27198909)
Supplement: Supplementary file 1 [file ijms-27-08909-s001.zip › Supplementary Table S3 Baseline steroids.pdf]

Supplementary Table S3. Values of BMI, age and steroids on the beginning of the treatment (stage V1)

**Variable**	**Mean** (LCL95%, UCL95%)
	15 (11.2, 20.3)
BMI [kg/m^2]	25.2 (23.7, 27)
Age [years]	25.4 (23, 28.1)
(a) **Δ^5-pregnanes**	
Pregnenolone [nM]	1.39 (1.11, 1.73)
Pregnenolone sulfate [nM]	396 (321, 478)
20α-Dihydropregnenolone [nM]	2.76 (2.25, 3.37)
20α-Dihydropregnenolone sulfate [μM]	1.66 (1.32, 2.07)
17-Hydroxypregnenolone [nM]	14.6 (11, 18.9)
17-Hydroxypregnenolone sulfate [nM]	39.3 (30.4, 49.2)
16α-Hydroxypregnenolone [nM]	0.72 (0.587, 0.861)
(b) **Δ^5-androstanes**	
Dehydroepiandrosterone (DHEA) [nM]	12 (9.45, 15)
DHEA sulfate [μM]	8.12 (6.25, 10.3)
7α-Hydroxy-DHEA [nM]	1.56 (1.22, 1.93)
7-oxo-DHEA [nM]	0.374 (0.245, 0.568)
7β-Hydroxy-DHEA [nM]	0.79 (0.631, 0.965)
Androstenediol [nM]	3.55 (2.93, 4.34)
Androstenediol sulfate [μM]	1.96 (1.5, 2.5)
5-Androstene-3β,7α,17β-triol [nM]	0.817 (0.662, 1)
5-Androstene-3β,7β,17β-triol [nM]	0.435 (0.344, 0.543)
5-Androstene-3β,16α,17β-triol [nM]	0.227 (0.171, 0.302)
5-Androstene-3β,16α,17β-triol sulfate [nM]	149 (105, 215)
(c) **Δ^4-pregnanes**	
Progesterone [pM]	184 (104, 346)
20α-Dihydroprogesterone [pM]	194 (134, 271)
Conjugated 20α-dihydroprogesterone [nM]	1.61 (1.24, 2.07)
17-Hydroxyprogesterone [nM]	3.8 (3.31, 4.38)
16α-Hydroxyprogesterone [nM]	0.863 (0.688, 1.07)
17,20α-Dihydroxy-4-pregnene-3-one [nM]	1.63 (1.42, 1.89)
Conjugated 17,20α-dihydroxy-4-pregnene-3-one [nM]	8.91 (7.28, 11.2)
(d) **Δ^4-androstanes**	
Androstenedione [nM]	4.75 (4, 5.55)
Testosterone [nM]	18 (15.5, 20.8)
Conjugated testosterone [nM]	8.1 (6.1, 10.4)
(e) **5α-/5β-reduced pregnanes**	
5α-Dihydroprogesterone [pM]	36.9 (26.1, 53.1)
Allopregnanolone [pM]	147 (93, 225)
Allopregnanolone sulfate [nM]	7.01 (5.19, 9.3)
Isopregnanolone [pM]	110 (76.7, 148)
Isopregnanolone sulfate [nM]	19.6 (15.7, 24.4)
Pregnanolone [pM]	16 (9.01, 28)
Conjugated pregnanolone [nM]	28.4 (23.1, 35.6)
Conjugated epipregnanolone [nM]	3.86 (3.17, 4.7)
17-Hydroxyallopregnanolone [pM]	20.3 (13.3, 31.3)
17-Hydroxyallopregnanolone sulfate [nM]	4.49 (3.27, 6.15)
17-Hydroxypregnanolone [pM]	50.5 (28.8, 82.1)
Conjugated 17-hydroxypregnanolone [nM]	15.8 (12.3, 20.5)
5α,20α-Tetrahydroprogesterone [pM]	111 (79.3, 147)
Conjugated 5α,20α-tetrahydroprogesterone [pM]	133 (102, 179)
5α-Pregnane-3α,20α-diol [pM]	232 (164, 321)
Conjugated 5α-pregnane-3α,20α-diol [nM]	34.6 (24.8, 47)
5α-Pregnane-3β,20α-diol [nM]	1.17 (0.713, 1.89)
Conjugated 5α-pregnane-3β,20α-diol [μM]	2.99 (2.32, 3.71)

Conjugated 5β,20α-tetrahydroprogesterone [pM]	200 (251, 158)
5β-Pregnane-3α,20α-diol [pM]	104 (70.2, 150)
Conjugated 5β-pregnane-3α,20α-diol [nM]	15.3 (12.2, 19.2)
5β-Pregnane-3β,20α-diol [pM]	62 (42.6, 96.4)
Conjugated 5β-pregnane-3β,20α-diol [nM]	11.6 (9.4, 14.3)
5α-Pregnane-3α,17,20α-triol [pM]	308 (218, 447)
Conjugated 5α-pregnane-3α,17,20α-triol [nM]	52.3 (35.9, 75.2)
5α-Pregnane-3β,17,20α-triol [pM]	369 (238, 556)
Conjugated 5α-pregnane-3β,17,20α-triol [nM]	7.94 (5.26, 12.1)
5β-Pregnane-3α,17,20α-triol [nM]	2.13 (1.67, 2.62)
Conjugated 5β-pregnane-3α,17,20α-triol [nM]	137 (105, 178)
(f) **5α-/5β-reduced androstanes**	
5α-Androstane-3,17-dione [pM]	352 (262, 460)
Androsterone [nM]	1.06 (0.897, 1.25)
Androsterone sulfate [μM]	4.46 (3, 6.5)
Epiandrosterone [nM]	0.466 (0.358, 0.598)
Epiandrosterone sulfate [nM]	920 (698, 1170)
Etiocholanolone [nM]	0.188 (0.157, 0.227)
Etiocholanolone sulfate [nM]	113 (86.8, 149)
Epietiocholanolone sulfate [nM]	40.6 (29.6, 55.1)
5α-Dihydrotestosterone [nM]	0.945 (0.733, 1.18)
Conjugated 5α-dihydrotestosterone [nM]	5.16 (3.67, 7.32)
5α-Androstane-3α,17β-diol [nM]	0.448 (0.33, 0.575)
Conjugated 5α-androstane-3α,17β-diol [nM]	192 (142, 258)
5α-Androstane-3β,17β-diol [pM]	122 (76, 193)
Conjugated 5α-androstane-3β,17β-diol [nM]	201 (151, 261)
5β-Androstane-3α,17β-diol [pM]	9.57 (4.78, 18.7)
Conjugated 5β-androstane-3α,17β-diol [nM]	15 (11.9, 18.6)
Conjugated 5β-androstane-3β,17β-diol [nM]	0.626 (0.48, 0.81)
(g) **corticoids and 11β-hydroxy-androstanes**	
Cortisol [nM]	360 (320, 401)
Cortisone [nM]	92.9 (76.8, 114)
Corticosterone [nM]	23 (17.1, 29.5)
21-Deoxycortisol [pM]	34.3 (17.7, 65.3)
11β-Hydroxyandrostenedione [nM]	36.3 (28.4, 45.3)
11β-Hydroxyandrosterone [nM]	1.85 (1.34, 2.48)
11β-Hydroxyandrosterone sulfate [nM]	37.4 (31.4, 44.3)
11β-Hydroxyepiandrosterone [pM]	70.2 (47.4, 101)
11β-Hydroxyepiandrosterone sulfate [nM]	1.22 (1.56, 0.917)
11β-Hydroxyetiocholanolone [nM]	1.2 (0.864, 1.61)
11β-Hydroxyetiocholanolone sulfate [nM]	9.22 (6.85, 12.3)
(i) **CYP17A1, hydroxylase + lyase**	
DHEA/Preg	8.28 (6.94, 9.96)
DHEA/Preg, C	20.2 (16.7, 24.3)
DHEA/20α-DHPreg	4.35 (3.56, 5.3)
DHEA/20α-DHPreg, C	4.46 (3.79, 5.29)
A/P	26.7 (14.6, 46)
A/20α-DHP	24.7 (17.4, 35.8)
5α-DHA/5α-DHP	8.22 (5.76, 11.6)
5α-DHA/5α,20α-THP	3.29 (2.44, 4.57)
3α,5α-THA/3α,5α-THP	7.6 (5.31, 11.1)
3α,5α-THA/3α,5α,20α-PD	4.8 (3.64, 6.36)
3α,5α-THA/3α,5α-THP, C	627 (447, 877)
3α,5α-THA/3α,5α,20α-PD, C	123 (90.4, 170)
3β,5α-THA/3β,5α-THP	4.71 (3.88, 5.83)
3β,5α-THA/3β,5α,20α-PD	0.397 (0.295, 0.507)
3β,5α-THA/3β,5α-THP, C	41.7 (31.2, 55.4)
3β,5α-THA/3β,5α,20α-PD, C	0.296 (0.225, 0.392)
3α,5β-THA/3α,5β-THP	11.4 (7.18, 18.7)
3α,5β-THA/3α,5β,20α-PD	1.83 (1.39, 2.46)
3α,5β-THA/3α,5β-THP, C	3.74 (2.84, 4.99)

3α,5β-THA/3α,5β,20α-PD, C	7.5 (5.74, 9.93)
3β,5β-THA/3β,5β-THP, C	10.6 (7.85, 13.8)
3β,5β-THA/3β,5β,20α-PD, C	3.75 (2.82, 4.9)
11β-OH-A/B	1.71 (1.39, 2.13)
17-OH-Preg/Preg	10.2 (12.7, 8.09)
10^3·17-OH-Preg/Preg, C	102 (88.5, 116)
(ii) **CYP17A1, hydroxylase only**	
17-OH-P/P	22.4 (12.6, 37.2)
17-OH-20α-DHP/20α-DHP	9.79 (6.95, 13.8)
17-OH-20α-DHP/20α-DHP, C	6.29 (4.97, 7.83)
3α,5α,17-PD/3α,5α-THP	0.193 (0.125, 0.283)
3α,5α,17-PD/3α,5α-THP, C	0.694 (0.607, 0.774)
3α,5β,17-PD/3α,5β-THP	2.55 (1.29, 5.17)
3α,5β,17-PD/3α,5β-THP, C	0.548 (0.478, 0.631)
3α,5α,17,20α-PT/3α,5α,20α-PD	1.46 (0.98, 2.17)
3α,5α,17,20α-PT/3α,5α,20α-PD, C	1.37 (0.876, 2.2)
3β,5α,17,20α-PT/3β,5α,20α-PD	0.336 (0.19, 0.589)
10^3·3β,5α,17,20α-PT/3β,5α,20α-PD, C	2.73 (1.84, 4.17)
3α,5β,17,20α-PT/3α,5β,20α-PD	22.8 (17, 31.8)
3α,5β,17,20α-PT/3α,5β,20α-PD, C	9.76 (7.62, 12.2)
F/B	15.2 (12.9, 18.2)
(iii) **CYP17A1, lyase only**	
DHEA/17-OH-Preg	0.88 (0.749, 1.03)
DHEA/17-OH-Preg, C	193 (159, 239)
A/17-OH-P	1.16 (0.995, 1.35)
A/17-OH-20α-DHP	2.87 (2.38, 3.39)
3α,5α-THA/3α,5α,17-PD	53.1 (36.4, 76.8)
3α,5α-THA/3α,5α,17-PD, C	1010 (662, 1490)
3α,5β-THA/3α,5β,17-PD	4.46 (2.73, 7.58)
3α,5β-THA/3α,5β,17-PD, C	7.44 (5.45, 10.1)
3α,5α-THA/3α,5α,17,20α-PT	3.61 (2.48, 5.08)
3α,5α-THA/3α,5α,17,20α-PT, C	111 (63.6, 180)
3β,5α-THA/3β,5α,17,20α-PT	1.27 (0.854, 1.91)
3β,5α-THA/3β,5α,17,20α-PT, C	112 (69.2, 177)
10^3·3α,5β-THA/3α,5β,17,20α-PT	91.9 (73.7, 117)
3α,5β-THA/3α,5β,17,20α-PT, C	0.786 (0.579, 1.09)
10^3·11β-OH-A/F	104 (87, 123)
10^{-3}·11β-OH-A/DOF	0.888 (0.534, 1.55)
(iv) **HSD3B1, HSD3B2**	
10^3·P/Preg	119 (75.3, 198)
10^3·P/PregC	0.392 (0.24, 0.683)
10^3·20α-DHP/20α-DHPreg	64.5 (50, 83.2)
10^6·20α-DHP/20α-DHPregC	106 (79.2, 142)
10^3·20α-DHP/20α-DHPreg, C	0.952 (0.714, 1.28)
17-OH-P/17-OH-Preg	0.253 (0.204, 0.318)
10^3·17-OH-P/17-OH-PregC	104 (80.7, 134)
16α-OH-P/16α-OH-Preg	1.33 (1.08, 1.6)
A/DHEA	0.374 (0.32, 0.437)
10^3·A/DHEAC	0.595 (0.472, 0.754)
T/Adiol	4.76 (4.24, 5.37)
10^3·T/AdiolC	9.18 (6.99, 12.2)
10^3·T/Adiol, C	9.32 (7.09, 12.4)
(v) **SULT2A1 (vs. STS)**	
Preg, C/U	262 (224, 312)
20α-DHPreg, C/U	602 (509, 712)
17-OH-Preg, C/U	2.62 (2.15, 3.23)
DHEA, C/U	632 (514, 780)
Adiol, C/U	550 (398, 749)
3β,16α,17β-AT, C/U	764 (578, 968)
20α-DHP, C/U	8.89 (5.77, 13.8)

17-OH-20α-DHP, C/U	5.17 (4.24, 6.47)
T, C/U	0.415 (0.306, 0.563)
3α,5α-THP, C/U	50.8 (34.7, 73.6)
3β,5α-THP, C/U	195 (145, 270)
10^{-3}·3α,5β-THP, C/U	1.96 (1.14, 3.31)
3α,5α,17-PD, C/U	206 (155, 277)
3α,5β,17-PD, C/U	351 (239, 535)
5α,20α-THP, C/U	1.47 (0.999, 2.23)
3α,5α,20α-PD, C/U	122 (82.2, 197)
10^{-3}·3β,5α,20α-PD, C/U	2.33 (1.43, 3.81)
3α,5β,20α-PD, C/U	159 (105, 236)
3β,5β,20α-PD, C/U	198 (116, 307)
3α,5α,17,20α-PT, C/U	173 (108, 276)
3β,5α,17,20α-PT, C/U	22.6 (16.4, 30.9)
3α,5β,17,20α-PT, C/U	68.6 (52.1, 91.6)
10^{-3}·3α,5α-THA, C/U	4.44 (3.35, 5.57)
10^{-3}·3β,5α-THA, C/U	1.78 (1.33, 2.38)
10^{-3}·3α,5β-THA, C/U	0.621 (0.499, 0.773)
5α-DHT, C/U	6.55 (4.8, 8.84)
10^{-3}·3α,5α,17β-AD, C/U	0.484 (0.331, 0.743)
10^{-3}·3β,5α,17β-AD, C/U	1.7 (1.04, 2.69)
10^{-3}·3α,5β,17β-AD, C/U	1.63 (0.848, 3.34)
11β-OH-3α,5α-THA, C/U	22.1 (16.8, 29.2)
11β-OH-3β,5α-THA, C/U	15.7 (11.4, 22.2)
11β-OH-3α,5β-THA, C/U	8.52 (6.35, 11.1)
(vi) **HSD11B1 (vs. HSD11B2)**	
7-oxo-DHEA/7α-OH-DHEA	0.232 (0.166, 0.327)
7β-OH-DHEA/7α-OH-DHEA	0.524 (0.454, 0.605)
3β,7β,17β-AT/3β,7α,17β-AT	0.532 (0.461, 0.613)
F/E	3.92 (3.13, 4.78)
(vii) **CYP11B1**	
11β-OH-A/A	7.82 (6.27, 9.6)
11β-OH-3α,5α-THA/3α,5α-THA	1.61 (1.17, 2.19)
10^{3}·11β-OH-3α,5α-THA/3α,5α-THA, C	7.84 (6.51, 9.85)
11β-OH-3β,5α-THA/3β,5α-THA	0.154 (0.1, 0.234)
10^{3}·11β-OH-3β,5α-THA/3β,5α-THA, C	1.22 (0.867, 1.76)
11β-OH-3α,5β-THA/3α,5β-THA	6.5 (4.64, 8.76)
10^{3}·11β-OH-3α,5β-THA/3α,5β-THA, C	78.9 (56.7, 109)
(viii) **CYP7B1, CYP3A4, CYP3A7**	
10^{3}·7α-OH-DHEA/DHEA	119 (100, 138)
10^{3}·3β,7α,17β-AT/Adiol	214 (178, 259)
10^{3}·7β-OH-DHEA/DHEA	62.5 (51.3, 75.7)
10^{3}·3β,7β,17β-AT/Adiol	113 (93.5, 138)
10^{3}·16α-OH-Preg/Preg	477 (391, 585)
10^{3}·3β,16α,17β-AT/Adiol	52.8 (37.7, 73)
10^{3}·3β,16α,17β-AT/Adiol, C	69.4 (47.5, 106)
16α-OH-P/P	5.87 (3.53, 8.91)
(ix) **SRD5A1, SRD5A2**	
(5α-DHP+3α/β,5α-THP)/P	1.79 (1.02, 2.99)
3α/β,5α-THP, C/P	191 (104, 326)
(5α-DHP+3α/β,5α-THP, C+U)/P	193 (105, 330)
(5α,20α-THP+3α/β,5α,20α-PD)/20α-DHP	7.66 (5.75, 10.1)
10^{-3}·(5α,20α-THP+3α/β,5α,20α-PD)/20α-DHP, C	1.74 (1.25, 2.4)
10^{-3}·(5α,20α-THP+3α/β,5α,20α-PD)/20α-DHP, C+U	1.54 (1.15, 2.01)
10^{3}·3α,5α,17-PD/17-OH-P	6.27 (4.22, 9.08)
3α,5α,17-PDC/17-OH-P	1.27 (0.956, 1.68)
3α,5α,17-PD, C+U/17-OH-P	1.28 (0.961, 1.69)
3α/β,5α,17,20α-PT/17-OH-20α-DHP	0.408 (0.276, 0.612)
3α/β,5α,17,20α-PT/17-OH-20α-DHP, C	6.06 (4.44, 8.44)

3α/β,5α,17,20α-PT/17-OH-20α-DHP, C+U	5.17 (3.86, 7.09)
(5α-DHA+3α/β,5α-THA)/A	0.423 (0.349, 0.511)
10^{-3}·(5α-DHA+3α/β,5α-THA, C+U)/A	1.24 (0.88, 1.69)
10^{3}·(5α-DHT+3α/β,5α,17β-AD)/T	85.4 (71.1, 102)
(5α-DHT+3α/β,5α,17β-AD)/T, C	61.2 (43.7, 83.2)
(5α-DHT+3α/β,5α,17β-AD)/T, C+U	17.7 (13.8, 21.9)
10^{3}·11β-OH-3α/β,5α-THA/11β-OH-A	55 (41.8, 71.1)
11β-OH-3α/β,5α-THA, C/11β-OH-A	1.12 (1.48, 0.852)
11β-OH-3α/β,5α-THA, C+U/11β-OH-A	1.14 (0.887, 1.47)
(x) **AKR1D1**	
10^{3}·3α,5β-THP/P	72.9 (38.4, 134)
3α/β,5β-THP, C/P	241 (130, 420)
3α/β,5β-THP, C+U/P	241 (130, 420)
3α/β,5β,20α-PD/20α-DHP	0.743 (0.46, 1.27)
(5β,20α-THP+3α/β,5β-THP)/20α-DHP, C	16.2 (20, 13.3)
(5β,20α-THP+3α/β,5β-THP)/20α-DHP, C+U	16.3 (12.4, 21.1)
10^{3}·3α,5β,17-PD/17-OH-P	12.8 (7.63, 20.3)
3α,5β,17-PDC/17-OH-P	4.44 (3.66, 5.36)
3α,5β,17-PD, C+U/17-OH-P	4.46 (3.67, 5.39)
3α,5β,17,20α-PT/17-OH-20α-DHP	1.27 (1.01, 1.55)
3α,5β,17,20α-PT/17-OH-20α-DHP, C	15.4 (11.4, 20)
3α,5β,17,20α-PT/17-OH-20α-DHP, C+U	12.3 (7.05, 19.5)
10^{3}·3α,5β-THA/A	40 (47.6, 33.9)
3α,5β-THAC/A	35.3 (42.4, 30.2)
3α,5β-THA, C+U/A	37.7 (45.8, 31.2)
10^{3}·3α,5β,17β-AD/T	0.495 (0.247, 0.992)
3α,5β,17β-AD/T, C	2.09 (1.53, 2.85)
3α,5β,17β-AD/T, C+U/A	572 (727, 451)
10^{3}·11β-OH-3α,5β-THA/11β-OH-A	33.2 (26.4, 41.1)
10^{3}·11β-OH-3α,5β-THAC/11β-OH-A	261 (366, 188)
10^{3}·11β-OH-3α,5β-THA, C+U/11β-OH-A	305 (416, 224)
(xi) **AKR1C1 vs. HSD17B2**	
20α-DHPreg/Preg	1.92 (1.59, 2.34)
20α-DHPreg/Preg, C	4.48 (5.02, 3.98)
20α-DHP/P	1.08 (0.593, 1.86)
20α-DHPC/P	10.1 (5.18, 19.2)
17-OH-20α-DHP/17-OH-P	0.444 (0.524, 0.38)
17-OH-20α-DHPC/17-OH-P	2.5 (1.96, 3.27)
5α,20α-THP/5α-DHP	2.5 (1.73, 3.5)
3α,5α,20α-PD/3α,5α-THP	3.16 (2.23, 4.63)
3α,5α,20α-PD/3α,5α-THP, C	4.83 (4.16, 5.63)
3β,5α,20α-PD/3β,5α-THP	13.1 (9.61, 18.6)
3β,5α,20α-PD/3β,5α-THP, C	137 (112, 166)
3α,5β,20α-PD/3α,5β-THP	6.31 (4.14, 9.81)
3α,5β,20α-PD/3α,5β-THP, C	0.496 (0.425, 0.583)
3β,5β,20α-PD/3β,5β-THP, C	2.89 (2.46, 3.44)
3α,5α,17,20α-PT/3α,5α,17-PD	13.8 (9.94, 19.8)
3α,5α,17,20α-PT/3α,5α,17-PD, C	9.93 (6.74, 15.1)
3α,5β,17,20α-PT/3α,5β,17-PD	45.5 (30.4, 70.1)
3α,5β,17,20α-PT/3α,5β,17-PD, C	8.67 (7.2, 10.4)
(xii) **AKR1C2 vs. HSD17B2, HSD17B6**	
3α,5α-THP/3β,5α-THP	1.69 (1.2, 2.3)
10^{3}·3α,5α-THP/3β,5α-THP, C	352 (294, 414)
3α,5β-THP/3β,5β-THP, C	7.92 (6.94, 8.96)
10^{3}·3α,5α,20α-PD/3β,5α,20α-PD	179 (130, 253)
10^{3}·3α,5α,20α-PD/3β,5α,20α-PD, C	12.4 (10.3, 14.8)
3α,5β,20α-PD/3β,5β,20α-PD	1.56 (0.918, 2.51)
3α,5β,20α-PD/3β,5β,20α-PD, C	1.37 (1.19, 1.56)
3α,5α,17,20α-PT/3β,5α,17,20α-PT	0.965 (0.728, 1.3)
3α,5α,17,20α-PT/3β,5α,17,20α-PT, C	5.63 (8.48, 3.91)

3α,5α-THA/3β,5α-THA	2.43 (2.97, 1.97)
3α,5α-THA/3β,5α-THA, C	5.22 (4.47, 5.94)
3α,5β-THA/3β,5β-THA, C	3.12 (2.43, 3.92)
3α,5α,17β-AD/3β,5α,17β-AD	3.7 (2.01, 6.52)
3α,5α,17β-AD/3β,5α,17β-AD, C	1.05 (0.812, 1.34)
3α,5β,17β-AD/3β,5β,17β-AD, C	23.1 (28.7, 18.7)
11β-OH-3α,5α-THA/11β-OH-3β,5α-THA	24.5 (29.1, 21)
11β-OH-3α,5α-THA/11β-OH-3β,5α-THA, C	32.2 (24.9, 42.4)
3α,5α,17β-AD/5α-DHT	0.51 (0.355, 0.695)
3α,5α,17β-AD/5α-DHT, C	36.7 (29.5, 45.6)
3α,5α,17β-AD/5α-DHT, C+U	31.5 (25.6, 38.4)
3α,5α-THP/5α-DHP	3.43 (2.29, 5.17)
3α,5α-THPC/5α-DHP	180 (124, 254)
3α,5α-THP, C+U/5α-DHP	187 (130, 263)
3α,5α,20α-PD/5α,20α-THP	2.18 (1.62, 3.02)
3α,5α,20α-PD/5α,20α-THP, C	218 (159, 298)
3α,5α,20α-PD/5α,20α-THP, C+U	132 (102, 164)
3α,5β,20α-PD/5β,20α-THP, C	78.5 (63.2, 96.9)
3α,5α-THA/5α-DHA	2.97 (2.53, 3.56)
10^{-3}·3α,5α-THAC/5α-DHA	14.9 (10.2, 20.9)
10^{-3}·3α,5α-THA, C+U/5α-DHA	14.9 (10.2, 20.9)
(xiii) **HSD17B3, AKR1C3 vs. HSD17B2**	
Adiol/DHEA	0.29 (0.235, 0.363)
Adiol/DHEA, C	0.242 (0.194, 0.303)
3β,7α,17β-AT/7α-OH-DHEA	0.591 (0.499, 0.691)
3β,7β,17β-AT/7β-OH-DHEA	0.57 (0.501, 0.649)
T/A	3.88 (3.29, 4.57)
TC/A	1.63 (1.15, 2.3)
T, C+U/A	5.63 (4.67, 6.85)
5α-DHT/5α-DHA	2.53 (2, 3.23)
5α-DHTC/5α-DHA	15.5 (11.4, 21.6)
5α-DHT, C+U/5α-DHA	18.6 (14.1, 25.2)
3α,5α,17β-A/3α,5α-THA	0.407 (0.311, 0.508)
10^{3}·3α,5α,17β-A/3α,5α-THA, C	39.7 (57.1, 28.7)
10^{3}·3β,5α,17β-A/3β,5α-THA	266 (400, 177)
10^{3}·3β,5α,17β-A/3β,5α-THA, C	232 (288, 187)
10^{3}·3α,5β,17β-AD/3α,5β-THA	53.5 (26.9, 103)
10^{3}·3α,5β,17β-AD/3α,5β-THA, C	133 (100, 172)
10^{3}·3β,5β,17β-AD/3β,5β-THA, C	15 (11.2, 20.3)
