# Supplementary material for: Steroidomic Differences Among Clozapine, Olanzapine, and Risperidone in Male Patients with First-Episode Psychosis, Including a Treatment-Resistant Schizophrenia Subgroup
Source: Int J Mol Sci. 2026 Oct 7;27(19):8909. doi: 10.3390/ijms27198909 (PMC13665165; doi:10.3390/ijms27198909)
Supplement: Supplementary file 1 [file ijms-27-08909-s001.zip › Supplementary Table S4 NS OPLS.pdf]

Table S4. Discrimination between antipsychotic treatment groups based on steroid profiles, evaluated by OPLS and OMR models with insignificant CV-ANOVA but significant predictive component.

Table SA. Discrimination between clozapine- and olanzapine-treated patients based on steroid profiles, a proxy for CYP17A1, hydroxylase + lyase activity, evaluated by OPLS and OMR models

		Orthogonal predictions to latent structure (**OPLS**), **predictive component**					Ordinary multiple regression (**OMR**)		
	**Variable**	Variable importance	t-statistic	Component loading	t-statistic	R[a]	Regression coefficient	t-statistic	
EXPLANATORY VARIABLES (**X**)	DHEA/Preg	0.77	2.05*	-0.417	-3.03	-0.707**	-0.113	-1.86	↓
	DHEA/20α-DHPreg	1.216	7.16**	-0.481	-6.27	-0.815**	-0.178	-2.62*	↓
	A/20α-DHP	1.016	2.57*	-0.474	-6.22	-0.803**	-0.149	-2.23*	↓
	3β,5α-THA/3β,5α,20α-PD	0.826	2.08*	-0.348	-1.93	-0.589*	-0.121	-1.74	↓
	3β,5α-THA/3α,5β-THP, C	1.184	2.39*	-0.419	-2.50	-0.710*	-0.174	-1.69	↓
	11β-OH-A/B	0.901	2.62*	-0.309	-1.83	-0.524	-0.132	-2.24*	↓
**Y**	Clozapine (vs. Olanzapine), LLR			1.000	5.82	0.609**			
	R^2Y=shared variability with explanatory variables (after CV=Q^2), CV-ANOVA			37% (21.7%), F(2, 17)=2.4, p=0.125					

Number of relevant/total explanatory variables=6/24, Shared variability among explanatory variables (R^2X)=48.9%, Predictive/orthogonal components=1/0, Sensitivity=0.667(0.223-0.957), Specificity=0.929(0.661-0.998) (values with 95% CI)

*[a]R=Component loadings expressed as correlation coefficients with predictive components; LLR=logarithm of likelihood ratio; *p<0.05, **p<0.01; CV=cross validation; The p-value of CV-ANOVA (Analysis of variance testing of Cross-Validated predictive residuals) indicates the probability level where a model with this F-value may be the result of just chance.*

Table SB. Discrimination between clozapine- and olanzapine-treated patients based on steroid profiles, a proxy for CYP17A1, hydroxylase activity, evaluated by OPLS and OMR models

		Orthogonal predictions to latent structure (**OPLS**), **predictive component**					Ordinary multiple regression (**OMR**)		
	**Variable**	Variable importance	t-statistic	Component loading	t-statistic	R[a]	Regression coefficient	t-statistic	
EXPL. VARs (**X**)	17-OH-20α-DHP/20α-DHP, C	0.966	3.07*	0.571	4.39	0.778**	0.255	2.12*	↑
	3β,5α,17,20α-PT/3β,5α,20α-PD, C	0.892	2.69*	0.544	4.06	0.741**	0.236	2.25*	↑
	3α,5β,17,20α-PT/3α,5β,20α-PD, C	1.127	3.76**	0.617	3.84	0.841**	0.298	3.10**	↑
**Y**	Clozapine (vs. Olanzapine), LLR			1.000	3.30	0.624**			
	R^2Y=shared variability with explanatory variables (after CV=Q^2), CV-ANOVA			38.9% (28.7%), F(2, 17)=3.4, p=0.056					

Number of relevant/total explanatory variables=3/17, Shared variability among explanatory variables (R^2X)=62.1%, Predictive/orthogonal components=1/0, Sensitivity=0.667(0.223-0.957), Specificity=0.929(0.661-0.998) (values with 95% CI)

*[a]R=Component loadings expressed as correlation coefficients with predictive components; LLR=logarithm of likelihood ratio; *p<0.05, **p<0.01; CV=cross validation; The p-value of CV-ANOVA (Analysis of variance testing of Cross-Validated predictive residuals) indicates the probability level where a model with this F-value may be the result of just chance.*

Table SC. Discrimination between clozapine- and olanzapine-treated patients based on steroid profiles, a proxy for CYP17A1, lyase activity, evaluated by OPLS and OMR models

		Orthogonal predictions to latent structure (**OPLS**), **predictive component**					Ordinary multiple regression (**OMR**)		
	**Variable**	Variable importance	t-statistic	Component loading	t-statistic	R[a]	Regression coefficient	t-statistic	
EXPLANATORY VARIABLES (**X**)	A/P	1.172	3.71**	-0.358	-6.16	-0.832**	-0.101	-2.09*	↓
	A/20α-DHP	1.134	3.71**	-0.342	-4.05	-0.794**	-0.097	-2.35*	↓
	3α,5α-THA/3α,5α,17-PD	0.788	2.23*	-0.308	-4.76	-0.715**	-0.068	-1.69	↓
	3α,5β-THA/3α,5β,17-PD, C	1.022	4.28**	-0.377	-5.30	-0.876**	-0.088	-2.09*	↓
	3β,5α-THA/3β,5α,17,20α-PT	0.634	1.95*	-0.331	-5.15	-0.769**	-0.054	-1.51	↓
	3β,5α-THA/3β,5α,17,20α-PT, C	0.971	3.06**	-0.303	-5.55	-0.704**	-0.083	-2.39*	↓
	3α,5β-THA/3α,5β,17,20α-PT	0.765	2.20*	-0.350	-3.61	-0.812**	-0.066	-1.85	↓
	3α,5β-THA/3α,5β,17,20α-PT, C	1.459	7.01**	-0.383	-13.51	-0.890**	-0.125	-3.02**	↓
	11β-OH-A/F	0.787	3.42**	-0.298	-4.60	-0.603**	-0.068	-3.42**	↓
**Y**	Clozapine (vs. Olanzapine), LLR			1.000	4.77	0.599**			
	R^2Y=shared variability with explanatory variables (after CV=Q^2), CV-ANOVA	35.8% (23.2%), F(2, 17)=2.6, p=0.102							

Number of relevant/total explanatory variables=9/17, Shared variability among explanatory variables (R^2X)=61.3%, Predictive/orthogonal components=1/0, Sensitivity=0.667(0.223-0.957), Specificity=0.667(0.223-0.957) (values with 95% CI)

*[a]R=Component loadings expressed as correlation coefficients with predictive components; LLR=logarithm of likelihood ratio; *p<0.05, **p<0.01; CV=cross validation; The p-value of CV-ANOVA (Analysis of variance testing of Cross-Validated predictive residuals) indicates the probability level where a model with this F-value may be the result of just chance.*

Table SD Discrimination between clozapine- and olanzapine-treated patients based on steroid profiles, a proxy for the balance between HSD11B1 and HSD11B2, evaluated by OPLS and OMR models

		Orthogonal predictions to latent structure (**OPLS**), **predictive component**					Ordinary multiple regression (**OMR**)		
	**Variable**	Variable importance	t-statistic	Component loading	t-statistic	R[a]	Regression coefficient	t-statistic	
EXPL. VARS (**X**)	7β-OH-DHEA/7α-OH-DHEA	0.972	6.39**	0.574	9.85	0.737**	0.256	1.37	↑
	3β,7β,17β-AT/3β,7α,17β-AT	0.929	4.20**	0.637	4.29	0.779**	0.244	1.16	↑
	F/E	1.091	5.69**	0.533	4.24	0.688**	0.287	1.14	↑
**Y**	Clozapine (vs. Olanzapine), LLR			1.000	2.37	0.559*			
	R^2Y=shared variability with explanatory variables (after CV=Q^2), CV-ANOVA	31.3% (-6.9%), F(2, 17)=0, p=1							

Number of relevant/total explanatory variables=3/5, Shared variability among explanatory variables (R^2X)=54.2%, Predictive/orthogonal components=1/0, Sensitivity=0.667(0.223-0.957), Specificity=1(0.768-1) (values with 95% CI)

*[a]R=Component loadings expressed as correlation coefficients with predictive components; LLR=logarithm of likelihood ratio; *p<0.05, **p<0.01; CV=cross validation; The p-value of CV-ANOVA (Analysis of variance testing of Cross-Validated predictive residuals) indicates the probability level where a model with this F-value may be the result of just chance.*

Table SE. Discrimination between clozapine- and olanzapine-treated patients based on steroid profiles, a proxy for activities of CYP7B1, CYP3A4, CYP3A7 enzymes, evaluated by OPLS and OMR models

		Orthogonal predictions to latent structure (**OPLS**), **predictive component**					Ordinary multiple regression (**OMR**)		
	**Variable**	Variable importance	t-statistic	Component loading	t-statistic	R^a	Regression coefficient	t-statistic	
EXP. VAR. (**X**)	7β-OH-DHEA/DHEA	1		1.000		1.000	0.614	3.68 **	↑
**Y**	Clozapine (vs. Olanzapine), LLR			1.000	2.35	0.614*			
	R^2Y=shared variability with explanatory variables (after CV=Q^2), CV-ANOVA	37.7% (32%), F(2, 15)=3.5, p=0.055							

Number of relevant/total explanatory variables=1/9, Shared variability among explanatory variables (R^2X)=100%, Predictive/orthogonal components=1/0, Sensitivity=0.667(0.223-0.957), Specificity=0.917(0.615-0.998) (values with 95% CI)

*[a]R=Component loadings expressed as correlation coefficients with predictive components; LLR=logarithm of likelihood ratio; *p<0.05, **p<0.01; CV=cross validation; The p-value of CV-ANOVA (Analysis of variance testing of Cross-Validated predictive residuals) indicates the probability level where a model with this F-value may be the result of just chance.*

Table SF. Discrimination between clozapine- and olanzapine-treated patients based on steroid profiles, a proxy for SRD5A activities, evaluated by OPLS and OMR models

		Orthogonal predictions to latent structure (**OPLS**), **predictive component**					Ordinary multiple regression (**OMR**)		
	**Variable**	Variable importance	t-statistic	Component loading	t-statistic	R^a	Regression coefficient	t-statistic	
EXPLANATORY VARIABLES (**X**)	3α,5α,17-PD/17-OH-P	0.905	2.24*	0.339	4.65	0.650**	0.104	1.63	↑
	3α,5α,17-PDC/17-OH-P	0.91	3.13**	0.432	5.31	0.828**	0.104	2.30*	↑
	3α,5α,17-PD, C+U/17-OH-P	0.913	3.14**	0.433	5.42	0.831**	0.104	2.28*	↑
	(5α-DHA+3α,5α-THA+3β,5α-THA)/A	0.821	2.54*	0.306	4.59	0.587**	0.094	2.88*	↑
	(3α,5α-THA+3β,5α-THA, C)/A	1.192	3.05**	0.402	3.63	0.772**	0.136	3.06**	↑
	(5α-DHA+3α,5α-THAC +3β,5α-THAC)/A	1.193	3.06**	0.402	3.64	0.772**	0.137	3.07**	↑
	(5α-DHT+3α,5α,17β-AD +3β,5α,17β-AD)/T	1.001	3.43**	0.339	3.43	0.651**	0.115	2.58*	↑
**Y**	Clozapine (vs. Olanzapine), LLR			1.000	4.22	0.581**			
	R^2Y=shared variability with explanatory variables (after CV=Q^2), CV-ANOVA	33.8% (24.9%), F(2, 17)=2.8, p=0.088							

Number of relevant/total explanatory variables=7/22, Shared variability among explanatory variables (R^2X)=53.7%, Predictive/orthogonal components=1/0, Sensitivity=0.667(0.223-0.957), Specificity=0.929(0.661-0.998) (values with 95% CI)

*[a]R=Component loadings expressed as correlation coefficients with predictive components; LLR=logarithm of likelihood ratio; *p<0.05, **p<0.01; CV=cross validation; The p-value of CV-ANOVA (Analysis of variance testing of Cross-Validated predictive residuals) indicates the probability level where a model with this F-value may be the result of just chance.*

Table SG. Discrimination between clozapine- and olanzapine-treated patients based on steroid profiles, a proxy for AKR1D1 activity, evaluated by OPLS and OMR models

	Variable	Orthogonal predictions to latent structure (**OPLS**), **predictive component**					Ordinary multiple regression (**OMR**)		
		Variable importance	t-statistic	Component loading	t-statistic	R[a]	Regression coefficient	t-statistic	
EXPLANAT. VARIABLES (X)	17-OH-20α-DHP/17-OH-P	0.847	2.00*	0.388	1.28	0.454	0.246	1.97*	↑
	3α,5β,20α-PD/3α,5β-THP	0.915	2.44*	0.636	3.09	0.744**	0.266	2.58*	↑
	3α,5β,20α-PD/3α,5β-THP, C	1.181	2.27*	-0.452	-1.12	-0.529	-0.343	-1.39	↓
	3α,5β,17,20α-PT/3α,5β,17-PD, C	1.025	2.61*	0.542	2.46	0.634*	0.298	2.53*	↑
**Y**	Clozapine (vs. Risperidone), LLR			1.000	3.59	0.680**			
	R^2Y=shared variability with explanatory variables (after CV=Q^2), CV-ANOVA			61.2% (42.4%), F(2, 17)=3.3, p=0.061					

Number of relevant/total explanatory variables=4/20, Shared variability among explanatory variables (R^2X)=36%, Predictive/orthogonal components=1/0, Sensitivity=0.8(0.284-0.995), Specificity=0.929(0.661-0.998) (values with 95% CI)

*[a]R=Component loadings expressed as correlation coefficients with predictive components; LLR=logarithm of likelihood ratio; *p<0.05, **p<0.01; CV=cross validation; The p-value of CV-ANOVA (Analysis of variance testing of Cross-Validated predictive residuals) indicates the probability level where a model with this F-value may be the result of just chance.*

Table SH. Discrimination between clozapine- and risperidone-treated patients based on steroid profiles, a proxy for CYP17A1 hydroxylase + lyase activity, evaluated by OPLS and OMR models

	Variable	Orthogonal predictions to latent structure (**OPLS**), **predictive component**					Ordinary multiple regression (**OMR**)		
		Variable importance	t-statistic	Component loading	t-statistic	R[a]	Regression coefficient	t-statistic	
EXPLANATORY VARIABLES (X)	Age	1.105	2.51*	-0.429	-6.40	-0.774**	-0.162	-1.87	↓
	A/20α-DHP	1.15	2.42*	-0.410	-4.45	-0.741**	-0.169	-1.64	↓
	3β,5α-THA/3β,5α-THP	1.035	2.43*	-0.390	-2.76	-0.705*	-0.152	-2.50*	↓
	3β,5α-THA/3β,5α-THP, C	0.982	2.26*	-0.411	-2.80	-0.743*	-0.144	-2.37*	↓
	3β,5α-THA/3β,5α,20α-PD, C	0.57	2.01*	-0.461	-3.12	-0.833**	-0.084	-1.81	↓
	11β-OH-A/B	1.046	2.43*	-0.414	-3.25	-0.748**	-0.153	-1.72	↓
**Y**	Clozapine (vs. Risperidone), LLR			1.000	4.68	0.649**			
	R^2Y=shared variability with explanatory variables (after CV=Q^2), CV-ANOVA			42.2% (25.3%), F(2, 8)=1.4, p=0.311					

Number of relevant/total explanatory variables=6/24, Shared variability among explanatory variables (R^2X)=57.5%, Predictive/orthogonal components=1/0, Sensitivity=0.833(0.359-0.996), Specificity=0.6(0.147-0.947) (values with 95% CI)

*[a]R=Component loadings expressed as correlation coefficients with predictive components; LLR=logarithm of likelihood ratio; *p<0.05, **p<0.01; CV=cross validation; The p-value of CV-ANOVA (Analysis of variance testing of Cross-Validated predictive residuals) indicates the probability level where a model with this F-value may be the result of just chance.*

Table SI. Discrimination between clozapine- and risperidone-treated patients based on steroid profiles, a proxy for CYP17A1 hydroxylase activity, evaluated by OPLS and OMR models

		Orthogonal predictions to latent structure (**OPLS**), **predictive component**					Ordinary multiple regression (**OMR**)		
	**Variable**	Variable importance	t-statistic	Component loading	t-statistic	R[a]	Regression coefficient	t-statistic	
EXPL. VARS. (X)	Age	0.947	5.03**	-0.592	-8.31	-0.925**	-0.217	-2.44*	↓
	17-OH-Preg/Preg, C	0.788	3.32**	0.567	8.21	0.885**	0.180	2.45*	↑
	F/B	1.218	6.43**	-0.595	-6.21	-0.930**	-0.279	-5.37**	↓
**Y**	Clozapine (vs. Risperidone), LLR			1.000	1.96	0.620*			
	R^2Y=shared variability with explanatory variables (after CV=Q^2), CV-ANOVA	38.4% (29.3%), F(2, 8)=1.7, p=0.25							

Number of relevant/total explanatory variables=3/17, Shared variability among explanatory variables (R^2X)=83.5%, Predictive/orthogonal components=1/0, Sensitivity=0.833(0.359-0.996), Specificity=0.8(0.284-0.995) (values with 95% CI)

*[a]R=Component loadings expressed as correlation coefficients with predictive components; LLR=logarithm of likelihood ratio; *p<0.05, **p<0.01; CV=cross validation; The p-value of CV-ANOVA (Analysis of variance testing of Cross-Validated predictive residuals) indicates the probability level where a model with this F-value may be the result of just chance.*

Table SJ. Discrimination between clozapine- and risperidone-treated patients based on steroid profiles, a proxy for CYP17A1 lyase activity, evaluated by OPLS and OMR models

		Orthogonal predictions to latent structure (**OPLS**), **predictive component**					Ordinary multiple regression (**OMR**)		
	**Variable**	Variable importance	t-statistic	Component loading	t-statistic	R[a]	Regression coefficient	t-statistic	
EXPLANAT. VARS. (X)	Age	1.056	1.83	-0.380	-2.45	-0.663 *	-0.174	-1.11	↓
	A/17-OH-20α-DHP	0.971	2.05 *	-0.371	-2.52	-0.647 *	-0.160	-1.52	↓
	3α,5α-THA/3α,5α,17-PD	0.969	2.23 *	-0.442	-2.83	-0.773 *	-0.159	-1.82	↓
	3α,5α-THA/3α,5α,17-PD, C	1.037	3.03 **	-0.529	-4.63	-0.923 **	-0.171	-3.12 **	↓
	3α,5β-THA/3α,5β,17,20α-PT, C	0.962	3.13 **	-0.517	-5.66	-0.903 **	-0.158	-2.40 *	↓
**Y**	Clozapine (vs. Risperidone), LLR			1.000	2.39	0.642 *			
	R^2Y=shared variability with explanatory variables (after CV=Q^2), CV-ANOVA	41.2% (19.7%), F(2, 8)=1, p=0.416							

Number of relevant/total explanatory variables=5/17, Shared variability among explanatory variables (R^2X)=62.5%, Predictive/orthogonal components=1/0, Sensitivity=0.833(0.359-0.996), Specificity=0.6(0.147-0.947) (values with 95% CI)

*[a]R=Component loadings expressed as correlation coefficients with predictive components; LLR=logarithm of likelihood ratio; *p<0.05, **p<0.01; CV=cross validation; The p-value of CV-ANOVA (Analysis of variance testing of Cross-Validated predictive residuals) indicates the probability level where a model with this F-value may be the result of just chance.*

Table SK. Discrimination between clozapine- and risperidone-treated patients based on steroid profiles, a proxy for HSD3Bs activities, evaluated by OPLS and OMR models

		Orthogonal predictions to latent structure (**OPLS**), **predictive component**					Ordinary multiple regression (**OMR**)		
	**Variable**	Variable importance	t-statistic	Component loading	t-statistic	R^a	Regression coefficient	t-statistic	
EXPL. VARS. (**X**)	Age	0.928	3.14**	-0.493	-5.61	-0.892**	-0.161	-1.95*	↓
	P/Preg, C	1.02	3.58**	0.516	4.89	0.873**	0.177	4.14**	↑
	20α-DHP/20α-DHPreg, C	1.217	6.02**	0.529	5.42	0.958**	0.212	2.98*	↑
	T/Adiol	0.785	3.57**	-0.474	-7.32	-0.859**	-0.136	-2.73*	↓
**Y**	Clozapine (vs. Risperidone), LLR			1.000	3.37	0.630**			
	R^2Y=shared variability with explanatory variables (after CV=Q^2), CV-ANOVA	39.7% (30.8%), F(2, 8)=1.8, p=0.228							
Number of relevant/total explanatory variables=4/14, Shared variability among explanatory variables (R^2X)=80.4%, Predictive/orthogonal components=1/0, Sensitivity=0.833(0.359-0.996), Specificity=0.8(0.284-0.995) (values with 95% CI)									

*[a]R=Component loadings expressed as correlation coefficients with predictive components; LLR=logarithm of likelihood ratio; *p<0.05, **p<0.01; CV=cross validation; The p-value of CV-ANOVA (Analysis of variance testing of Cross-Validated predictive residuals) indicates the probability level where a model with this F-value may be the result of just chance.*

Table SL. Discrimination between clozapine- and olanzapine-treated patients based on conjugated-to-unconjugated steroid ratios (C/U), a proxy for the balance between SULT2A1 and STS activities, evaluated by OPLS and OMR models

		Orthogonal predictions to latent structure (**OPLS**), **predictive component**					Ordinary multiple regression (**OMR**)		
	**Variable**	Variable importance	t-statistic	Component loading	t-statistic	R^a	Regression coefficient	t-statistic	
EXPLANATORY VARIABLES (**X**)	Age	0.929	2.68*	-0.360	-7.00	-0.849**	-0.095	-2.03*	↓
	20α-DHPreg, C/U	0.973	2.67*	-0.327	-4.58	-0.772**	-0.099	-2.79*	↓
	20α-DHP, C/U	0.863	2.10*	-0.291	-3.04	-0.686**	-0.088	-1.78	↓
	5α-DHT, C/U	0.811	2.34*	-0.208	-2.74	-0.491*	-0.083	-2.34*	↓
	3β,5α-THP, C/U	1.004	3.20**	-0.354	-5.33	-0.836**	-0.103	-3.28**	↓
	5α,20α-THP, C/U	0.807	2.09*	-0.305	-5.23	-0.721**	-0.082	-1.53	↓
	3β,5α-THA, C/U	0.736	1.90*	-0.350	-4.33	-0.826**	-0.075	-1.98*	↓
	3β,5α,17β-AD, C/U	1.142	4.05**	-0.371	-6.87	-0.876**	-0.117	-3.45**	↓
	3α,5β,17β-AD, C/U	1.607	4.02**	-0.350	-4.70	-0.827**	-0.164	-2.76*	↓
	11β-OH-3β,5α-THA, C/U	0.837	2.42*	-0.292	-3.53	-0.691**	-0.085	-2.66*	↓
**Y**	Clozapine (vs. Risperidone), LLR			1.000	7.81	0.763 **			
	R^2Y=shared variability with explanatory variables (after CV=Q^2), CV-ANOVA	58.2% (44.6%), F(2, 8)=3.2, p=0.094							
Number of relevant/total explanatory variables=10/33, Shared variability among explanatory variables (R^2X)=58.6%, Predictive/orthogonal components=1/0, Sensitivity=1(0.541-1), Specificity=0.8(0.284-0.995) (values with 95% CI)									

*[a]R=Component loadings expressed as correlation coefficients with predictive components; LLR=logarithm of likelihood ratio; *p<0.05, **p<0.01; CV=cross validation; The p-value of CV-ANOVA (Analysis of variance testing of Cross-Validated predictive residuals) indicates the probability level where a model with this F-value may be the result of just chance.*

Table SM. Discrimination between clozapine- and olanzapine-treated patients based on steroid profiles, a proxy for the balance between HSD11B1 and HSD11B2 activities, evaluated by OPLS and OMR models

	Variable	Orthogonal predictions to latent structure (**OPLS**), **predictive component**					Ordinary multiple regression (**OMR**)		
		Variable importance	t-statistic	Component loading	t-statistic	R^a	Regression coefficient	t-statistic	
EXPL. VARS. (X)	Age	0.954	2.40*	-0.682	-1.59	-0.651	-0.581	-2.09*	↓
	F/E	1.044	2.69*	0.731	2.07	0.682*	0.636	2.81*	↑
**Y**	Clozapine (vs. Risperidone), LLR			1.000	2.51	0.822*			
	R^2Y=shared variability with explanatory variables (after CV=Q^2), CV-ANOVA	67.6% (45.5%), F(2, 8)=3.3, p=0.088							
Number of relevant/total explanatory variables=2/5, Shared variability among explanatory variables (R^2X)=44.3%, Predictive/orthogonal components=1/0, Sensitivity=0.833(0.359-0.996), Specificity=1(0.478-1) (values with 95% CI)									

*[a]R=Component loadings expressed as correlation coefficients with predictive components; LLR=logarithm of likelihood ratio; *p<0.05, **p<0.01; CV=cross validation; The p-value of CV-ANOVA (Analysis of variance testing of Cross-Validated predictive residuals) indicates the probability level where a model with this F-value may be the result of just chance.*

Table SN. Discrimination between clozapine- and risperidone-treated patients based on steroid profiles, a proxy for AKR1D1 activity, evaluated by OPLS and OMR models

	Variable	Orthogonal predictions to latent structure (**OPLS**), **predictive component**					Ordinary multiple regression (**OMR**)		
		Variable importance	t-statistic	Component loading	t-statistic	R^a	Regression coefficient	t-statistic	
EXPL. VARS. (X)	Age	0.863	2.00*	-0.432	-2.33	-0.610*	-0.266	-1.82	↓
	3α,5β-THP/P	0.909	2.64*	-0.485	-4.19	-0.591**	-0.280	-3.05**	↓
	3α,5β,17β-AD/T	1.272	3.93**	0.574	4.43	0.810**	0.392	3.32**	↑
	11β-OH-3α,5β-THA/11β-OH-A	0.9	3.84**	0.506	5.10	0.714**	0.277	2.30*	↑
**Y**	Clozapine (vs. Risperidone), LLR			1.000	7.59	0.870**			
	R^2Y=shared variability with explanatory variables (after CV=Q^2), CV-ANOVA	75.7% (57.7%), F(2, 8)=5.8, p=0.028							
Number of relevant/total explanatory variables=4/22, Shared variability among explanatory variables (R^2X)=47.5%, Predictive/orthogonal components=1/0, Sensitivity=1(0.541-1), Specificity=1(0.478-1) (values with 95% CI)									

*[a]R=Component loadings expressed as correlation coefficients with predictive components; LLR=logarithm of likelihood ratio; *p<0.05, **p<0.01; CV=cross validation; The p-value of CV-ANOVA (Analysis of variance testing of Cross-Validated predictive residuals) indicates the probability level where a model with this F-value may be the result of just chance.*

Table SO. Discrimination between olanzapine- and risperidone-treated patients based on steroid profiles evaluated by OPLS and OMR models

		Orthogonal predictions to latent structure (**OPLS**), **predictive component**					Ordinary multiple regression (**OMR**)		
	**Variable**	Variable importance	t-statistic	Component loading	t-statistic	R[a]	Regression coefficient	t-statistic	
EXPLANATORY VARIABLES (**X**)	Pregnenolone	1.018	5.42**	0.192	5.50	0.850**	0.023	2.74*	↑
	20α-Dihydropregnenolone	0.919	2.40*	0.166	6.43	0.735**	0.020	1.87	↑
	17-Hydroxypregnenolone	1.024	2.94*	0.182	4.45	0.805**	0.023	2.06*	↑
	17-Hydroxypregnenolone, C	1.092	5.27**	0.178	10.97	0.788**	0.024	4.05**	↑
	16α-Hydroxypregnenolone	0.849	2.53*	0.088	9.62	0.389**	0.019	2.42*	↑
	Dehydroepiandrosterone	1.212	2.81*	0.180	7.18	0.794**	0.027	2.29*	↑
	7α-Hydroxy-DHEA	1.163	6.38**	0.209	17.69	0.922**	0.026	3.36**	↑
	7β-Hydroxy-DHEA	1.057	8.22**	0.191	8.25	0.845**	0.023	3.41**	↑
	Androstenediol	0.876	1.97*	0.175	3.78	0.772**	0.019	1.60	↑
	5-Androstene-3β,7α,17β-triol	0.934	1.93*	0.160	2.98	0.709*	0.021	1.97*	↑
	Progesterone	0.743	2.13*	0.120	2.08	0.532*	0.016	2.89*	↑
	20α-Dihydroprogesterone	0.939	2.96*	0.197	6.11	0.871**	0.021	2.54*	↑
	17-Hydroxyprogesterone	0.674	2.03*	0.165	4.70	0.731**	0.015	1.58	↑
	17,20α-Dihydroxy-4-pregnene-3-one	1.069	2.90*	0.167	5.09	0.740**	0.024	3.73**	↑
	Androstenedione	1.274	3.15**	0.161	3.38	0.712**	0.028	2.08*	↑
	5α-Dihydroprogesterone	1.138	5.02**	0.190	6.91	0.838**	0.025	3.47**	↑
	Allopregnanolone	0.764	2.62*	0.172	5.11	0.761**	0.017	2.32*	↑
	Allopregnanolone, C	0.938	3.20**	0.186	5.21	0.823**	0.021	3.18**	↑
	Isopregnanolone	1.045	5.94**	0.197	11.66	0.869**	0.023	3.21**	↑
	Isopregnanolone, C	0.741	4.06**	0.176	6.86	0.778**	0.016	3.98**	↑
	Pregnanolone	1.052	3.71**	0.154	7.30	0.682**	0.023	5.06**	↑
	Pregnanolone, C	0.819	3.26**	0.169	4.64	0.748**	0.018	2.93*	↑
	17-Hydroxyallopregnanolone, C	0.808	3.55**	0.179	5.97	0.792**	0.018	2.83*	↑
	17-Hydroxypregnanolone, C	0.697	2.03*	0.123	4.67	0.543**	0.015	1.74	↑
	5α,20α-Tetrahydroprogesterone	1.143	5.31**	0.205	8.98	0.906**	0.025	3.79**	↑
	5β-Pregnane-3α,20α-diol	0.858	2.25*	0.150	5.21	0.661**	0.019	3.07**	↑
	5α-Pregnane-3α,17,20α-triol	1.079	4.06**	0.176	7.40	0.775**	0.024	2.96*	↑
	5β-Pregnane-3α,17,20α-triol	0.887	2.29*	0.167	4.19	0.737**	0.020	2.40*	↑
	5α-Androstane-3,17-dione	1.168	2.51*	0.152	2.93	0.670*	0.026	2.03*	↑
	Androsterone	1.119	4.02**	0.086	2.55	0.381*	0.025	3.88**	↑
	Epiandrosterone	1.405	6.47**	0.166	4.94	0.735**	0.031	4.91**	↑
	5α-Androstane-3α,17β-diol	0.971	6.09**	0.136	3.70	0.600**	0.021	3.33**	↑
	5β-Androstane-3α,17β-diol	1.118	3.09**	0.165	5.71	0.729**	0.025	3.02**	↑
	Cortisol	0.992	2.59*	0.156	4.23	0.693**	0.022	2.24*	↑
	Corticosterone	0.732	2.19*	0.159	4.70	0.701**	0.016	1.63	↑
	21-Deoxycortisol	0.397	3.07**	0.089	2.42	0.395*	0.009	2.17*	↑
	11β-Hydroxyandrosterone	1.246	3.09**	0.176	5.98	0.777**	0.028	2.93*	↑
	11β-Hydroxyandrosterone, C	1.235	1.94*	0.057	0.99	0.251	0.027	1.74	↑
	11β-Hydroxyepiandrosterone	1.037	3.09**	0.188	7.59	0.828**	0.023	2.43*	↑
**Y**	Olanzapine (vs. Risperidone), LLR			1.000	11.57	0.611**			
	R^2Y=shared variability with explanatory variables (after CV=Q^2), CV-ANOVA			37.3% (28.2%), F(2, 16)=3.1, p=0.071					

Number of relevant/total explanatory variables=39/86, Shared variability among explanatory variables (R^2X)=53.4%, Predictive/orthogonal components=1/0, Sensitivity=0.6(0.147-0.947), Specificity=0.857(0.572-0.982) (values with 95% CI)

[a]*R=Component loadings expressed as correlation coefficients with predictive components; LLR=logarithm of likelihood ratio; *p<0.05, **p<0.01; CV=cross validation; The p-value of CV-ANOVA (Analysis of variance testing of Cross-Validated predictive residuals) indicates the probability level where a model with this F-value may be the result of just chance.*

Table SP. Discrimination between olanzapine- and risperidone-treated patients based on steroid profiles, a proxy for HSD3Bs activities, evaluated by OPLS and OMR models

		Orthogonal predictions to latent structure (**OPLS**), **predictive component**					Ordinary multiple regression (**OMR**)		
	**Variable**	Variable importance	t-statistic	Component loading	t-statistic	R^a	Regression coefficient	t-statistic	
EXPL. VARS. (**X**)	P/PregC	0.806	2.67*	0.428	3.75	0.557**	0.171	2.74*	↑
	20α-DHP/20α-DHPreg, C	1.144	3.56**	0.632	8.01	0.864**	0.243	2.33*	↑
	16α-OH-P/16α-OH-Preg	0.864	2.08*	-0.374	-1.27	-0.512	-0.184	-2.19*	↓
	A/DHEAC	1.139	3.30**	0.536	3.15	0.734**	0.242	1.98*	↑
**Y**	Olanzapine (vs. Risperidone), LLR			1.000	2.84	0.582*			
	R^2Y=shared variability with explanatory variables (after CV=Q^2), CV-ANOVA			33.8% (23.3%), F(2, 16)=2.5, p=0.115					

Number of relevant/total explanatory variables=4/14, Shared variability among explanatory variables (R^2X)=46.9%, Predictive/orthogonal components=1/0, Sensitivity=0.857(0.572-0.982), Specificity=0.6(0.147-0.947) (values with 95% CI)

[a]*R=Component loadings expressed as correlation coefficients with predictive components; LLR=logarithm of likelihood ratio; *p<0.05, **p<0.01; CV=cross validation; The p-value of CV-ANOVA (Analysis of variance testing of Cross-Validated predictive residuals) indicates the probability level where a model with this F-value may be the result of just chance.*

Table SQ. Discrimination between olanzapine- and risperidone-treated patients based on 20α-hydroxy/20-oxo steroid molar ratios, a proxy for the balance between AKR1C1 and HSD17B2 activities, evaluated by OPLS and OMR models)

		Orthogonal predictions to latent structure (**OPLS**), **predictive component**					Ordinary multiple regression (**OMR**)		
	**Variable**	Variable importance	t-statistic	Component loading	t-statistic	R^a	Regression coefficient	t-statistic	
EXPL. VARS. (**X**)	5α,20α-THPC/5α-DHP	1.116	4.51**	-0.802	-8.63	-0.917**	-0.282	-2.41*	↓
	3β,5α,20α-PDC/3β,5α-THP	1.120	3.59**	-0.690	-2.98	-0.789*	-0.283	-3.00**	↓
	3β,5α,20α-PD/3β,5α-THP, C	0.706	1.93*	0.092	0.31	0.105	0.178	1.63	↑
**Y**	Olanzapine (vs. Risperidone), LLR			1.000	2.33	0.500*			
	R^2Y=shared variability with explanatory variables (after CV=Q^2), CV-ANOVA			25% (13.8%), F(2, 16)=1.3, p=0.305					

Number of relevant/total explanatory variables=3/21, Shared variability among explanatory variables (R^2X)=49.1%, Predictive/orthogonal components=1/0, Sensitivity=0.929(0.661-0.998), Specificity=0.4(0.053-0.853) (values with 95% CI)

[a]*R=Component loadings expressed as correlation coefficients with predictive components; LLR=logarithm of likelihood ratio; *p<0.05, **p<0.01; CV=cross validation; The p-value of CV-ANOVA (Analysis of variance testing of Cross-Validated predictive residuals) indicates the probability level where a model with this F-value may be the result of just chance.*

Table SR. Discrimination between olanzapine- and risperidone-treated patients based on 3α/3β steroid molar ratios, a proxy for the balance between AKR1C2 and HSD17B2/HSD17B6 activities, evaluated by OPLS and OMR models c

Orthogonal predictions to latent structure (**OPLS**), **predictive component**	Ordinary multiple regression (**OMR**)

	Variable	Variable importance	t-statistic		Component loading	t-statistic	R^a		Regression coefficient	t-statistic		
EXPLANATORY VARIABLES (**X**)	T/A	0.963	4.42	**	-0.442	-17.25	-0.809	**	-0.109	-1.60		↓
	TC/A	0.89	2.00	*	-0.330	-2.39	-0.604	*	-0.100	-1.62		↓
	T, C+U/A	1.22	3.72	**	-0.448	-5.28	-0.819	**	-0.138	-2.31	*	↓
	5α-DHT/5α-DHA	1.2	2.33	*	-0.408	-3.56	-0.746	**	-0.135	-1.78		↓
	5α-DHTC/5α-DHA	0.801	3.63	**	-0.413	-7.95	-0.756	**	-0.090	-1.92	*	↓
	5α-DHT, C+U/5α-DHA	0.843	4.14	**	-0.431	-6.91	-0.788	**	-0.095	-1.93	*	↓
**Y**	Olanzapine (vs. Risperidone), LLR				1.000	2.27	0.505	*				
	R^2Y=shared variability with explanatory variables (after CV=Q^2), CV-ANOVA				25.5% (9.1%), F(2, 16)=0.8, p=0.465							
	Number of relevant/total explanatory variables=6/18, Shared variability among explanatory variables (R^2X)=57.3%, Predictive/orthogonal components=1/0, Sensitivity=0.857(0.572-0.982), Specificity=0.4(0.053-0.853) (values with 95% CI)											

*[a]R=Component loadings expressed as correlation coefficients with predictive components; LLR=logarithm of likelihood ratio; *p<0.05, **p<0.01; CV=cross validation; The p-value of CV-ANOVA (Analysis of variance testing of Cross-Validated predictive residuals) indicates the probability level where a model with this F-value may be the result of just chance.*
