# Supplementary material for: Steroidomic Differences Among Clozapine, Olanzapine, and Risperidone in Male Patients with First-Episode Psychosis, Including a Treatment-Resistant Schizophrenia Subgroup
Source: Int J Mol Sci. 2026 Oct 7;27(19):8909. doi: 10.3390/ijms27198909 (PMC13665165; doi:10.3390/ijms27198909)
Supplement: Supplementary file 1 [file ijms-27-08909-s001.zip › Supplementary Table S5 Analytical criteria.pdf]

Table S5. Correlation coefficients, linear ranges, detection and quantification limits, and precision for steroid analytes

No	Steroid	Correlation Coefficient (r)	Linear Dynamic Range [pg injected]	LOD [pg]	LOQ [pg] (bias + precision at LOQ)	Level [pg inj.]/[nM]	Intra-/Inter-assay [%]
1	Pregnenolone	0.9995	0.077-2000	0.02	2 (5.8%, 18%)	32/5.1	1.3/12
2	17-Hydroxypregnenolone	0.9996	0.12-2000	0.05	0.5 (-5.8%, 10%)	80/12	1.5/11
3	16α-Hydroxypregnenolone	0.9997	0.12-2000	0.01	0.5 (13%, 3.3%)	2.9/0.43	4.8/8.5
4	20α-Dihydropregnenolone	0.9991	0.12-2000	0.03	0.5 (-6%, 11%)	15/2.3	4.3/9.8
5	Dehydroepiandrosterone (DHEA)	0.9978	0.12-2000	0.01	2 (9.5%, 6%)	58/10	1.4/6.8
6	7α-Hydroxy-DHEA	0.9995	0.12-2000	0.02	0.5 (-1.7%, 11%)	7.9/1.3	1.6/8.3
7	7-oxo-DHEA	0.9952	0.49-2000	0.09	0.5 (7.7%, 11%)	6.6/1.1	6.7/11
8	7β-Hydroxy-DHEA	0.9987	0.49-2000	0.03	0.5 (3.2%, 13%)	2.9/0.48	7.1/14
9	Androstenediol	0.9979	0.49-2000	0.1	2 (0.61%, 10%)	15/2.5	1.4/6.7
10	5-Androstene-3β,7α,17β-triol	0.9999	0.49-2000	0.02	0.5 (15%, 4.9%)	2.3/0.37	2.7/10
11	5-Androstene-3β,7β,17β-triol	0.9993	0.12-2000	0.02	0.5 (13%, 8.4%)	1.9/0.31	8.6/12
12	5-Androstene-3β,16α,17β-triol	0.9985	0.49-2000	0.04	0.5 (-2.6%, 20%)	3.1/0.51	13/12
13	Progesterone	0.9998	0.12-10000	30	0.5 (-2.1%, 12%)	1.5/0.24	6.3/11
14	17-Hydroxyprogesterone	0.9997	0.12-2000	0.1	0.5 (5.1%, 11%)	18/2.8	4.3/9.1
15	17,20α-Dihydroxy-4-pregnene-3-one	0.9957	0.12-10000	1	0.5 (6.3%, 7.6%)	10/1.5	1.3/8.6
16	16α-Hydroxyprogesterone	0.9998	0.12-2000	0.02	0.5 (7.2%, 3.6%)	5/0.76	3.3/11
17	20α-Dihydroprogesterone	0.9997	0.49-2000	0.02	0.5 (13%, 8.6%)	1.3/0.21	6.9/8.9
18	Androstenedione	0.9988	0.49-2000	0.09	2 (-4.3%, 9.8%)	15/2.6	0.88/11
19	Testosterone	0.9998	2.0-2000	0.02	2 (18%, 1.6%)	86/15	2.2/8
20	5α-Dihydrotestosterone	0.9994	0.49-2000	0.04	0.5 (9.5%, 5.8%)	8.7/1.5	6.4/9.1
21	5α-Dihydroprogesterone	0.9995	0.12-10000	0.2	2 (9%, 17%)	0.22/0.035	9.1/6.3
22	Allopregnanolone	0.9996	0.12-2000	0.02	0.5 (1.9%, 11%)	0.43/0.068	13/15
23	Isopregnanolone	0.9995	0.49-2000	0.02	0.5 (4%, 15%)	1.6/0.25	3.5/9.8
24	Pregnanolone	0.9995	0.12-2000	0.04	0.5 (-9.9%, 10%)	0.086/0.014	5.7/8.8
25	5α,20α-Tetrahydroprogesterone	0.9995	0.12-2000	0.2	0.5 (-4.3%, 11%)	1.5/0.24	11/7.1
26	5α-Pregnane-3α,20α-diol	0.9995	0.12-10000	0.6	0.5 (16%, 6.1%)	1.7/0.26	5.3/5
27	5α-Pregnane-3β,20α-diol	0.9987	7.8-10000	2	0.5 (17%, 3.5%)	9/1.4	12/12
28	5β-Pregnane-3α,20α-diol	0.9995	0.12-2000	0.2	0.5 (-2.2%, 16%)	1.8/0.28	14/11
29	5β-Pregnane-3β,20α-diol	0.9997	0.49-10000	0.5	0.5 (6.4%, 13%)	0.44/0.068	13/14
30	17-Hydroxyallopregnanolone	0.9994	0.49-2000	0.2	0.1 (-1.8%, 13%)	0.51/0.077	19/14
31	17-Hydroxypregnanolone	0.9995	0.49-2000	0.1	0.5 (-3.5%, 13%)	0.8/0.12	13/9.1
32	5α-Pregnane-3α,17,20α-triol	0.9981	0.12-10000	0.07	0.5 (0.56%, 11%)	1.9/0.28	3/14
33	5α-Pregnane-3β,17,20α-triol	0.9977	0.12-10000	0.1	0.5 (6%, 9.4%)	1.6/0.24	3/8.5
34	5β-Pregnane-3α,17,20α-triol	0.9982	0.12-10000	0.06	0.5 (0.33%, 12%)	10/1.5	1.9/9.1
35	5α-Androstane-3,17-dione	0.9993	0.12-10000	0.3	0.5 (3.6%, 12%)	1.6/0.27	5.7/8.8
36	Androsterone	0.9987	0.12-2000	0.1	0.5 (5.7%, 7.5%)	2.7/0.46	2.6/12
37	Epiandrosterone	0.9991	2.0-2000	0.03	0.5 (9.9%, 6.6%)	1.8/0.31	1.9/12
38	Etiocholanolone	0.9994	0.12-2000	0.01	0.5 (5.6%, 6.2%)	1.5/0.25	2.8/6.2
39	5α-Androstane-3α,17β-diol	0.9996	0.12-2000	0.2	0.5 (5.3%, 11%)	1.7/0.29	4.1/9.7
40	5α-Androstane-3β,17β-diol	0.9989	0.12-2000	0.02	0.5 (16%, 7.8%)	0.81/0.14	9.4/11
41	5β-Androstane-3α,17β-diol	0.9995	0.12-2000	0.09	0.5 (0.74%, 6.5%)	0.93/0.16	8.4/11
42	Cortisol	0.9991	31-10000	30	100 (-4.7%, 8.8%)	2200/310	2.9/11
43	Cortisone	0.9972	125-10000	30	100 (-0.35%, 6.6%)	370/51	4.6/8.2
44	Corticosterone	0.9987	7.8-10000	1	2 (3.2%, 10%)	90/13	4.7/9.8
45	21-Deoxycortisol	0.9991	0.49-2000	0.8	0.5 (1.3%, 12%)	1.3/0.19	11/13
46	11β-Hydroxyandrostenedione	0.9978	0.49-10000	0.9	8 (-3.8%, 11%)	470/77	3.4/6.5
47	11β-Hydroxyandrosterone	0.9998	0.12-2000	0.04	0.5 (3.3%, 10%)	50/8.2	2.4/11
48	11β-Hydroxyepiandrosterone	0.9983	0.12-2000	0.04	0.5 (6.5%, 6.7%)	3/0.49	8.2/11
49	11β-Hydroxyetiocholanolone	0.9999	0.12-2000	0.05	0.5 (7.7%, 6%)	32/5.3	2.9/12

50	Pregnenolone sulfate			5	30 (10%, 2.5%)	1600/250	1.3/7.2
51	17-Hydroxypregnenolone sulfate			1	8 (1.5%, 6.2%)	270/41	1.3/10
52	20α-Dihydropregnenolone sulfate			4	30 (3.2%, 8.2%)	8900/1400	0.76/11
53	DHEA sulfate			0.2	30 (-0.17%, 4.3%)	27000/4700	1.3/7.3
54	Androstenediol sulfate			4	30 (5.9%, 9.2%)	20000/3400	1.2/13
55	5-Androstene-3β,16α,17β-triol sulfate			0.7	8 (-0.063%, 9.4%)	310/50	2.8/12
56	Conjugated 17,20α-dihydroxy-4-pregnene-3-one			5	8 (5.2%, 7.2%)	86/13	1.5/14
57	Conjugated 20α-dihydroprogesterone			2	8 (1.9%, 12%)	18/2.9	2.8/9
58	Conjugated testosterone			2	8 (8.2%, 8%)		
59	Conjugated 5α-dihydrotestosterone			0.06	0.9 (11.3%, 7.2%)	36/6.15	6.4/9.1
60	Allopregnanolone sulfate			0.3	2 (-2.8%, 4.1%)	46/7.3	3/14
61	Isopregnanolone sulfate			0.3	8 (4%, 9%)	110/17	3.2/9.6
62	Conjugated pregnanolone			0.7	2 (0.29%, 5.5%)	200/31	1.9/9.9
63	Conjugated epipregnanolone			0.5	2 (-5.5%, 5.4%)	25/3.9	2.9/11
64	Conjugated 5α,20α-tetrahydroprogesterone			5	2 (2.3%, 8.7%)	4/0.63	8.3/6.9
65	Conjugated 5α-pregnane-3α,20α-diol			10	8 (1.4%, 8.8%)	210/33	2.1/14
66	Conjugated 5α-pregnane-3β,20α-diol			5	30 (-7.3%, 7.2%)	3200/500	5.2/8.9
67	Conjugated 5β,20α-tetrahydroprogesterone			6	30 (-9.8%, 18%)	220/35	1.1/13
68	Conjugated 5β-pregnane-3α,20α-diol			2	8 (7.4%, 6.2%)	130/20	2.5/11
69	Conjugated 5β-pregnane-3β,20α-diol			9	8 (4.9%, 7.7%)	150/24	5.6/12
70	17-Hydroxyallopregnanolone sulfate			0.5	2 (-1.6%, 7.6%)	29/4.4	4.8/7.5
71	Conjugated 17-hydroxypregnanolone			0.3	8 (-3.2%, 10%)	120/18	1.5/6.7
72	Conjugated 5α-pregnane-3α,17,20α-triol			0.2	8 (0.31%, 6%)	380/57	4.1/12.7
73	Conjugated 5α-pregnane-3β,17,20α-triol			0.2	8 (-5.8%, 17%)	34/5.1	5.4/9.7
74	Conjugated 5β-pregnane-3α,17,20α-triol			0.2	8 (1.3%, 13%)	860/130	4.3/10.5
75	Androsterone sulfate			1	8 (2%, 6.8%)	11000/1900	0.67/8.6
76	Epiandrosterone sulfate			0.9	8 (-0.81%, 8.5%)	3000/510	1.2/7.6
77	Etiocholanolone sulfate			3	8 (-2.5%, 20%)	580/100	0.63/5
78	Epietiocholanolone sulfate			0.9	8 (-1.6%, 8.2%)	310/54	0.97/8.7
79	Conjugated 5α-androstane-3α,17β-diol			1	8 (-0.64%, 5.8%)	990/170	0.98/10
80	Conjugated 5α-androstane-3β,17β-diol			0.1	8 (-1.6%, 6%)	1500/250	1.2/8.6
81	Conjugated 5β-androstane-3α,17β-diol			2	8 (2.2%, 11%)	140/24	2.5/11
82	Conjugated 5β-androstane-3β,17β-diol			2	2 (11%, 13%)	7/1.2	4.4/11
83	11β-Hydroxyandrosterone sulfate			0.2	2 (8.6%, 9%)	270/44	0.98/5.1
84	11β-Hydroxyepiandrosterone sulfate			0.2	2 (-8.1%, 8.6%)	13/2.2	6.3/6.6
85	11β-Hydroxyetiocholanolone sulfate			0.3	2 (6.2%, 7.7%)	56/9.2	1.2/11
